# Novel TDP1 Inhibitors: Disubstituted Thiazolidine-2,4-Diones Containing Monoterpene Moieties

**DOI:** 10.3390/ijms24043834

**Published:** 2023-02-14

**Authors:** Dmitry I. Ivankin, Tatyana E. Kornienko, Marina A. Mikhailova, Nadezhda S. Dyrkheeva, Alexandra L. Zakharenko, Chigozie Achara, Jóhannes Reynisson, Victor M. Golyshev, Olga A. Luzina, Konstantin P. Volcho, Nariman F. Salakhutdinov, Olga I. Lavrik

**Affiliations:** 1 N.N. Vorozhtsov Novosibirsk Institute of Organic Chemistry, Siberian Branch of the Russian Academy of Science, 9, Akademika Lavrentieva Ave., 630090 Novosibirsk, Russia; 2 Institute of Chemical Biology and Fundamental Medicine, Siberian Branch of the Russian Academy of Science, 8, Akademika Lavrentieva Ave., 630090 Novosibirsk, Russia; 3 Department of Natural Sciences, Novosibirsk State University, 630090 Novosibirsk, Russia; 4 School of Pharmacy and Bioengineering, Keele University, Hornbeam Building, Newcastle-under-Lyme, Staffordshire ST5 5BC, UK

**Keywords:** tyrosyl-DNA-phosphodiesterase 1, TDP1 inhibitors, thiazolidine-2,4-dione derivatives

## Abstract

Tyrosyl-DNA-phosphodiesterase 1 (TDP1) is a promising target for antitumor therapy; the use of TDP1 inhibitors with a topoisomerase 1 poison such as topotecan is a potential combination therapy. In this work, a novel series of 3,5-disubstituted thiazolidine-2,4-diones was synthesized and tested against TDP1. The screening revealed some active compounds with IC_50_ values less than 5 μM. Interestingly, compounds **20d** and **21d** were the most active, with IC_50_ values in the submicromolar concentration range. None of the compounds showed cytotoxicity against HCT-116 (colon carcinoma) and MRC-5 (human lung fibroblasts) cell lines in the 1–100 μM concentration range. Finally, this class of compounds did not sensitize cancer cells to the cytotoxic effect of topotecan.

## 1. Introduction

The usage of DNA repair enzyme inhibitors to fight drug-resistant tumors is a promising tactic in anticancer therapy. These inhibitors exacerbate DNA damage, leading to cancer cell death.

Topotecan and irinotecan are well-known topoisomerase 1 (TOP1) poisons that are used against many types of cancer. They stabilize the TOP1-DNA complex and result in cell death by inhibiting the religation process [1]. Tyrosyl-DNA phosphodiesterase 1 (TDP1) is an antagonist of the TOP1-DNA complex. TDP1 catalyzes the hydrolysis of the phosphodiester bond in TOP1-DNA between the 3′-phosphate of DNA and a tyrosine amino acid of TOP1 for a DNA single-strand break; this also takes place in the TOP1 poison stabilized adduct. Thus, TDP1 is thought to be responsible for the resistance of tumors to anticancer therapy [2] and by using TDP1 inhibitors, the increased efficacy of camptothecin derivatives can be achieved (topotecan and irinotecan).

**Figure 1:**
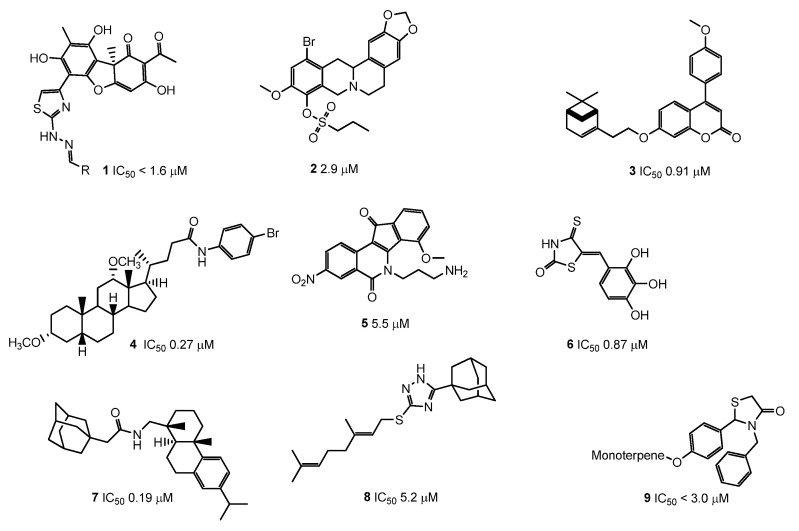
Structure of natural, semi-natural and synthetic TDP1 inhibitors.

To date, many low-molecular-weight TDP1 inhibitors have been introduced, including derivatives of natural compounds such as usnic acids **1** [3], berberines **2** [4], coumarins **3** [5] and steroids **4** [6], as well as synthetic inhibitors, such as isoquinolines **5** [7] and thiazolidines **6** [8,9] (see Figure 1).

Compounds that combine pharmacophore fragments of natural and synthetic origin also performed well, including resin acid derivatives **7** [10], and terpenoadamantanes **8** [11].

Previously, we synthesized semi-natural compounds **9** based on thiazolidin-4-one, which contained a monoterpene substituent. The obtained terpenothiazolidin-4-ones exhibit activity in the micromolar concentration range and enhance the efficacy of topotecan on cancer cell lines. We have shown that a terpene substituent is a decisive factor for inhibitory activity. However, the terpenothiazolidin-4-ones synthesized by us were obtained in the form of mixtures of either enantiomers, or, provided there is a chiral center in the terpene substituent, in the form of a mixture of diastereomers [8]. A molecular modeling investigation of these diastereomers indicated two potential binding modes in the TDP1 catalytic pocket and was not able to discern which diastereomer would preferentially bind to the pocket. Furthermore, mainstream molecular descriptors do not give insight into the different biological compatibility of optical isomers, as they share the same calculated physicochemical parameters [8]. An approach to avoid the formation of a mixture of isomers is the use of the closest analogue of thiazolidin-4-ones, thiazolidine-2,4-diones, as the nucleus for the formation of an inhibitor. The presence of thiazolidine-2,4-diones in TDP1 inhibitors (IC_50_ 0.87–55 μM) has been previously described by Pommier et al. [12]. In addition, the structure of thiazolidine-2,4-dione allows the introduction of terpene substituents at two different positions, which increases the variety of compounds obtainable for structure–activity relationship (SAR) derivation.

With the aim to identify the SAR for this class of compounds, the synthesis and biological testing of thiazolidine-2,4-dione derivatives that contained aromatic and monoterpene moieties were carried out.

## 2. Results and Discussion

### 2.1. Chemistry

At first, all the precursors required for the synthesis of 3,5-disubstituted thiazolidine-2,4-diones were obtained. (-)-Campholenic aldehyde **12g** was synthesized from (+)-α-pinene **10** by using a two-step synthesis process. The epoxidation of **10** by peracetic acid in CH_2_Cl_2_ gave compound **11** [13]. The further Meinwald rearrangement of epoxide **11** in the presence of ZnCl_2_ in benzene resulted in aldehyde **12g** [14].

**Scheme 1:**
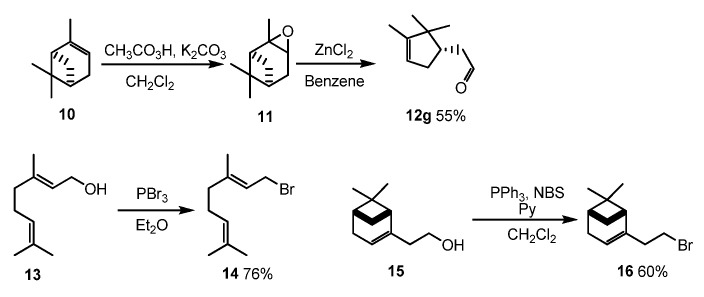
Synthesis of precursors **12g**, **14**, and **16**.

Monoterpenic bromides **14** and **16** were prepared from commercially available alcohols. Geranyl bromide **14** was synthesized from geraniol **13** using PBr_3_ [15], whereas the modification of the Appel reaction on substrate **15** gave **16 [16]** (Scheme 1).

**Scheme 2:**
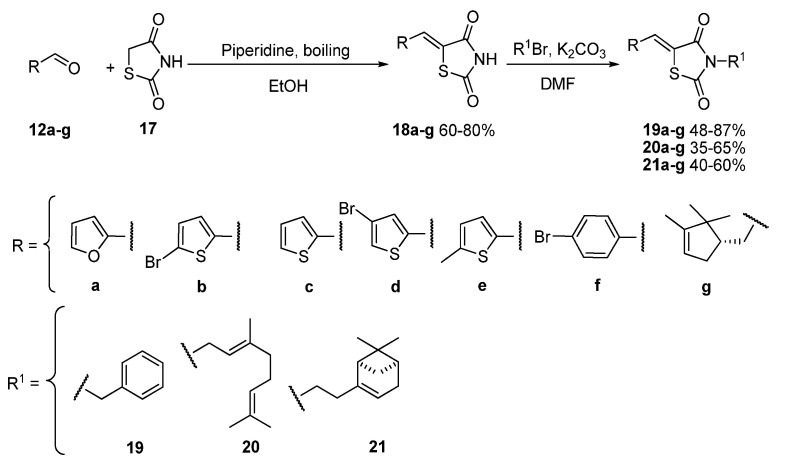
Synthesis of thiazolidine-2,4-dione derivatives.

Following this, some 3,5-disubstituted thiazolidine-2,4-diones were synthesized. At the first stage, a condensation reaction between thiazolidine-2,4-dione **17** and aldehyde **12a–g** was carried out by refluxing in ethanol for 6–20 h to obtain compounds **18a–g**. After this, monoterpenoid bromides, as well as benzyl bromide, were used for the functionalization of thiazolidine-2,4-diones to give 3,5-disubstituted derivatives **19–21** (Scheme 2).

All of the synthesized heterocyclic structures only have *Z*-exocyclic double bonds. Furthermore, the published syntheses have always indicated the preparation of a pure Z-isomer. This is thought to be related to its greater stability against an E-isomer [17,18].

For some compounds, wide yield ranges were observed. This fact may be connected to the lability of monoterpene-substituted thiazolidine-2,4-diones on silica gel in the case of column chromatography. In addition, some compounds required recrystallization after chromatography.

### 2.2. Biology

The inhibiting activity of the compounds against TDP1 was investigated using a real-time oligonucleotide biosensor assay. Human recombinant TDP1 was used as an enzyme and a 16-mer single-stranded oligonucleotide that contained both a 5′-FAM (fluorescein) fluorophore donor and a quenching 3′-BHQ1 (Black Hole Quencher 1) acceptor was used as a biosensor for in vitro screening; the results are presented in Table 1.

As can be observed, the type of substituent can affect inhibition. In the case of monosubstitution, only one structural fragment promotes binding to the enzyme—5-bromothiophene. It should be noted that in our previous work, this structural fragment exhibited the strongest binding to TDP1 [3]. However, in general, the presence of a substituent at position 3 results in binding to the enzyme and the addition of substituents at the third position of 5-bromothiophene-thiazolidine-2,4-dione did not always help to maintain activity. In the series of derivatives **18b**–**21b**, the compound with the geranyl substituent **20b** did not show affinity for the enzyme.

Most of compounds with heterocyclic fragments without substituents do not inhibite TDP1; out of eight compounds of the series of unsubstituted furans (**18a**–**21a**) and unsubstituted thiophenes (**18c**–**21c**), only one compound (**20c**) inhibits TDP1 at a micromolar concentration. The 4-bromothiophene substituent, although not an independent pharmacophore, in combination with terpene substituents at position 3 of the thiazolidine-2,4-dione, leads to compounds with the most pronounced activity; compounds **20d** and **21d** were the most active with IC_50_ values in the submicromolar range (0.65 and 0.55 μM, respectively). In addition, the presence of a terpene substituent at position 5 is promising and each of the 5-campholenidene-substituted compounds (**19g**, **20g**, **21g**) were active (IC_50_ 2–5 μM).

The analysis of the influence of position 3 substituents does not reveal a clear picture. In the series benzyl (**19a–g**), geranyl (**20a–g**), and (-)-nopyl (**21a–g**), there are approximately equal numbers of compounds that exhibit inhibitory activity (**3**, **4**, and **4** compounds, respectively). Monoterpene as well as benzyl substituents equally increase TDP1 inhibition in many cases.

**Figure 2:**
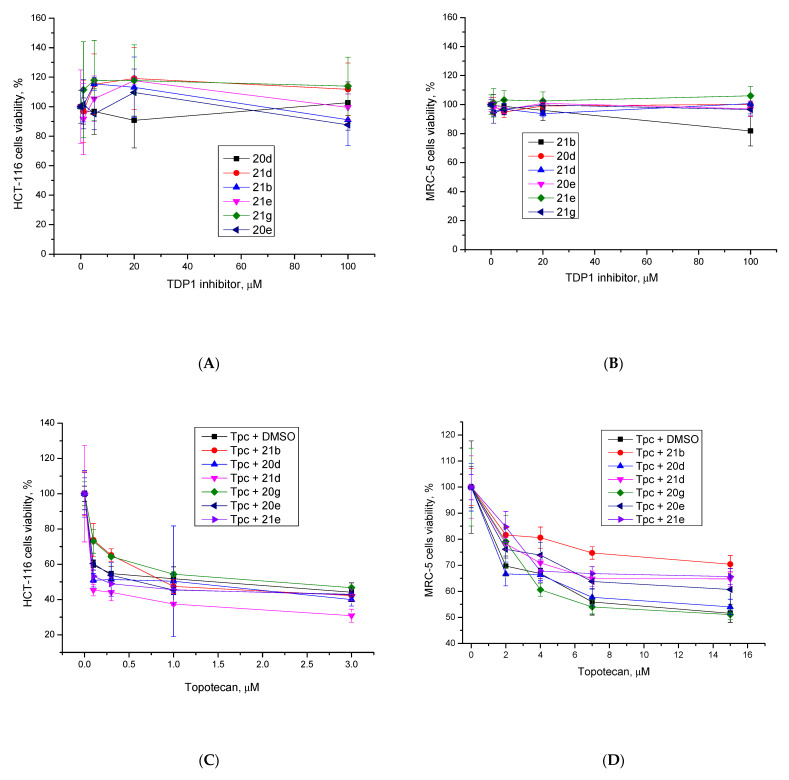
Influence of compounds on the cell line viability. The TDP1 inhibitors’ intrinsic cytotoxicity on HCT-116 cells (**A**) and MRC-5 cells (**B**)—dose-dependent action. Topotecan (Tpc) cytotoxicity in the presence of TDP1 inhibitors in HCT-116 (**C**) and MRC-5 (**D**) cells.

Since thiazolidine-2,4-dione derivatives are planned to be used in combination with known antitumor drugs, the former should not have intrinsic toxicity that could lead to side effects. We investigated the cytotoxicity of the compounds against the cell lines MRC-5 (human lung fibroblasts) and HCT-116 (colon carcinoma) and did not find any compounds that reduced cell survival using the EZ4U test in the concentration range 1 to 100 μM (data Figure 2A,B). As observed in Figure 2A,B, none of the compounds at concentrations up to 100 μM reduced cell viability by more than 20% (within experimental errors).

According the EZ4U test data the thiazolidine-2,4-diones were shown not to enhance the cytotoxic effect of topotecan at 10 μM (Figure 2C,D). In some cases, the compounds even protect cells to some extent from the action of topotecan, for example, compound **21b** protects MRC-5 cells (Figure 2D, red graph). The lack of efficacy suggests that, despite the high affinity for the enzyme and the absence of cytotoxicity, these compounds should not be considered for further development as TDP1 inhibitors. Interestingly, terpenyl-substituted thiazolidin-4-ones (**15a**, **b**, **d**, **e**, **f**) [8] had a sensitizing effect on the cytotoxicity of topotecan against HeLa tumor cells, but not on HEK293A cells of non-tumor origin. It can be speculated that the decisive factor is the use of the thiazolidine-2,4-dione fragment as a central molecular scaffold, which is a pharmacophore for many targets. In particular, thiazolidine-2,4-diones are well-known for their antidiabetic [19], antitumor [20], and anti-inflammatory [21] activities. Such promiscuity can lead to binding to a number of proteins/enzymes in cells other than TDP1.

### 2.3. In Silico

#### 2.3.1. Molecular Modeling

Twenty-eight thiazolidine-2,4-dione derivatives were docked into the binding site of TDP1 (PDB ID: 6W7K, resolution 1.70 Å) [22]. The robustness of the TDP1 docking scaffold has been previously established [23]. The scoring functions GoldScore(GS) [24], ChemScore (CS) [25,26], ChemPLP (Piecewise Linear Potential) [27] and ASP (Astex Statistical Potential) [28] in the GOLD (v2020.2.0)) docking algorithm were used. The GOLD docking algorithm is reported to be an excellent molecular modelling tool [29,30].

The binding scores for the TDP1 catalytic pocket are given in Table S1; all the ligands have reasonable values. When the scores of the active ligands were correlated against their IC_50_ values, only weak trends were observed for ASP (R^2^—0.090), ChemPLP (R^2^—0.129) and GS (R^2^—0.162). However, for CS, a correlation with R^2^—0.441 was observed (see Figure S1). Furthermore, the inactive ligands (IC_50_ > 100 μM, *n*—16) had overall lower scores for all the scoring functions, compared to the active compounds (*n*—12).

**Figure 3:**
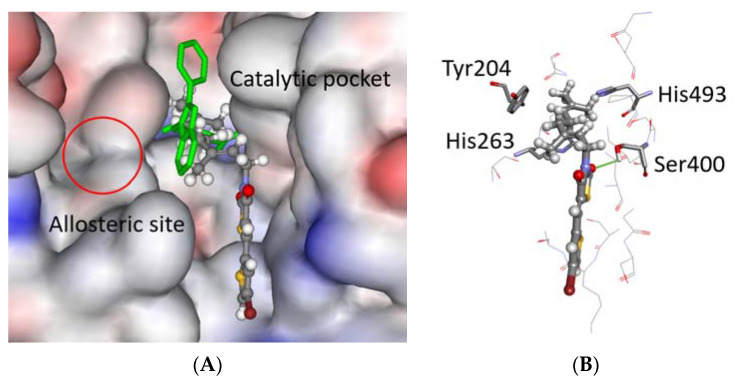
The docked pose of **21d** in the catalytic site of TDP1, as predicted by the ChemPLP scoring function. (**A**) The ligand is shown in the ball-and-stick format. The catalytic pocket is at the right-hand site of the image and the co-crystallized ligand is depicted as green sticks. The allosteric pocket is not predicted to be occupied (see circled area). The protein surface is rendered; blue depicts regions with a partial positive charge on the surface; red depicts regions with a partial negative charge and grey shows neutral areas. (**B**) The predicted configuration is depicted as balls-and-sticks; H-bonding can be observed between one of the carbonyl groups in the thiazolidinedione ring and the side chain of Ser400 (stick format). The catalytic His263 and His493 amino acid residues are shown as sticks. The adjacent amino acids (<5 Å), buttressing the ligand, are shown as lines. The amino acids’ hydrogens are not shown for clarity.

The predicted binding poses of **20b**, **21b**, **20d**, **21d**, **20g** and **21g** were investigated; no dominant binding poses were predicted by the four scoring functions. Nevertheless, all the ligands overlapped with the co-crystallized structure, as well as occupying the catalytic pocket that contained the His263 and His493 amino acid residues. The binding mode, as predicted by ChemPLP, of the most active ligand **21d** is shown in Figure 3. The aliphatic ring moiety overlaps the co-crystallized ligand, whereas the thiophene and thiazolidinedione rings are in a groove with the latter ring system, forming an H-bond from one of its carbonyl groups with the side chain of the Ser400 amino acid residue (see Figure 3B).

Molecular dynamics simulations have suggested that TDP1 inhibitors occupy an allosteric binding pocket next to the catalytic site, as shown in Figure 3A [31]. Molecular modelling and structural activity relationship studies of usnic acid derivatives corroborate the existence of the allosteric site and its occupancy being beneficial to the overall binding efficacy [23]. None of the ligands were predicted to occupy the allosteric pocket, explaining the relatively modest binding affinity observed.

The inconsistency of the scoring functions that predicted a dominant pose indicates that the thiazolidine-2,4-diones are not particularly selective for TDP1; this is in line with the observation that the ligands do not potentiate topotecan in cancer cells.

#### 2.3.2. Chemical Space

The calculated molecular descriptors MW (molecular weight), log *P* (water–octanol partition coefficient), HD (hydrogen bond donors), HA (hydrogen bond acceptors), PSA (polar surface area) and RB (rotatable bonds) are given in Table S2. The values of the molecular descriptors lie within lead-like chemical space for HD and HA and in lead- and drug-like space for RB, MW and PSA; finally, log *P* spans all three definitions of chemical space (for the definition of lead-like, drug-like and known drug space (KDS) regions, see ref. [32] and Table S3). Some of the log *P* values are as high as 6.5, located at the border of KDS. No correlations were found between the IC_50_ values of the active ligands and their descriptors; however, the log *P* values of the active ligands tended to be higher than for the inactive ones (active: $\bar{x}$ = 5.2 ± 1.2 vs. inactive: $\bar{x}$ = 3.7 ± 1.7). The higher log *P* of the active compounds supports the previous findings from the cell bases studies and molecular modelling that entropy-driven promiscuous binding to various components of the cell occurs, not leading to any biological effect.

The Known Drug Indexes (KDIs) for the ligands were calculated to gauge the balance of the molecular descriptors (MW, *log* P, HD, HA, PSA and RB). This method is based on the analysis of drugs in clinical use, i.e., the statistical distribution of each descriptor is fitted to a Gaussian function and normalized to 1, resulting in a weighted index. Both the summation of the indexes (KDI_2a_) and multiplication (KDI_2b_) methods were used [33], as shown for KDI_2a_ in Equation (1) and for KDI_2b_ in Equation (2); the numerical results are given in Table S2.

KDI_2a_ = I_MW_ + I_log P_ + I_HD_ + I_HA_ + I_RB_ + I_PSA_ (1)

KDI_2b_ = I_MW_ × I_log P_ × I_HD_× I_HA_ × I_RB_ × I_PSA_ (2)

The KDI_2a_ values for the ligands range from 3.97 to 5.00, with a theoretical maximum of 6 and an average of 4.08 (±1.27) for known drugs. The KDI_2b_ values range from 0.05 to 0.31, with a theoretical maximum of 1 and with a KDS average of 0.18 (±0.20). The range in the KDI values is quite high, as some of the ligands have high log *P* values.

## 3. Materials and Methods

### 3.1. Chemistry Section

The ^1^H and ^13^C NMR spectra for the solutions of the compounds in CDCl_3_ were recorded on a Bruker AV-400 spectrometer (Bruker Corporation, Billerica, MA, USA) (400.13 and 100.61 MHz, respectively). The residual signals of the solvent were used as references (*δ*_H_ 7.24, *δ*_C_ 76.9). The mass spectra (70 eV) were recorded on a DFS Thermo Scientific high-resolution mass spectrometer (Thermo Fisher Scientific, Waltham, MA, USA). Thin-layer chromatography was performed on TLC Silica gel 60 (F_254_, Merck, Germany). Merck silica gel (63–200 μm) was used for the column chromatography. Circular dichroism spectra of the samples were registered using a quartz cell (1 mm path length) on a JASCO J-600 spectrophotometer (JASCO, Tokyo, Japan). All CD spectra were obtained in 5 replicates in the spectral range 200–350 nm at 1 nm resolution and 2 nm bandwidth, with a response time of 1 s and sensitivity of 10 mdeg. The averaged values are presented in Figure S55. The samples were dissolved in methanol and the concentration of all the studied samples was 1 mM. The UV–Vis absorption spectra of some compounds (in a final concentration of 0.1 mM) were recorded with a TDP1 reaction buffer (50 mM Tris-HCl, pH 8.0, 50 mM NaCl, and 7 mM β-mercaptoethanol), using a POLARstar OPTIMA fluorimeter (BMG LABTECH, GmbH, Ortenberg, Germany). The data are presented using MARS Data Analysis 2.0 (BMG LABTECH), as shown in Figures S56–S62.

All solvents were purified by general methods before the experiments. All synthetic reagents were purchased from Acros Organics (Geel, Belgium).

The atom numerations in the compounds are provided for the assignment of signals in the NMR spectra and are different from the atom numerations in the nomenclature name.

#### 3.1.1. Synthesis of Compounds **12g, 14**, and **16**

*(-)-(1S)-2,2,3-Trimethylcyclopent-3-enylethanal* ((-)-campholenic aldehyde) **12g** was synthesized from (+)-α-pinene **10** based on [13,14]. A mixture of peracetic acid (248 mmol) and anhydrous Na_2_CO_3_ (207 mmol) was added to a stirred solution of (+)-α-pinene (207 mmol) in CH_2_Cl_2_ (200 mL) at 0 °C for 45 min. Then, the mixture was allowed to warm to room temperature, followed by stirring for about 5 h. After this, the solution was washed with water (3 × 100 mL), brine (2 × 100 mL) and dried over anhydrous Na_2_SO_4_ and filtered. The filtrate was evaporated and the product was used without further purification.

A solution of epoxide **11** was added to the suspension of anhydrous ZnCl_2_ (22 mmol) in toluene (600 mL). The mixture was stirred at room temperature for about 36 h. Then, the mixture was washed with 50% acetic acid (15 mL) and bicarbonate solution (5%, 15 mL). The extract was dried over anhydrous Na_2_SO_4_ and filtered. After removing the toluene, the product was purified by column chromatography on silica gel. The eluent was hexane with diethyl ether (10/1) with a yield of 74%.

*(E)-1-Bromo-3,7-dimethylocta-2,6-diene* (geranyl bromide) **14** was synthesized from geraniol **13** according to [15]. Geraniol **13** (3.24 mmol) and phosphorus tribromide (1.36 mmol) were stirred in dry diethyl ether at −15 °C for 3 h. Then, the solution was quenched in distilled water with ice. After the ice melted, the mixture was poured into a separatory funnel. The organic phase was washed with sodium bicarbonate solution (5%) and brine in turn, and then it was dried with anhydrous Na_2_SO_4_ and filtered. The filtrate was evaporated and the product was used without further purification (yield 76%).

*(1R,5S)-2-(2-Bromoethyl)-6,6-dimethylbicyclo [3.1.1]hept-2-ene* ((-)-nopyl bromide) **16** was synthesized from (-)-nopol **15** according to [16]. Triphenylphosphine (23 mmol) was dissolved in 23 mL of dry dichloromethane. *N*-bromosuccinimide (23 mmol) was added to this solution in small portions over 5 min under an ice-water bath. Later, the ice-water bath was removed and the mixture was stirred at room temperature for 30 min. Then, pyridine (1 mL) was added and the color turned to reddish-brown. (–)-Nopol **15** (12 mmol) was added to the mixture dropwise over 10 min. The mixture was stirred overnight at room temperature. After this, the mixture was diluted with hexane (40 mL) and filtered through a silica gel plug. In order to obtain a high yield, the reaction flask was washed three times with ethyl acetate/hexane (25/25 mL) and filtered through the silica gel plug. Later, it was concentrated in vacuo and the solid powder was stirred in hexane (100 mL) for 15 min. Then, it was filtered and concentrated in vacuo. The crude residue (yellowish oil) was purified by flash chromatography (hexane) to obtain (–)-nopyl bromide (yield 60%).

#### 3.1.2. Synthesis of 5-Substituted Thiazolidine-2,4-Diones

A mixture of aldehyde (13.2 mmol), thiazolidine-2,4-dione (13.2 mmol), and a catalytic amount of piperidine in 100 mL of ethanol was heated under reflux and stirred for 6–20 h. After this, ethanol was evaporated. The products were purified by recrystallization from ethanol, except for **18g**, which was purified by column chromatography on silica gel. The eluent was methylene chloride with a methanol gradient of up to 3% by vol.

*(Z)-5-(furan-2-ylmethylene)thiazolidine-2,4-dione***18a**.

Yield 68%. Brown solid substance. The spectrum data were similar to those mentioned here [34].

*(Z)-5-((5-bromothiophen-2-yl)methylene)thiazolidine-2,4-dione***18b**.

Yield 60%. Brown solid substance. The spectrum data were similar to those mentioned here [18].

*(Z)-5-(thiophen-2-ylmethylene)thiazolidine-2,4-dione***18c**.

Yield 60%. Pale yellow solid substance. The spectrum data were similar to those mentioned here [35].

*(Z)-5-((4-bromothiophen-2-yl)methylene)thiazolidine-2,4-dione***18d**.

Yield 72%. Pale yellow solid substance. The spectrum data were similar to those mentioned here [36].

*(Z)-5-((5-methylthiophen-2-yl)methylene)thiazolidine-2,4-dione***18e** (Figures S1–S3).

Yield 70%. Pale yellow solid substance. M.p. 210 °C. HRMS: 224.9911 [M]^+^; calcd. 224.9913 (C_9_H_7_O_2_N_1_^32^S_2_)^+^. ^1^H-NMR (400 MHz, DMSO-d_6_) (ppm) δ: 2.53 (s, 3H, H-9), 6.97 (d, 1H, *J*_7,6_ = 3.6 Hz, H-7), 7.46 (d, *J*_6,7_ = 3.6 Hz, 1H, H-6), 7.93 (s, 1H, H-4). ^13^C-NMR (125 MHz, DMSO-d_6_) (ppm) δ: 167.32 (s, C-1), 167.09 (s, C-2), 119.39 (s, C-3), 125.53 (d, C-4), 135.25 (d, C-5), 127.68 (d, C-6), 135.23 (d, C-7), 147.96 (s, C-8), 15.54 (k, C-9).

*(Z)-5-(4-bromobenzylidene)thiazolidine-2,4-dione***18f**.

Yield 65%. Pale yellow solid substance. The spectrum data were similar to those mentioned here [35].

*(S,Z)-5-(2-(2,2,3-Trimethylcyclopent-3-enyl)ethylene)thiazolidine-2,4-dione***18g**.

Yield 80%. White solid substance. The data spectra were similar to those mentioned here [37].

#### 3.1.3. Synthesis of 3,5-Disubstituted Thiazolidine-2,4-Diones

Potassium carbonate (3.60 mmol) was added to a solution of 5-substituted thiazolidine-2,4-dione (1.20 mmol) in 5 mL of dry DMF at room temperature. After stirring the reaction mixture for 30 min, organic bromide (1.20 mmol) was added, and then the mixture was stirred at room temperature for 4 h. After this, the reaction was quenched by adding distilled water and the product was extracted with ethyl acetate (3 × 10 mL). The extracts were washed with brine, dried with Na_2_SO_4_, and evaporated. The product was purified by column chromatography on silica gel using hexane as an eluent, with an ethyl acetate gradient of up to 25% by vol. or/and by recrystallization from hexane/ethyl acetate.

*(Z)-3-Benzyl-5-(furan-2-ylmethylene)thiazolidine-2,4-dione***19a**.

Yield 94%. Pale yellow solid substance. The spectrum data were similar to those mentioned here [38].

*(Z)-3-((E)-3,7-dimethylocta-2,6-dienyl)-5-(furan-2-ylmethylene)thiazolidine-2,4-dione***20a**.

Yield 56%. Yellowish solid substance. The spectrum data were similar to those mentioned here [37].

*(Z)-3-(2-((1R,5S)-6,6-dimethylbicyclo[3.1.1]hept-2-en-2-yl)ethyl)-5-(thiophen-2-ylmethylene)thiazolidine-2,4-dione***21a** (Figures S4–S6).

Yield 62%. Pale pink solid substance. M.p. 117 °C. HRMS: 343.1237 [M]^+^; calcd. 343.1234 (C_19_H_21_O_2_N_1_^32^S_2_)^+^. ${\left[\alpha \right]}_{589}^{25}$ = −3.08 (c = 0.26, CDCl_3_). ^1^H-NMR (500 MHz, CDCl_3_) (ppm) δ: 0.80 (s, 3H, H-18), 1.08 (d, 1H, *J*_17_^a^_,17_^b^ = 8.6 Hz, H-17^a^), 1.26 (s, 3H, H-19), 2.00–2.12 (m, 2H, H-14, H-16), 2.15–2.28 (m, 3H, H-13, H-17^b^), 2.25–2.50 (m, 2H, H-10), 3.64–3.84 (m, 2H, H-9), 5.30 (m, 1H, H-12), 6.54 (dd, *J*_7,6_ = 3.4 Hz, *J*_7,8_ = 1.7 Hz, H-7), 6.75 (d, *J*_6,7_ = 3.4 Hz, H-6), 7.72 (d, 1H, *J*_8,7_ = 1.7 Hz, H-8), 7.64 (s, 1H, H-4). ^13^C-NMR (100 MHz, CDCl_3_) (ppm) δ: 168.32 (s, C-1), 165.80 (s, C-2), 119.14 (s, C-3), 117.31 (d, C-4), 149.67 (s, C-5), 119.13 (d, C-6), 112.88 (d, C-7), 146.08 (s, C-8), 40.00 (t, C-9), 34.41 (t, C-10), 143.88 (s, C-11), 119.13 (d, C-12), 31.16 (t, C-13), 40.44 (d, C-14), 37.80 (s, C-15), 45.27 (d, C-16), 34.41 (t, C-17), 26.04 (k, C-18), 20.92 (k, C-19).

*(Z)-3-Benzyl-5-((5-bromothiophen-2-yl)methylene)thiazolidine-2,4-dione***19b** (Figures S7–S9).

Yield 87%. White solid substance. M.p. 166 °C. HRMS: 378.9327 [M]^+^; calcd. 378.9331 (C_15_H_10_O_2_N_1_^79^Br_1_^32^S_2_)^+^. ^1^H-NMR (400 MHz, CDCl_3_) (ppm) δ: 4.87 (s, 2H, H-9), 7.10–7.13 (m, 2H, H-6, H-7), 7.26–7.44 (m, 5H, H-11, H-12, H-13, H-14, H-15), 7.89 (s, 1H, H-4). ^13^C-NMR (100 MHz, CDCl_3_) (ppm) δ: 166.57 (s, C-1), 165.58 (s, C-2), 119.90 (s, C-3), 125.44 (d, C-4), 139.05 (s, C-5), 133.32 (d, C-6), 131.43 (d, C-7), 119.75 (s, C-8), 45.38 (t, C-9), 134.91 (s, C-10), 128.64 (d, C-11, C-15), 128.75 (d, C-12, C-14), 128.19 (d, C-13).

*(Z)-5-((5-bromothiophen-2-yl)methylene)-3-((E)-3,7-dimethylocta-2,6-dienyl)thiazolidine-2,4-dione***20b** (Figures S10–S12).

Yield 65%. White solid substance. M.p. 73 °C. HRMS: 425.0106 [M]^+^; calcd. 425.0113 (C_18_H_20_O_2_N_1_^79^Br_1_^32^S_2_)^+^. ^1^H-NMR (400 MHz, CDCl_3_) (ppm) δ: 1.56 (s, 3H, H-17), 1.64 (s, 3H, H-16), 1.78 (s, 3H, H-18), 1.89–2.10 (m, 4H, H-12, H-13), 4.30 (d, 2H, *J*_9,10_ = 7.3 Hz, H-9), 5.02 (t, 1H, *J*_14,13_ = 6.9 Hz, H-14), 5.20 (t, 1H, *J*_10,9_ = 6.9 Hz, H-10), 7.10–7.13 (m, 2H, H-6, H-7), 7.88 (s, 1H, H-4). ^13^C-NMR (100 MHz, CDCl_3_) (ppm) δ: 166.32 (s, C-1), 165.53 (s, C-2), 119.52 (s, C-3), 124.84 (d, C-4), 139.13 (s, C-5), 132.98 (d, C-6), 131.29 (d, C-7), 120.17 (s, C-8), 39.29 (t, C-9), 116.38 (d, C-10), 141.94 (s, C-11), 39.85 (t, C-12), 26.04 (t, C-13), 123.47 (d, C-14), 131.63 (s, C-15), 25.43 (k, C-16), 17.45 (k, C-17), 16.23 (k, C-18).

*(Z)-5-((5-bromothiophen-2-yl)methylene)-3-(2-((1R,5S)-6,6-dimethylbicyclo[3.1.1]hept-2-en-2-yl)ethyl)thiazolidine-2,4-dione***21b** (Figures S13–S15).

Yield 55%. Yellowish solid substance. M.p. 84 °C. HRMS: 437.0112 [M]^+^; calcd. 437.0113 (C_19_H_20_O_2_N_1_^79^Br_1_^32^S_2_)^+^. ^1^H-NMR (400 MHz, CDCl_3_) (ppm) δ: 0.80 (s, 3H, H-18), 1.08 (d, 1H, *J*_17_^a^_,17_^b^ = 8.6 Hz, H-17^a^), 1.26 (s, 3H, H-19), 2.01–2.11 (m, 2H, H-14, H-16), 2.15–2.22 (m, 2H, H-13), 2.25–2.50 (m, 3H, H-17^b^, H-10), 3.64–3.84 (m, 2H, H-9), 5.30 (m, 1H, H-12), 7.10–7.13 (m, 2H, H-6, H-7), 7.88 (s, 1H, H-4). ^13^C-NMR (100 MHz, CDCl_3_) (ppm) δ: 166.36 (s, C-1), 165.51 (s, C-2), 119.52 (s, C-3), 124.83 (d, C-4), 139.08 (s, C-5), 133.00 (d, C-6), 131.26 (d, C-7), 119.52 (s, C-8), 40.34 (t, C-9), 34.35 (t, C-10), 143.71 (s, C-11), 119.23 (d, C-12), 31.13 (t, C-13), 40.40 (d, C-14), 37.77 (s, C-15), 45.24 (d, C-16), 31.52 (t, C-17), 26.00 (k, C-18), 20.88 (k, C-19).

*(Z)-3-Benzyl-5-(thiophen-2-ylmethylene)thiazolidine-2,4-dione***19c** (Figures S16–S18)

Yield 50%. Yellowish solid substance. M.p. 165 °C. HRMS: 301.0221 [M]^+^; calcd. 301.0226 (C_15_H_11_O_2_N_1_^32^S_2_)^+^. ^1^H-NMR (400 MHz, CDCl_3_) (ppm) δ: 4.87 (s, 2H, H-9), 7.15 (t, 1H, *J*_7,8_ = 4.4 Hz, H-7), 7.25–7.46 (m, 5H, H-11, H-12, H-13, H-14, H-15), 7.36 (d, 1H, *J*_6,7_ = 3.7 Hz, H-6), 7.62 (d, 1H, *J*_8,7_ = 4.8 Hz, H-8), 8.04 (s, 1H, H-4). ^13^C-NMR (100 MHz, CDCl_3_) (ppm) δ: 167.09 (s, C-1), 165.77 (s, C-2), 119.21 (s, C-3), 126.47 (d, C-4), 137.50 (s, C-5), 133.26 (d, C-6), 128.60 (d, C-7), 131.88 (d, C-8), 45.22 (t, C-9), 135.01 (s, C-10), 128.47 (d, C-11, C-15), 128.71 (d, C-12, C-14), 128.11 (d, C-13).

*(Z)-3-((E)-3,7-dimethylocta-2,6-dienyl)-5-(thiophen-2-ylmethylene)thiazolidine-2,4-dione***20c** (Figures S19–S21).

Yield 35%. Yellowish solid substance. M.p. 92 °C. HRMS: 347.1003 [M]^+^; calcd. 347.1008 (C_18_H_21_O_2_N_1_^32^S_2_)^+^. ^1^H-NMR (400 MHz, CDCl_3_) (ppm) δ: 1.59 (s, 3H, H-17), 1.67 (s, 3H, H-16), 1.81 (s, 3H, H-18), 1.96–2.15 (m, 4H, H-12, H-13), 4.34 (d, 2H, *J*_9,10_ = 7.3 Hz, H-9), 5.05 (t, 1H, *J*_14,13_ = 6.9 Hz, H-14), 5.25 (t, 1H, *J*_10,9_ = 6.9 Hz, H-10), 7.19 (t, 1H, *J*_7,6_ = 4.2 Hz, H-7), 7.39 (d, 1H, *J*_6,7_ = 3.0 Hz, H-6), 7.66 (d, 1H, *J*_8,7_ = 4.5 Hz, H-8), 8.01 (s, 1H, H-4). ^13^C-NMR (100 MHz, CDCl_3_) (ppm) δ: 166.96 (s, C-1), 165.83 (s, C-2), 119.67 (s, C-3), 125.99 (d, C-4), 137.64 (s, C-5), 133.06 (d, C-6), 128.42 (d, C-7), 131.66 (d, C-8), 39.37 (t, C-9), 116.56 (d, C-10), 141.85 (s, C-11), 39.79 (t, C-12), 26.12 (t, C-13), 123.56 (d, C-14), 131.71 (s, C-15), 25.54 (k, C-16), 17.55 (k, C-17), 16.32 (k, C-18).

*(Z)-3-(2-((1R,5S)-6,6-dimethylbicyclo[3.1.1]hept-2-en-2-yl)ethyl)-5-(thiophen-2-ylmethylene)thiazolidine-2,4-dione***21c** (Figures S22–S24).

Yield 40%. White solid substance. M.p. 101 °C. HRMS: 359.1007 [M]^+^; calcd. 359.1008 (C_19_H_21_O_2_N_1_^79^Br_1_^32^S_2_)^+^. ${\left[\alpha \right]}_{589}^{25}$ = −3.87 (c = 0.52, CDCl_3_). ^1^H-NMR (400 MHz, CDCl_3_) (ppm) δ: 0.80 (s, 3H, H-18), 1.08 (d, 1H, *J*_17_^a^_,17_^b^ = 8.6 Hz, H-17^a^), 1.26 (s, 3H, H-19), 2.00–2.11 (m, 2H, H-14, H-16), 2.15–2.22 (m, 2H, H-13), 2.25–2.50 (m, 3H, H-17^b^, H-10), 3.64–3.84 (m, 2H, H-9), 5.30 (m, 1H, H-12), 7.16 (dd, 1H, *J*_7,6_ = 5.3 Hz, *J*_7,8_ = 3.8 Hz, H-7), 7.38 (dt, 1H, *J*_6,7_ = 3.5 Hz, J < 1.0 Hz, H-6), 7.63 (dt, 1H, *J*_8,7_ = 5.0 Hz, *J*_8,6_ = 1.0 Hz, H-8), 7.80 (s, 1H, H-4). ^13^C-NMR (100 MHz, CDCl_3_) (ppm) δ: 166.73 (s, C-1), 165.53 (s, C-2), 119.13 (s, C-3), 127.71 (d, C-4), 137.32 (s, C-5), 132.83 (d, C-6), 131.41 (d, C-7), 131.40 (s, C-8), 40.03 (t, C-9), 34.18 (t, C-10), 143.54 (s, C-11), 119.13 (d, C-12), 30.92 (t, C-13), 40.14 (d, C-14), 37.57 (s, C-15), 44.96 (d, C-16), 31.31 (t, C-17), 25.80 (k, C-18), 20.70 (k, C-19).

*(Z)-3-Benzyl-5-((4-bromothiophen-2-yl)methylene)thiazolidine-2,4-dione***19d** (Figures S25–S27).

Yield 48%. Yellowish solid substance. M.p. 189 °C. HRMS: 378.9330 [M]^+^; calcd. 378.9331 (C_15_H_10_O_2_N_1_^79^Br_1_^32^S_2_)^+^. ^1^H-NMR (400 MHz, CDCl_3_) (ppm) δ: 4.87 (s, 2H, H-9), 7.24–7.25 (m, 1H, H-6), 7.26–7.44 (m, 5H, H-11, H-12, H-13, H-14, H-15), 7.49 (m, 1H, H-8), 7.91 (s, 1H, H-4). ^13^C-NMR (100 MHz, CDCl_3_) (ppm) δ: 166.49 (s, C-1), 165.50 (s, C-2), 112.02 (s, C-3), 138.32 (d, C-4), 138.31 (s, C-5), 124.71 (d, C-6), 121.05 (s, C-7), 134.50 (d, C-8), 45.42 (t, C-9), 134.83 (s, C-10), 128.64 (d, C-11, C-15), 128.81 (d, C-12, C-14), 128.23 (d, C-13).

*(Z)-5-((4-bromothiophen-2-yl)methylene)-3-((E)-3,7-dimethylocta-2,6-dienyl)thiazolidine-2,4-dione***20d** (Figures S28–S30).

Yield 45%. Yellowish solid substance. M.p. 141 °C. HRMS: 425.0107 [M]^+^; calcd. 425.0113 (C_18_H_20_O_2_N_1_^32^S_2_)^+^. ^1^H-NMR (400 MHz, CDCl_3_) (ppm) δ: 1.54 (s, 3H, H-17), 1.63 (s, 3H, H-16), 1.78 (s, 3H, H-18), 1.91–2.11 (m, 4H, H-12, H-13), 4.30 (d, 2H, *J*_9,10_ = 7.4 Hz, H-9), 5.01 (t, 1H, *J*_14,13_ = 6.9 Hz, H-14), 5.20 (t, 1H, *J*_10,9_ = 6.9 Hz, H-10), 7.23 (m, 1H, H-7), 7.48 (m, 1H, H-6), 7.88 (s, 1H, H-4). ^13^C-NMR (100 MHz, CDCl_3_) (ppm) δ: 166.39 (s, C-1), 165.58 (s, C-2), 112.00 (s, C-3), 128.17 (d, C-4), 138.50 (s, C-5), 124.29 (d, C-6), 142.17 (s, C-7), 134.38 (d, C-8), 39.42 (t, C-9), 134.40 (d, C-10), 142.17 (s, C-11), 40.01 (t, C-12), 26.16 (t, C-13), 123.61 (d, C-14), 131.79 (s, C-15), 25.63 (k, C-16), 17.63 (k, C-17), 16.40 (k, C-18).

*(Z)-5-((4-bromothiophen-2-yl)methylene)-3-(2-((1R,5S)-6,6-dimethylbicyclo[3.1.1]hept-2-en-2-yl)ethyl)thiazolidine-2,4-dione***21d** (Figures S31–S33).

Yield 50%. Yellowish solid substance. This compound decomposes without melting before 370 °C. HRMS: 437.0106 [M]^+^; calcd. 437.0113 (C_19_H_20_O_2_N_1_^79^Br_1_^32^S_2_)^+^. ${\left[\alpha \right]}_{589}^{25}$ = −1.35 (c = 0.57, CDCl_3_). ^1^H-NMR (300 MHz, CDCl_3_) (ppm) δ: 0.80 (s, 3H, H-18), 1.08 (d, 1H, *J*_17_^a^_,17_^b^ = 8.6 Hz, H-17^a^), 1.26 (s, 3H, H-19), 2.00–2.11 (m, 2H, H-14, H-16), 2.15–2.22 (m, 2H, H-13), 2.25–2.50 (m, 3H, H-17^b^, H-10), 3.64–3.84 (m, 2H, H-9), 5.30 (m, 1H, H-12), 7.25 (m, 1H, H-7), 7.49 (m, 1H, H-8), 7.89 (s, 1H, H-4). ^13^C-NMR (125 MHz, CDCl_3_) (ppm) δ: 166.43 (s, C-1), 165.54 (s, C-2), 111.95 (s, C-3), 128.14 (d, C-4), 138.39 (s, C-5), 124.29 (d, C-6), 138.4 (s, C-7), 134.40 (s, C-8), 40.50 (t, C-9), 34.44 (t, C-10), 143.72 (s, C-11), 119.40 (d, C-12), 31.21 (t, C-13), 40.40 (d, C-14), 37.88 (s, C-15), 45.20 (d, C-16), 31.62 (t, C-17), 26.09 (k, C-18), 21.00 (k, C-19).

*(Z)-3-Benzyl-5-((5-methylthiophen-2-yl)methylene)thiazolidine-2,4-dione***19e** (Figures S34–S36).

Yield 86%. White solid substance. M.p. 144 °C. HRMS: 315.0379 [M]^+^; calcd. 315.0382 (C_16_H_13_O_2_N_1_^32^S_2_)^+^. ^1^H-NMR (400 MHz, CDCl_3_) (ppm) δ: 2.70 (s, 3H, H-16), 5.01 (s, 2H, H-9), 6.97 (dd, 1H, *J*_7,6_ = 3.7 Hz, *J*_7,16_ = 1.0 Hz, H-7), 7.33 (d, *J*_6,7_ = 3.6 Hz, 1H, H-6), 7.35–7.60 (m, 5H, H-11, H-12, H-13, H-14, H-15), 8.11 (s, 1H, H-4). ^13^C-NMR (125 MHz, CDCl_3_) (ppm) δ: 167.37 (s, C-1), 165.87 (s, C-2), 117.40 (s, C-3), 126.95 (d, C-4), 135.48 (d, C-5), 127.12 (d, C-6), 134.13 (d, C-7), 148.30 (s, C-8), 45.12 (t, C-9), 135.10 (s, C-10), 128.56 (d, C-11, C-15), 128.70 (d, C-12, C-14), 128.06 (d, C-13), 15.87 (k, C-16).

*(Z)-3-((E)-3,7-dimethylocta-2,6-dienyl)-5-((5-methylthiophen-2-yl)methylene)-thiazolidine-2,4-dione***20e** (Figures S37–S39).

Yield 60%. Pale yellow solid substance. M.p. 54 °C. HRMS: 361.1162 [M]^+^; calcd. 361.1165 (C_19_H_23_O_2_N_1_^32^S_2_)^+^. ^1^H-NMR (300 MHz, CDCl_3_) (ppm) δ: 1.55 (s, 3H, H-17), 1.63 (s, 3H, H-16), 1.78 (s, 3H, H-18), 1.89–2.13 (m, 4H, H-12, H-13), 2.55 (s, 3H, H-19), 4.29 (d, 2H, *J*_9,10_ = 7.3 Hz, H-9), 5.02 (t, 1H, *J*_14,13_ = 6.5 Hz, H-14), 5.21 (t, 1H, *J*_10,9_ = 6.5 Hz, H-10), 6.81 (d, *J*_7,6_ = 2.9 Hz, 1H, H-7), 7.17 (d, 1H, *J*_6,7_ = 2.9 Hz, H-6), 7.93 (s, 1H, H-4). ^13^C-NMR (75 MHz, CDCl_3_) (ppm) δ: 167.23 (s, C-1), 165.92 (s, C-2), 117.93 (s, C-3), 127.05 (d, C-4), 135.64 (s, C-5), 133.86 (d, C-6), 126.45 (d, C-7), 148.00 (s, C-8), 39.36 (t, C-9), 116.67 (d, C-10), 141.69 (s, C-11), 39.70 (t, C-12), 26.12 (t, C-13), 123.58 (d, C-14), 131.68 (s, C-15), 25.53 (k, C-16), 17.54 (k, C-17), 15.84 (k, C-18).

*(Z)-3-(2-((1R,5S)-6,6-dimethylbicyclo[3.1.1]hept-2-en-2-yl)ethyl)-5-((5-methylthiophen-2-yl)methylene)thiazolidine-2,4-dione***21e** (Figures S40–S42).

Yield 60%. Pale yellow solid substance. M.p. 59 °C. HRMS: 373.1159 [M]^+^; calcd. 373.1165 (C_20_H_23_O_2_N_1_^32^S_2_)^+^. ${\left[\alpha \right]}_{589}^{25}$ = −1.69 (c = 0.59, CDCl_3_). ^1^H-NMR (400 MHz, CDCl_3_) (ppm) δ: 0.96 (s, 3H, H-18), 1.24 (d, 1H, *J*_17_^a^_,17_^b^ = 8.6 Hz, H-17^a^), 1.41 (s, 3H, H-19), 2.01–2.11 (m, 2H, H-14, H-16), 2.15–2.22 (m, 2H, H-13), 2.25–2.50 (m, 3H, H-17^b^, H-10), 2.71 (s, 3H, H-20), 3.70–3.84 (m, 2H, H-9), 5.47 (m, 1H, H-12), 6.98 (dd, *J*_7,6_ = 3.7 Hz, *J*_7,20_ = 1.0 Hz, 1H, H-7), 7.34 (d, *J*_6,7_ = 3.7 Hz, 1H, H-6), 8.09 (s, 1H, H-4). ^13^C-NMR (75 MHz, CDCl_3_) (ppm) δ: 167.00 (s, C-1), 165.63 (s, C-2), 117.41 (s, C-3), 126.76 (d, C-4), 135.31 (s, C-5), 133.62 (d, C-6), 126.16 (d, C-7), 147.74 (s, C-8), 40.16 (t, C-9), 34.20 (t, C-10), 143.61 (s, C-11), 118.88 (d, C-12), 30.91 (t, C-13), 49.95 (d, C-14), 37.56 (s, C-15), 44.98 (d, C-16), 31.30 (t, C-17), 25.80 (k, C-18), 20.69 (k, C-19), 15.55 (k, C-20).

*(Z)3-benzyl-5-(4-bromobenzylidene)thiazolidine-2,4-dione***19f** (Figures S43–S45).

Yield 90%. White solid substance. M.p. 150 °C. HRMS: 372.9771 [M]^+^; calcd. 372.8797 (C_17_H_12_O_2_N_1_^79^Br_1_^32^S_1_)^+^. ^1^H-NMR (500 MHz, CDCl_3_) (ppm) δ: 4.88 (s, H-11), 7.25–7.36 (m, 5H, H-13, H-14, H-15, H-16, H-17), 7.42 (d, 2H, *J*_7,6_ = 8.5 Hz, H-7, H-9), 7.57 (d, 2H, *J*_6,7_ = 8.5 Hz, H-6, H-10), 7.80 (s, 1H, H-4). ^13^C-NMR (125 MHz, CDCl_3_) (ppm) δ: 167.18 (s, C-1), 165.83 (s, C-2), 122.05 (s, C-3), 132.46 (d, C-4), 131.91 (s, C-5), 131.29 (d, C-6, C-10), 132.38 (d, C-7, C-9), 124.97 (s, C-8), 45.23 (t, C-11), 134.85 (s, C-12), 128.63 (d, C-13, C-17), 128.77 (d, C-14, C-16), 127.88 (d, C-15).

*(Z)-5-(4-bromobenzylidene)-3-((E)-3,7-dimethylocta-2,6-dienyl)thiazolidine-2,4-dione***20f** (Figures S46–S48).

Yield 65%. White solid substance. M.p. 85 °C. HRMS: 419.0545 [M]^+^; calcd. 419.0549 (C_20_H_22_O_2_N_1_^79^Br_1_^32^S_1_)^+^. ^1^H-NMR (400 MHz, CDCl_3_) (ppm) δ: 1.56 (s, 3H, H-19), 1.63 (s, 3H, H-18), 1.78 (s, 3H, H-20), 1.92–2.16 (m, 4H, H-14, H-15), 4.32 (d, 2H, *J*_11,12_ = 7.5 Hz, H-11), 5.02 (t, 1H, *J*_16,15_ = 6.7 Hz, H-16), 5.21 (t, 1H, *J*_12,11_ = 6.7 Hz, H-12), 7.33 (dt, 2H, *J*_7,6_ = 8.5 Hz, *J*_7,10_ = 1.6 Hz, H-7, H-9), 7.58 (dt, 2H, *J*_6,7_ = 8.5 Hz, *J*_6,9_ = 1.6 Hz, H-6, H-10), 7.78 (s, 1H, H-4). ^13^C-NMR (100 MHz, CDCl_3_) (ppm) δ: 167.01 (s, C-1), 165.86 (s, C-2), 122.54 (s, C-3), 123.55 (d, C-4), 132.11 (s, C-5), 131.27 (d, C-6, C-10), 132.37 (d, C-7, C-9), 124.81 (s, C-8), 39.38 (t, C-11), 116.43 (d, C-12), 142.06 (s, C-13), 39.80 (t, C-14), 26.13 (t, C-15), 123.55 (d, C-16), 131.70 (s, C-17), 25.53 (k, C-18), 17.55 (k, C-19), 16.32 (k, C-20).

*(Z)-5-(4-bromobenzylidene)-3-(2-((1R,5S)-6,6-dimethylbicyclo[3.1.1]hept-2-en-2-yl)ethyl)thiazolidine-2,4-dione***21f** (Figures S49–S51).

Yield 48%. White solid substance. M.p. 131 °C. HRMS: 431.0545 [M]^+^; calcd. 431.0549 (C_21_H_22_O_2_N_1_^79^Br_1_^32^S_1_)^+^. ${\left[\alpha \right]}_{589}^{25}$ = −4.52 (c = 0.58, CDCl_3_). ^1^H-NMR (400 MHz, CDCl_3_) (ppm) δ: 0.80 (s, 3H, H-20), 1.08 (d, 1H, *J*_17_^a^_,17_^b^ = 8.6 Hz, H-19^a^), 1.26 (s, 3H, H-21), 2.01–2.11 (m, 2H, H-16, H-18), 2.15–2.22 (m, 2H, H-15), 2.25–2.50 (m, 3H, H-19^b^, H-12), 3.63–3.88 (m, 2H, H-11), 5.30 (m, 1H, H-14), 7.34 (dt, 2H, *J*_7,6_ = 8.4 Hz, *J*_7,10_ = 1.6 Hz, H-7, H-9), 7.58 (dt, 2H, *J*_6,7_ = 8.4 Hz, *J*_6,9_ = 1.6 Hz, H-6, H-10), 7.78 (s, 1H, H-4). ^13^C-NMR (100 MHz, CDCl_3_) (ppm) δ: 167.10 (s, C-1), 165.88 (s, C-2), 122.26 (s, C-3), 123.50 (d, C-4), 132.04 (s, C-5), 131.32 (d, C-6, C-10), 132.36 (d, C-7, C-9), 124.85 (s, C-8), 40.34 (t, C-11), 34.45 (t, C-12), 143.79 (s, C-13), 119.36 (d, C-14), 31.23 (t, C-15), 45.31 (d, C-16), 37.89 (s, C-17), 40.46 (d, C-18), 31.63 (t, C-19), 26.11 (k, C-20), 21.00 (k, C-21).

*(S,Z)-3-Benzyl-5-(2-(2,2,3-trimethylcyclopent-3-enyl)ethylidene)thiazolidine-2,4-dione***19g**. Yield 40%. Yellow oily substance. The spectrum data were similar to those mentioned here [37].

*(Z)-3-[(E)-3,7-Dimethylocta-2,6-dienyl]-5-[2-((S)-(2-(2,2,3-trimethylcyclopent-3-enyl)ethylidene)thiazolidine-2,4-dione***20g**. Yield 50%. Yellow oily substance. The spectrum data were similar to those mentioned here [37].

*(Z)-3-(2-(1R,5S)-6,6-dimethylbicyclo[3.1.1]hept-2-en-2-yl)ethyl)-5-(2-((S)-2,2,3-trimethylcyclopent-3-enyl)ethylidene)thiazolidine-2,4-dione* **21g** (Figures S52–S54).

Yield 40%. Yellowish oily substance. HRMS: 399.2231 [M]^+^; calcd. 399.2237 (C_24_H_33_O_2_N_1_^32^S_2_)^+^. ${\left[\alpha \right]}_{589}^{24}$ = −13.1 (c = 0.51, CDCl_3_). ^1^H-NMR (300 MHz, CDCl_3_) (ppm) δ: 0.79 (s, 3H, H-23), 0.80 (s, 3H, H-12), 0.99 (s, 3H, H-13), 1.07 (d, 1H, *J*_22_^a^_, 22_^b^ = 9.0 Hz, H-22^a^), 1.25 (s, 3H, H-24), 1.59 (m, 3H, H-11), 2.00–2.11 (m, 12H, H-5, H-6, H-7, H-15, H-18, H-19, H-21, H-22^b^), 3.55–3.78 (m, 1H, H-14), 5.20 (s, 1H, H-8), 5.29 (s, 1H, H-17), 7.07 (t, 1H, *J*_4,5_ = 7.6 Hz, H-4). ^13^C-NMR (75 MHz, CDCl_3_) (ppm) δ: 167.47 (s, C-1), 164.82 (s, C-2), 125.29 (s, C-3), 138.08 (d, C-4), 32.72 (t, C-5), 49.10 (d, C-6), 35.44 (t, C-7), 121.25 (d, C-8), 148.13 (s, C-9), 46.91 (s, C-10), 12.50 (k, C-11), 19.73 (k, C-12), 25.73 (k, C-13), 40.00 (t, C-14), 34.45 (t, C-15), 143.95 (s, C-16), 119.19 (d, C-17), 31.25 (t, C-18), 49.10 (d, C-19), 37.89 (s, C-20), 45.35 (d, C-21), 31.63 (t, C-22), 26.12 (k, C-23), 21.01 (k, C-24).

### 3.2. Biology Section

#### 3.2.1. Real-Time Detection of TDP1 Activity

The biosensor (5′-[FAM] AAC GTC AGGGTC TTC C [BHQ]-3′) was synthesized in the Laboratory of Nucleic Acid Chemistry at the Institute of Chemical Biology and Fundamental Medicine (Novosibirsk, Russia) and was used for TDP1 enzyme activity real-time fluorescence detection [39]. The reaction mixture (200 µL) contained a TDP1 reaction buffer (50 mM Tris-HCl, pH 8.0, 50 mM NaCl, and 7 mM β-mercaptoethanol), 50 nM oligonucleotide, varied concentrations of the tested compounds, and purified TDP1 in a final concentration of 1.5 nM. The recombinant TDP1 was purified to homogeneity by chromatography on Ni-chelating resin and phosphocellulose P11, as described [40], using plasmid pET 16B-TDP1, kindly provided by Dr. K.W. Caldecott (University of Sussex, United Kingdom).

The reaction mixtures were incubated at a constant temperature of 26 °C in a POLARstar OPTIMA fluorimeter (BMG LABTECH, GmbH, Ortenberg, Germany). Fluorescence intensity was measured (Ex485/Em520 nm) every 1 min for 10 min. The average values of the half maximal inhibitory concentration (IC_50_) were determined using a six-point concentration response curve and calculated using MARS Data Analysis 2.0 (BMG LABTECH). The 50% inhibitory concentration (IC_50_) was defined as the concentration of the compound that inhibited 50% of the enzyme activity, when compared to the untreated controls. At least three independent experiments were carried out to obtain the IC_50_ values.

#### 3.2.2. Cytotoxicity Assays

Human colon adenocarcinoma HCT-116 and human lung fibroblast MRC-5 cell lines were provided by the Cell Culture Bank of the State Research Center for Virology and Biotechnology Vector, Novosibirsk, Russia. Cells were cultured in DMEM medium (Invitrogen) supplemented with 10% fetal bovine serum (FBS) (Invitrogen), penicillin (100 units/mL), and streptomycin (100 µg/mL) at 37 °C and 5% CO_2_ in a humid atmosphere. After the formation of a 30–50% monolayer, the tested compounds were added to the medium. Because the compounds are soluble in DMSO, we minimized the DMSO content in the culture medium. The volume of the added reagents was 1/100 of the total volume of the culture medium, and the amount of DMSO was 1% of the final volume. Control cells were grown in the presence of 1% DMSO.

Cells in the exponential growth phase were seeded in 96-well plates (5000 cells per well). The cells were allowed to attach for 24 h and were treated with compounds with concentrations ranging from 1 to 100 µM for 72 h at 37 °C. The cytotoxicity of the compounds was examined using the EZ4U Cell Proliferation and Cytotoxicity Assay (Biomedica, Austria), according to the manufacturer’s protocols. All concentrations were performed in triplicate. All measurements were repeated three times.

To study the effect of the compounds on the cytotoxicity of topotecan, an aqueous solution of topotecan was used at concentrations from 1 to 10 μM against the background of 10 μM of the compounds. Cells treated with compounds alone without topotecan served as controls.

### 3.3. Modeling Section

The compounds were docked against the crystal structure of TDP1 (PDB ID: 6W7K, resolution 1.70 Å) [22], which were obtained from the Protein Data Bank (PDB) [41,42]. The GOLD (v2020.2.0) software suite was used to prepare the crystal structure for docking, i.e., the hydrogen atoms were added, water molecules deleted and the co-crystallized ligand was identified: 4-[(2-phenylimidazo[1,2-*a*]pyridin-3-yl)amino]benzene-1,2-dicarboxylic acid (TG7). The Scigress version FJ 2.6 program [43] software suite was used to build the ligands and the MM3 [44,45,46] force field was applied to identify the global minimum value using the CONFLEX method [47], followed by structural optimization. The docking center for the TDP1 catalytic pocket was defined as the position of the co-crystallized ligand TG7 with a 10 Å radius. Fifty docking runs were allowed for each ligand with default search efficiency (100%). The basic amino acids lysine and arginine were defined as protonated. Furthermore, aspartic and glutamic acids were assumed to be deprotonated. The GoldScore (GS) [24] and ChemScore (CS) [25,26] ChemPLP (Piecewise Linear Potential) [7,27] and ASP (Astex Statistical Potential) [28] scoring functions were implemented to predict the binding modes and relative binding energies of the ligands using the GOLD v2020.2.0 software suite.

The QikProp 6.2 [48] software package was used to calculate the molecular descriptors of the molecules. The reliability of QikProp for the calculated descriptors was established [49]. The Known Drug Indexes (KDI) were calculated from the molecular descriptors, as described by Eurtivong and Reynisson [33]. For application in Excel, columns for each property were created and the following equations were used to derive the KDI numbers for each descriptor: KDI MW: = EXP(-((*MW*-371.76)^2)/(2*(112.76^2))), KDI Log P: = EXP(-((*LogP*-2.82)^2)/(2*(2.21^2))), KDI HD: = EXP(-((*HD-*1.88)^2)/(2*(1.7^2))), KDI HA: = EXP(-((*HA*-5.72)^2)/(2*(2.86^2))), KDI RB = EXP(-((*RB*-4.44)^2)/(2*(3.55^2))), and KDI PSA: = EXP(-((*PSA*-79.4)^2)/(2*(54.16^2))). These equations could simply be copied into Excel and the descriptor name (e.g., *MW*) was substituted with the value in the relevant column. To obtain KDI_2A_ values, the following equation was used: = (KDI MW + KDI Log P + KDI HD + KDI HA + KDI RB + KDI PSA) and for KDI_2B_: = (KDI MW × KDI Log P × KDI HD × KDI HA × KDI RB × KDI PSA).

## 4. Conclusions

In conclusion, we have synthesized and tested a series of mono- and disubstituted thiazolidinediones for the presence of inhibitory properties against TDP1. It has been shown that the substances that exhibit inhibitory activity in the low micromolar concentration range mostly belong to the series of disubstituted thiazolidinediones. The most active compounds, geranyl and (-)-nopyl, the 3-substituted 5-((4-bromothiophen-2-yl)methylene)-thiazolidine-2,4-diones, inhibit TDP1 at submicromolar concentrations. It has been found that the presence of terpene substituents in both position 3 and position 5 generally enhance activity. All of the studied compounds are non-toxic at concentrations up to 100 μM on HCT-116 (tumor) and MRC-5 (non-tumor) cells, but do not have a sensitizing effect when combined with the antitumor drug topotecan. Since we previously observed the desired effect for a series of disubstituted thiazolidine-4-ones, it is possible that the thiazolidinedione fragment contributes to the absence of sensitization, due to its multi-targeting effect. This study contributed to the elucidation of SAR and revealed pharmacophore fragments and undesirable structural motifs for binding to TDP1 and the development of a direction in medicinal chemistry dedicated to the creation of inhibitors of this DNA repair enzyme.

## Figures and Tables

**Table 1:** TDP1 inhibitory activities of the thiazolidine-2,4-dione derivatives (IC_50_, μM).

Compounds		18	19	20	21
		H	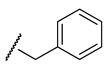	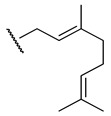	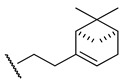
**a**	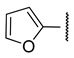	>100	>100	3.4 ± 0.1	>100
**b**	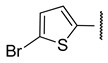	1.1 ± 0.2	1.9 ± 0.3	>100	2.5 ± 0.4
**c**	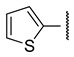	>100	>100	>100	>100
**d**	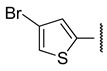	>100	>100	0.65 ± 0.07	0.55 ± 0.07
**e**	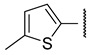	>100	>100	2.9 ± 0.1	2.3 ± 0.1
**f**	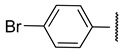	>100	1.6 ± 0.3	>100	>100
**g**	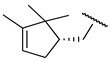	>100	2.4 ± 1.1	4.1 ± 0.1	2.4 ± 0.1

Furamidine: 1.2 ± 0.3 μM.

## Data Availability

The data presented in this study are available upon request from the corresponding author.

## Supplementary Materials

The following supporting information can be downloaded at: https://www.mdpi.com/article/10.3390/ijms24043834/s1.
