# Supplementary material for: Novel TDP1 Inhibitors: Disubstituted Thiazolidine-2,4-Diones Containing Monoterpene Moieties
Source: Int J Mol Sci. 2023 Feb 14;24(4):3834. doi: 10.3390/ijms24043834 (PMC9964680; doi:10.3390/ijms24043834)
Supplement: Supplementary file 1 [file ijms-24-03834-s001.zip › ijms-2156942-supplementary.pdf]

Supplementary Materials

Novel TDP1 Inhibitors: Disubstituted Thiazolidine-2,4-diones Containing Monoterpene Moieties

Dmitry I. Ivankin [1], Tatyana E. Kornienko [2], Marina A. Mikhailova [3], Nadezhda S. Dyrkheeva [2], Alexandra L. Zakharenko [2], Chigozie Achara [4], Jóhannes Reynisson [4], Victor M. Golyshev [2], Olga A. Luzina [1], Konstantin P. Volcho [1,*], Nariman F. Salakhutdinov [1,3] and Olga I. Lavrik [2,3]

1 N.N. Vorozhtsov Novosibirsk Institute of Organic Chemistry, Siberian Branch of the Russian Academy of Science, 9, Akademika Lavrentieva Ave., 630090 Novosibirsk, Russia
2 Institute of Chemical Biology and Fundamental Medicine, Siberian Branch of the Russian Academy of Science, 8, Akademika Lavrentieva Ave., 630090 Novosibirsk, Russia
3 Department of Natural Sciences, Novosibirsk State University, 630090 Novosibirsk, Russia
4 School of Pharmacy and Bioengineering, Keele University, Hornbeam Building, Newcastle-under-Lyme, Staffordshire ST5 5BC, UK
* Correspondence: volcho@nioch.nsc.ru

Table of contents

^{1}H, ^{13}C NMR, and DFS spectra of compounds	**Figures S1-S54**
Circular dichroism spectroscopy experiments	**Figure S55**
UV-vis absorbtion spectra of compounds	**Figure S56-S62**
The binding affinities as predicted by the scoring functions used to the catalytic TDP1 binding pocket and their measured IC_{50} values.	**Table S1**
The correlation plot of measured IC_{50} values against their CS counterparts.	**Figure S63**
The molecular descriptors and their corresponding Known Drug Indexes 2a and 2b ($KDI_{2a/2b}$).	**Table S2**
Definition of lead-like, drug-like and Known Drug Space (KDS) in terms of molecular descriptors. The values given are the maxima for each descriptor for the volumes of chemical space used.	**Table S3**

Chemistry

Compound **18e**.

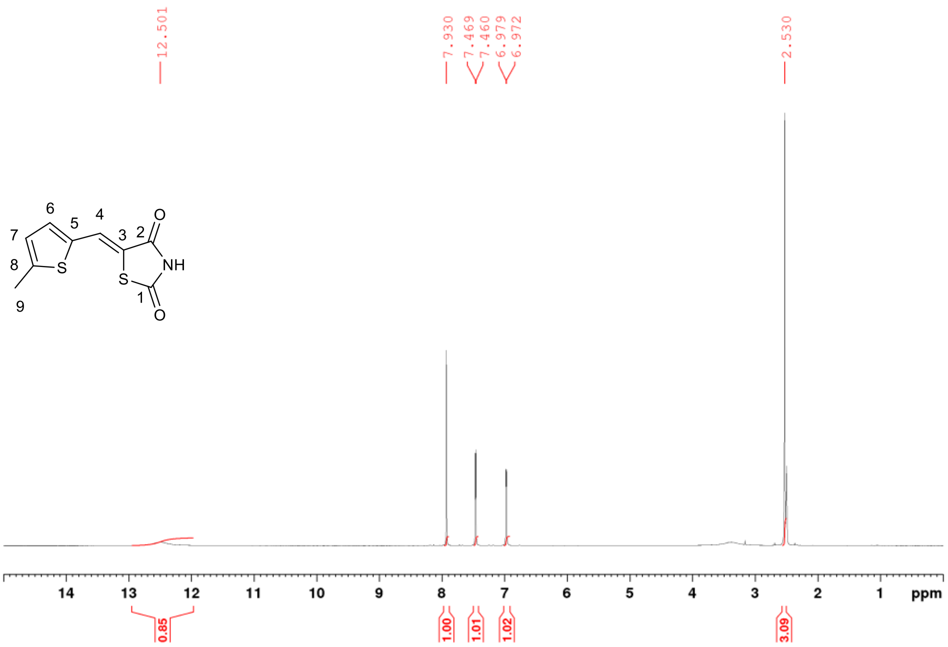

Figure S1. The ^{1}H NMR spectrum of **18e.**

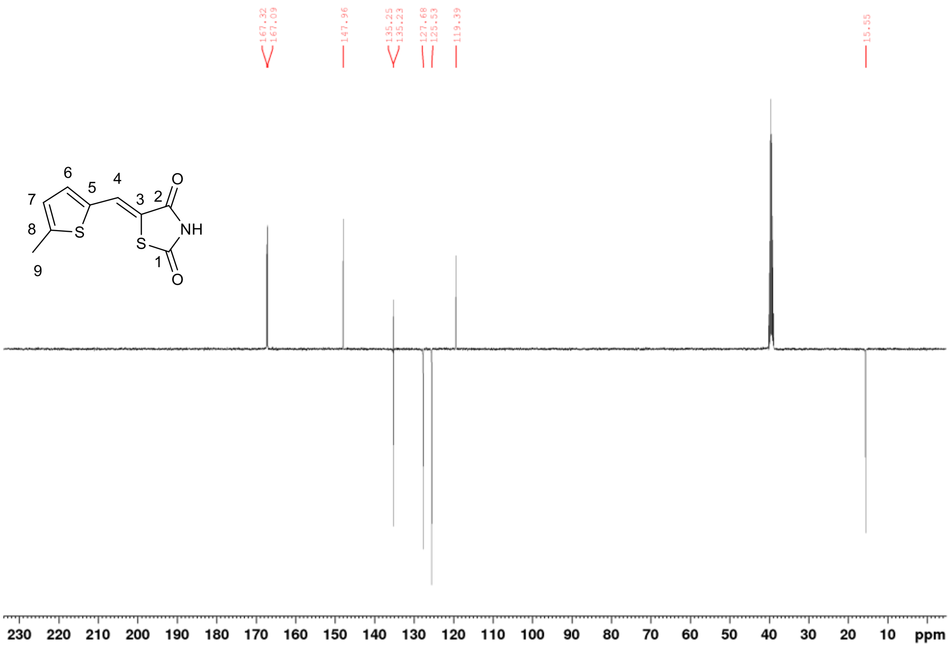

Figure S2. The ^{13}C NMR spectrum of **18e.**

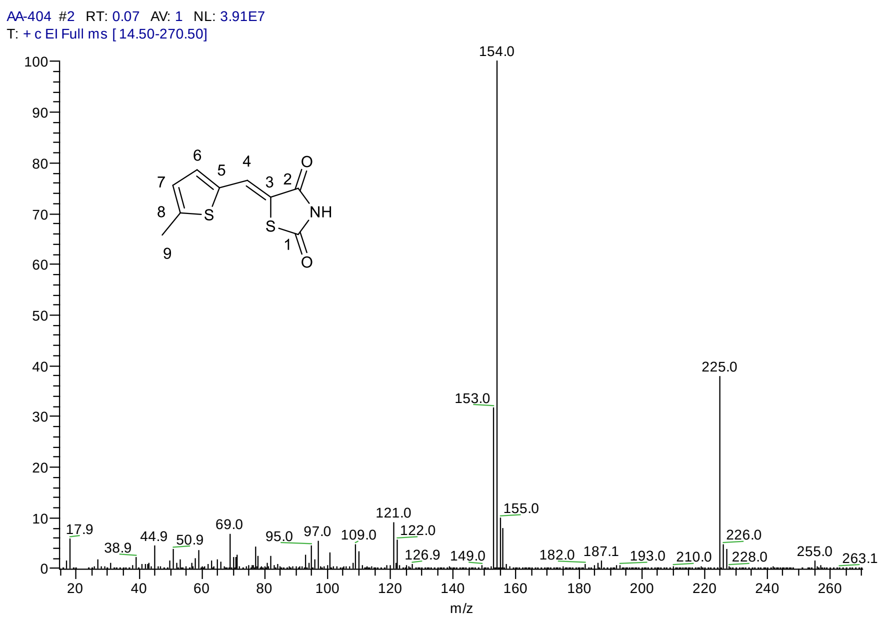

Figure S3. The DFS spectrum of **18e.**

Compound **21a**.

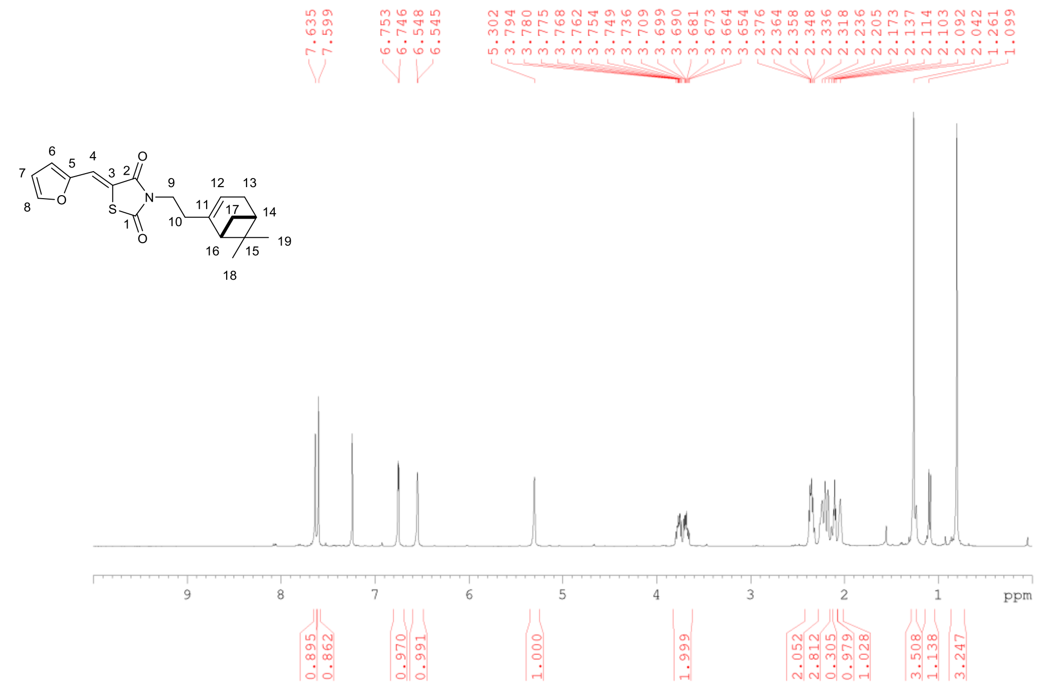

Figure S4. The ¹H NMR spectrum of **21a.**

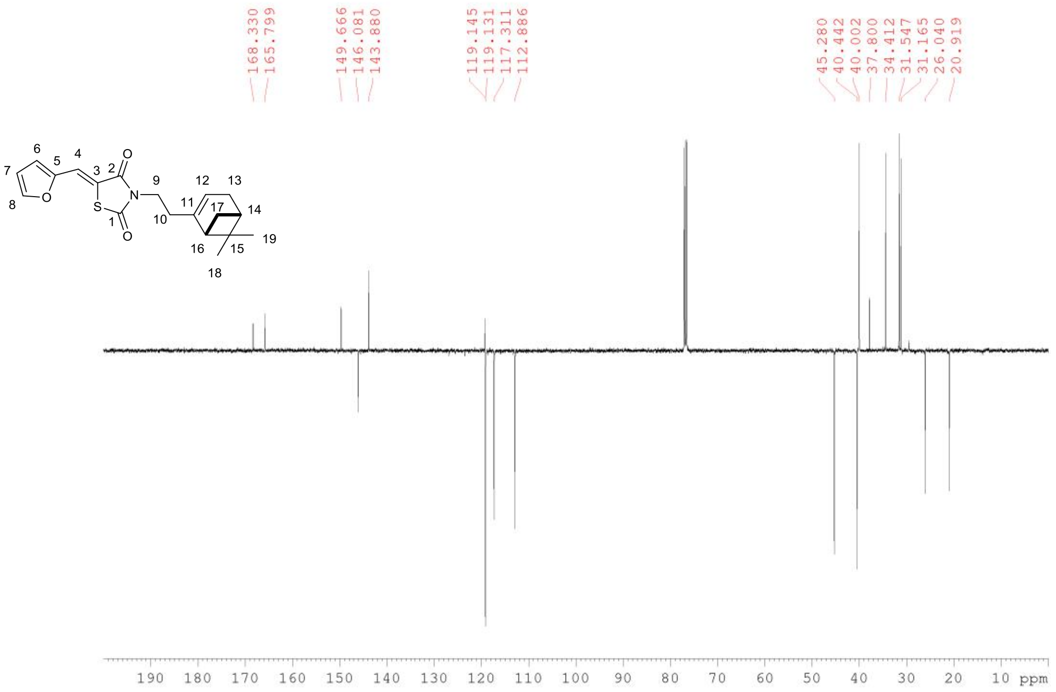

Figure S5. The ^{13}C NMR spectrum of **21a.**

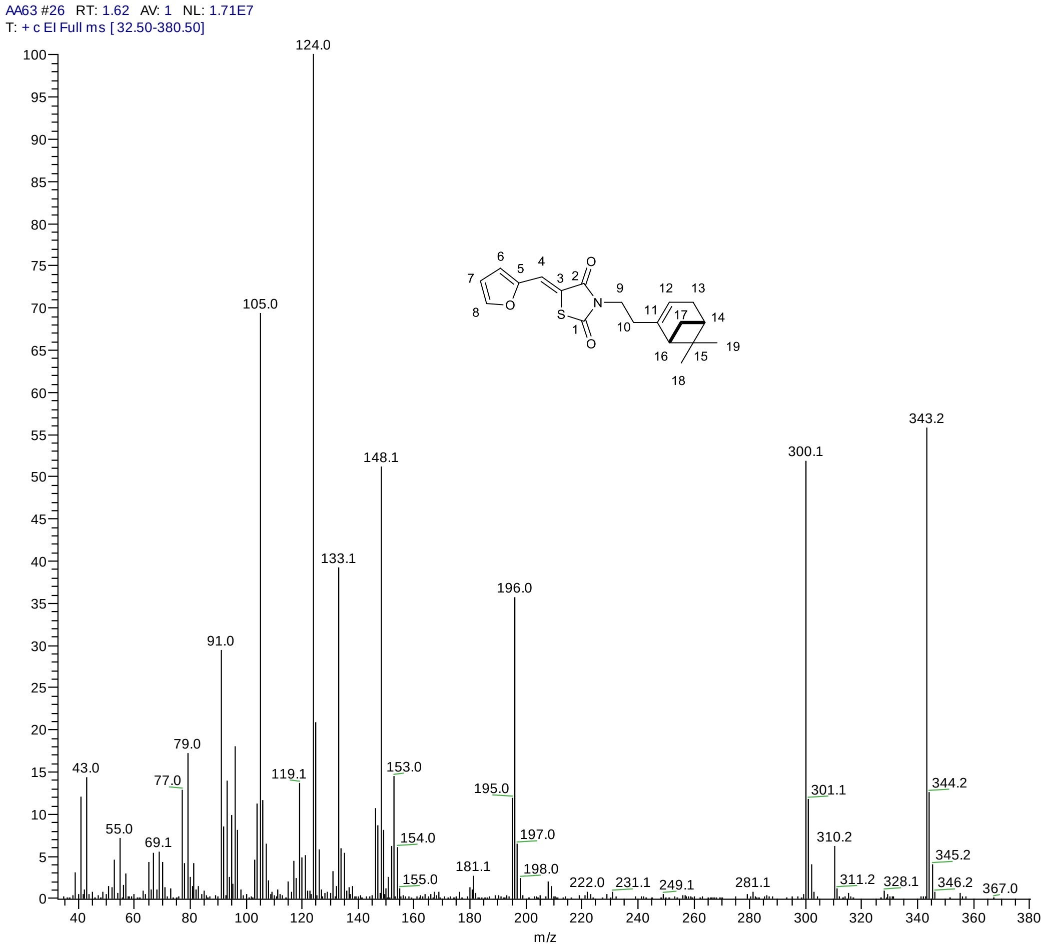

Figure S6. The DFS spectrum of **21a.**

Compound **19b**.

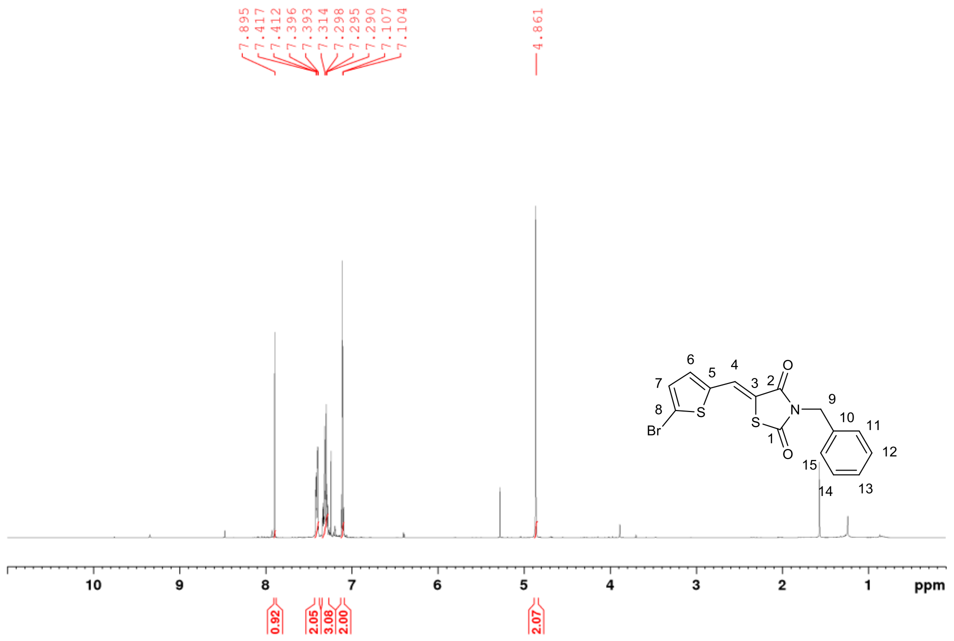

Figure S7. The ¹H NMR spectrum of **19b.**

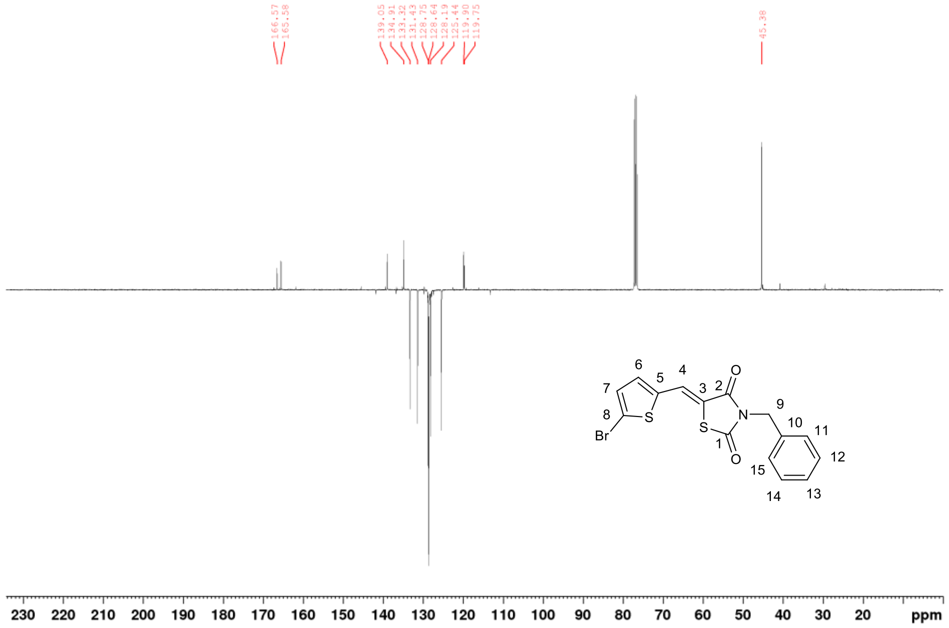

Figure S8. The ¹³C NMR spectrum of **19b.**

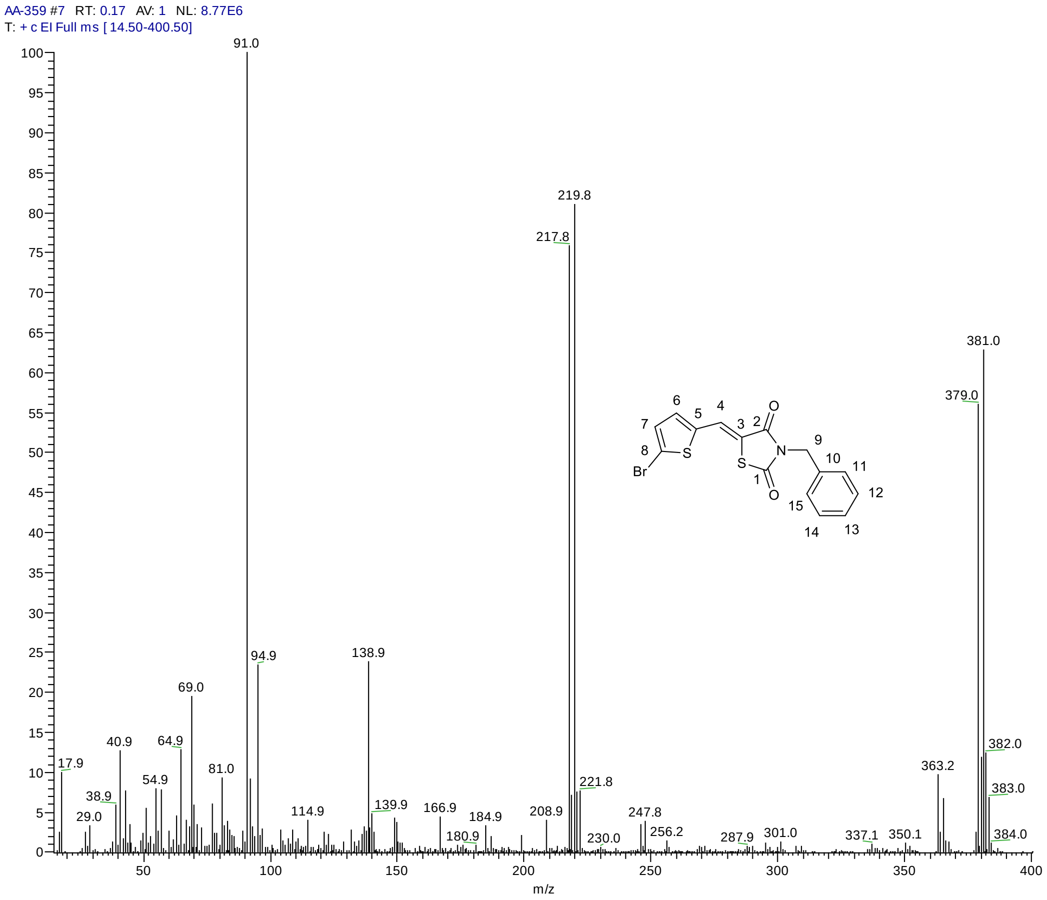

Figure S9. The DFS spectrum of **19b.**

Compound **20b**.

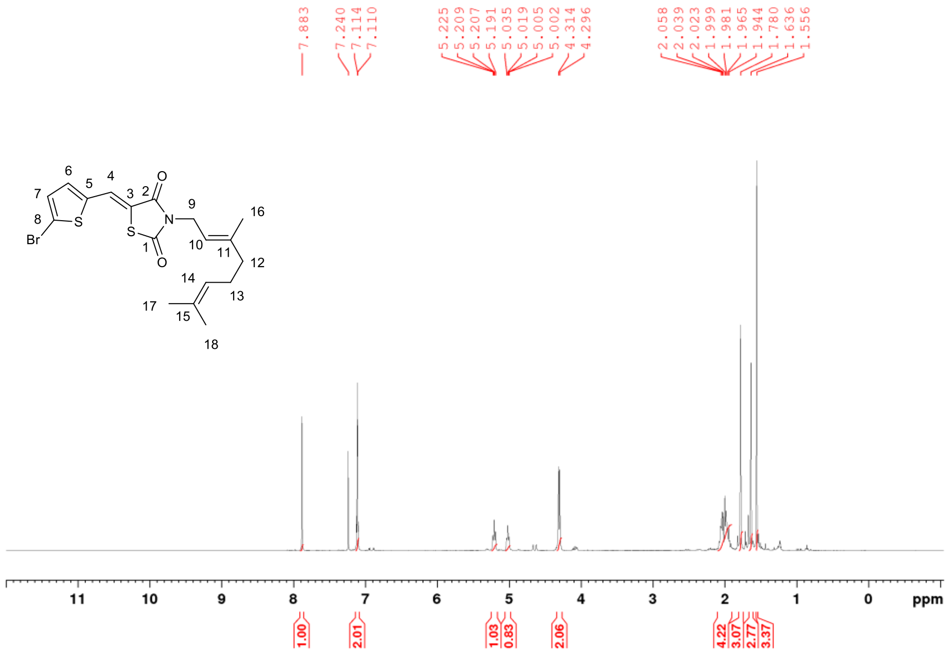

Figure S10. The ^{1}H NMR spectrum of **20b.**

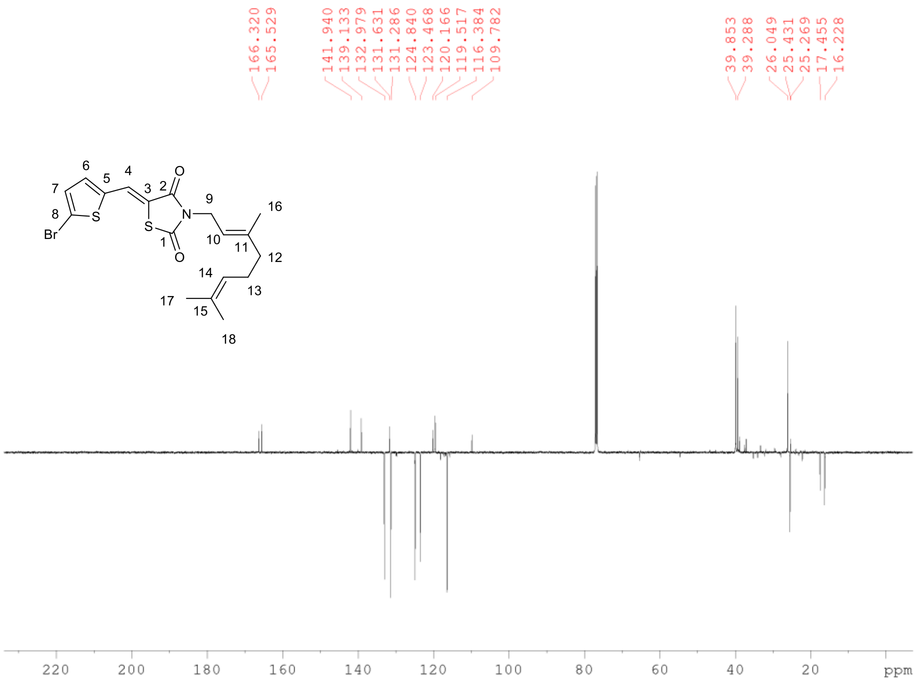

Figure S11. The ^{13}C NMR spectrum of **20b.**

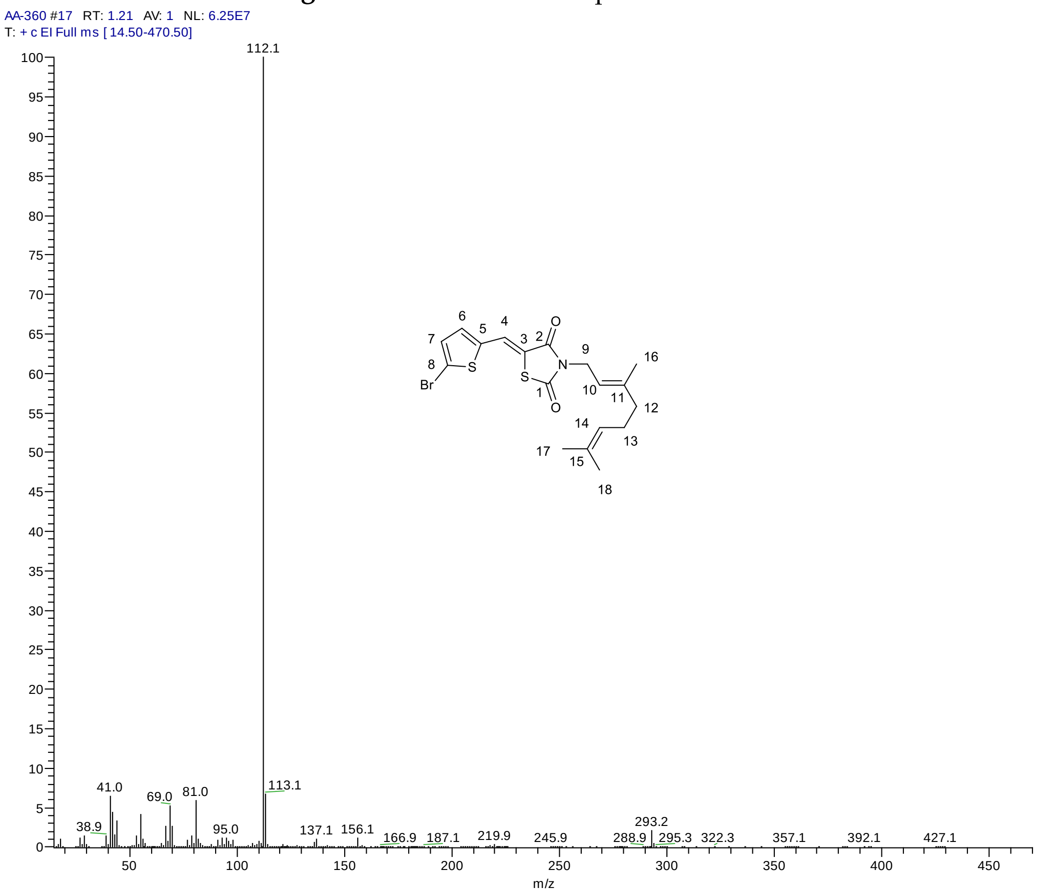

Figure S12. The DFS spectrum of **20b.**

Compound **21b**.

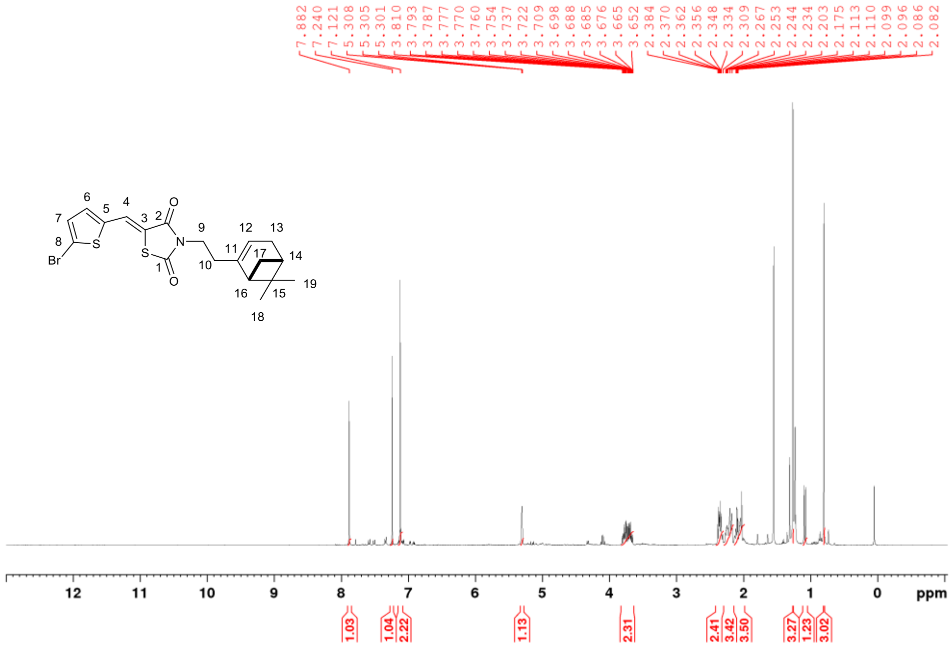

Figure S13. The ^{1}H NMR spectrum of **21b.**

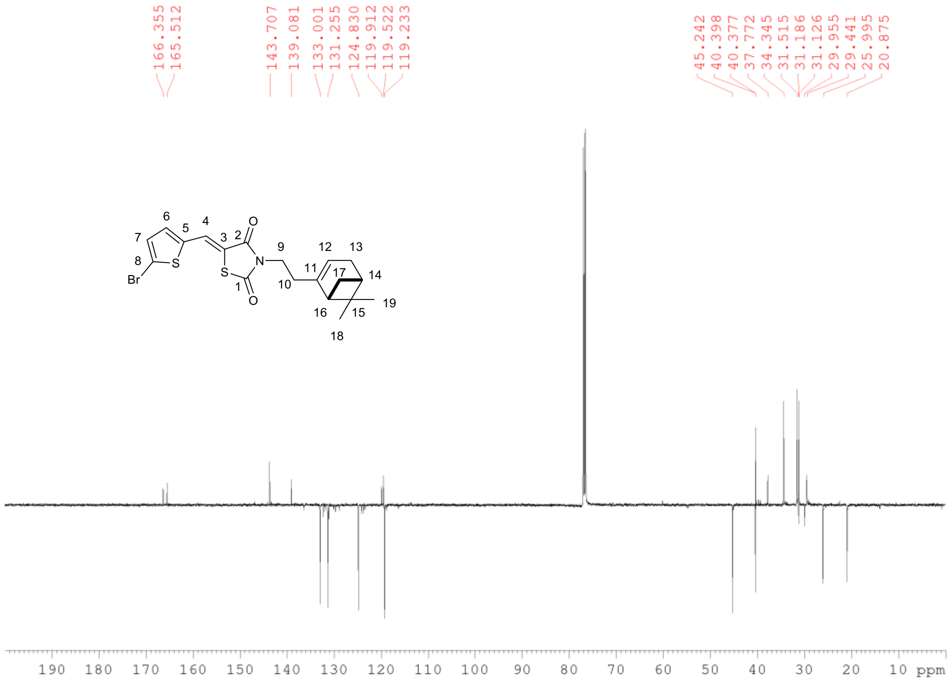

Figure S14. The ^{13}C NMR spectrum of **21b.**

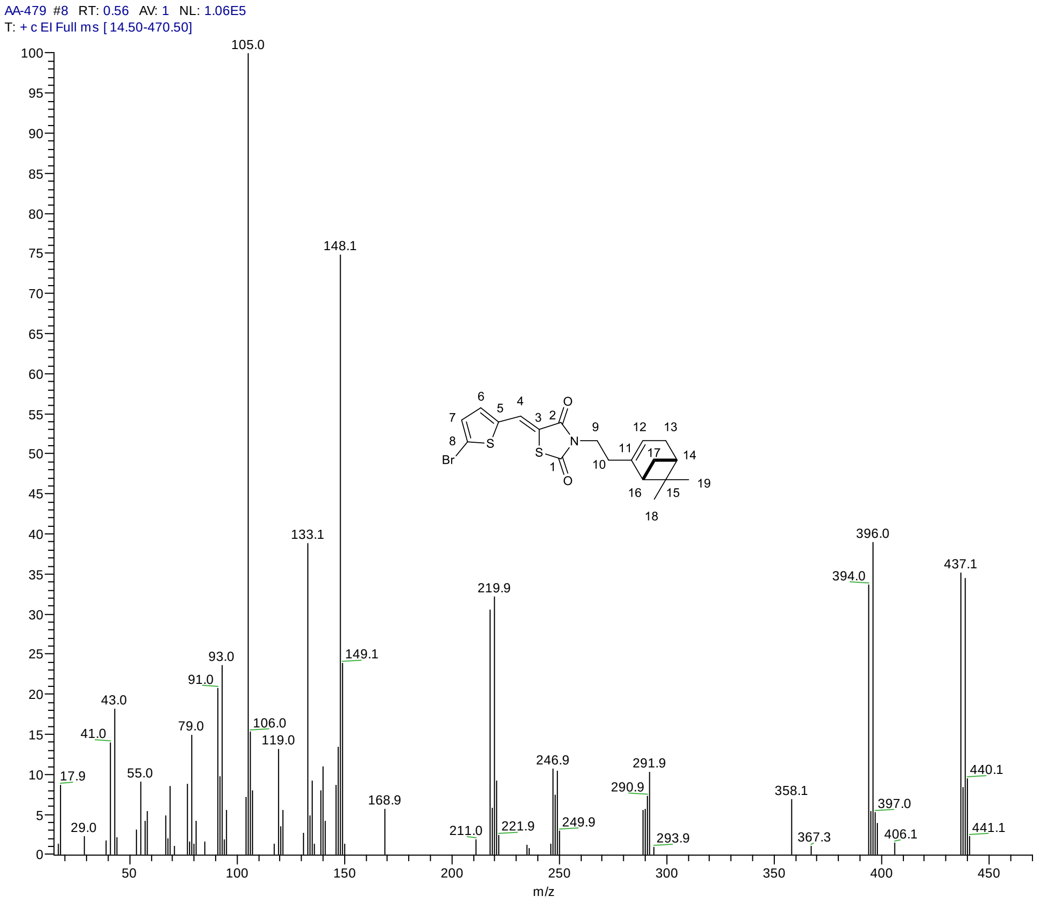

Figure S15. The DFS spectrum of **21b.**

Compound **19c**.

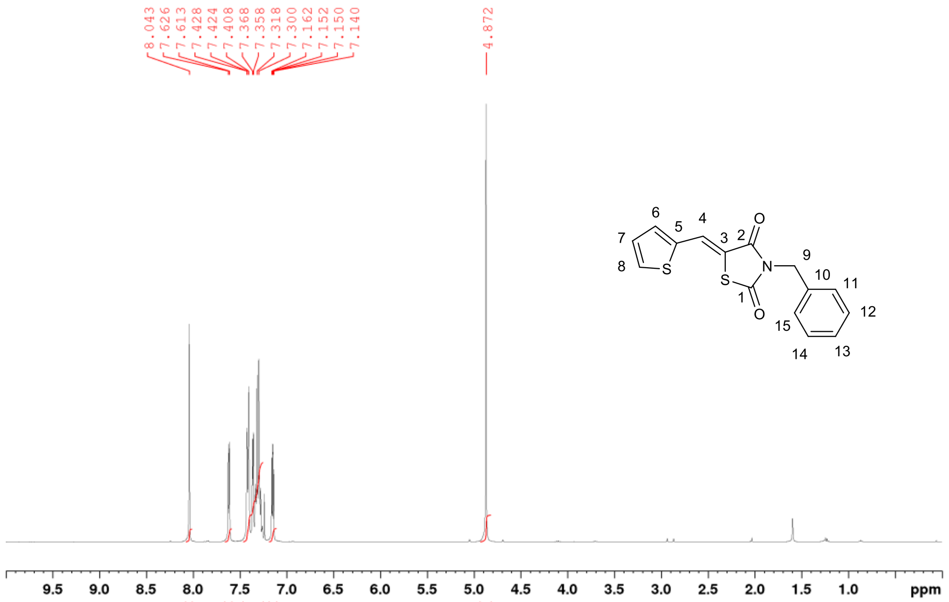

Figure S16. The ^{1}H NMR spectrum of **19c.**

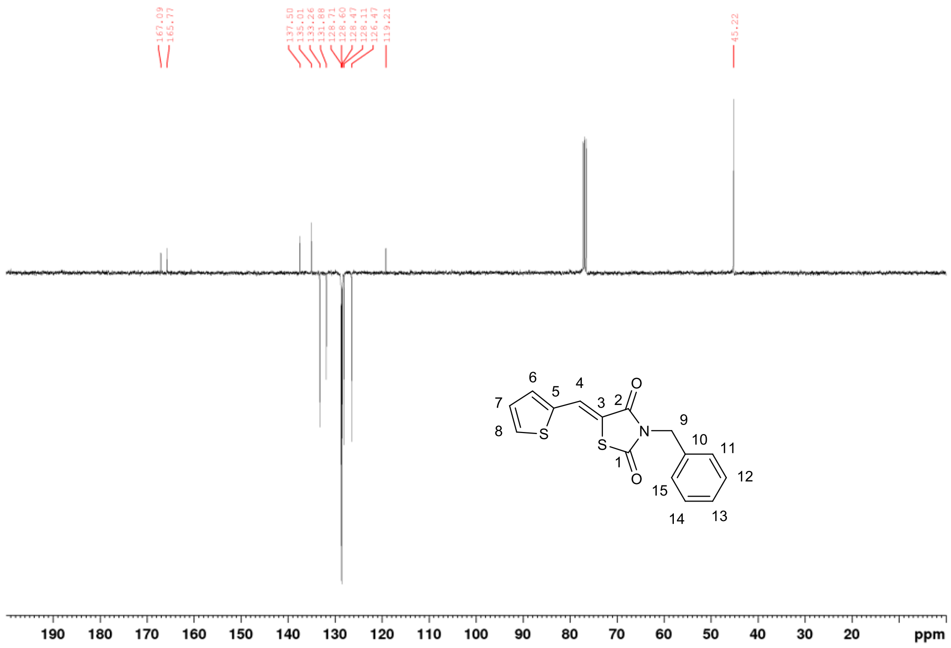

Figure S17. The ^{13}C NMR spectrum of **19c.**

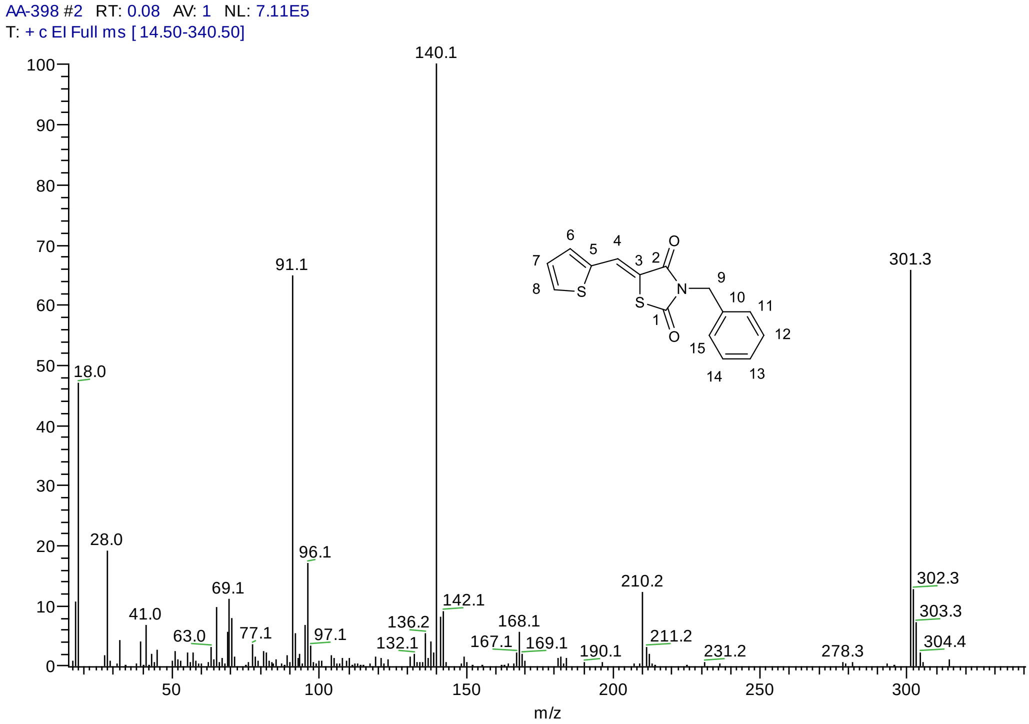

Figure S18. The DFS spectrum of **19c.**

Compound **20c**.

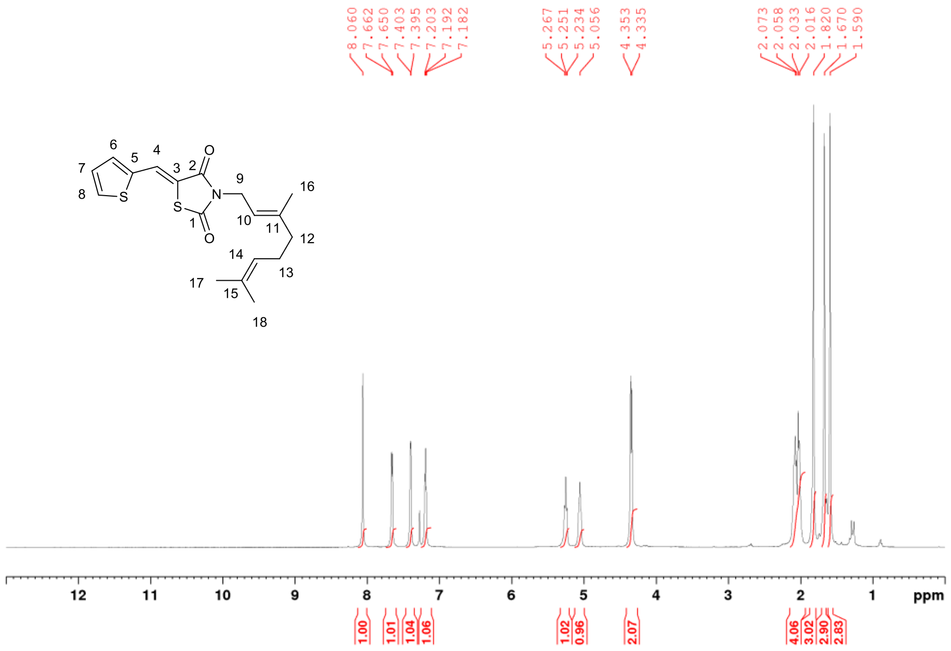

Figure S19. The ^{1}H NMR spectrum of **20c.**

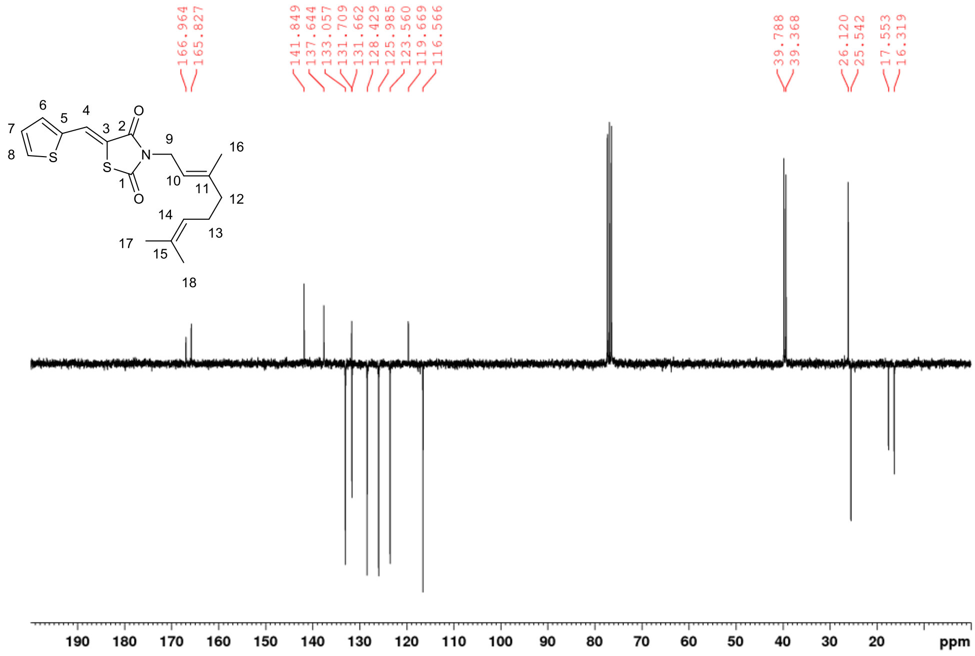

Figure S20. The ^{13}C NMR spectrum of **20c.**

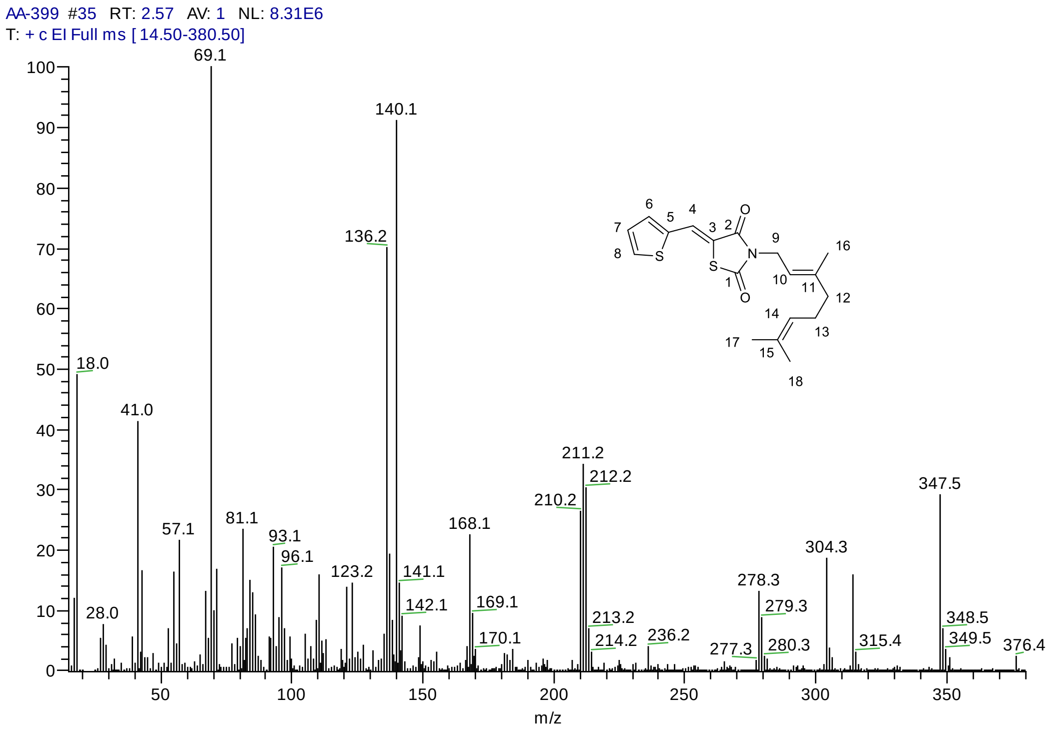

Figure S21. The DFS spectrum of **20c.**

Compound **21c**.

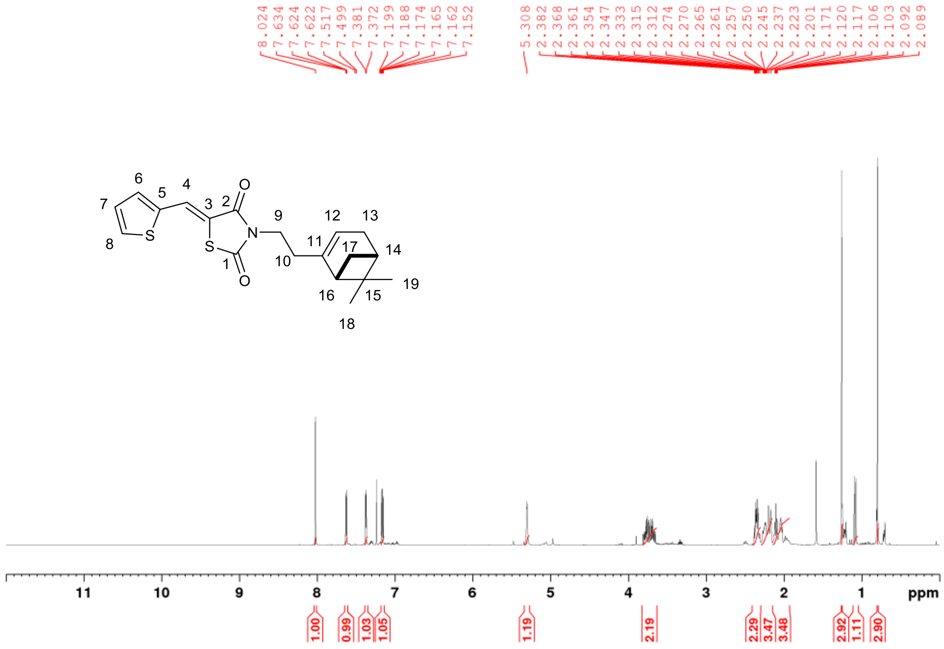

Figure S22. The ^{1}H NMR spectrum of **21c.**

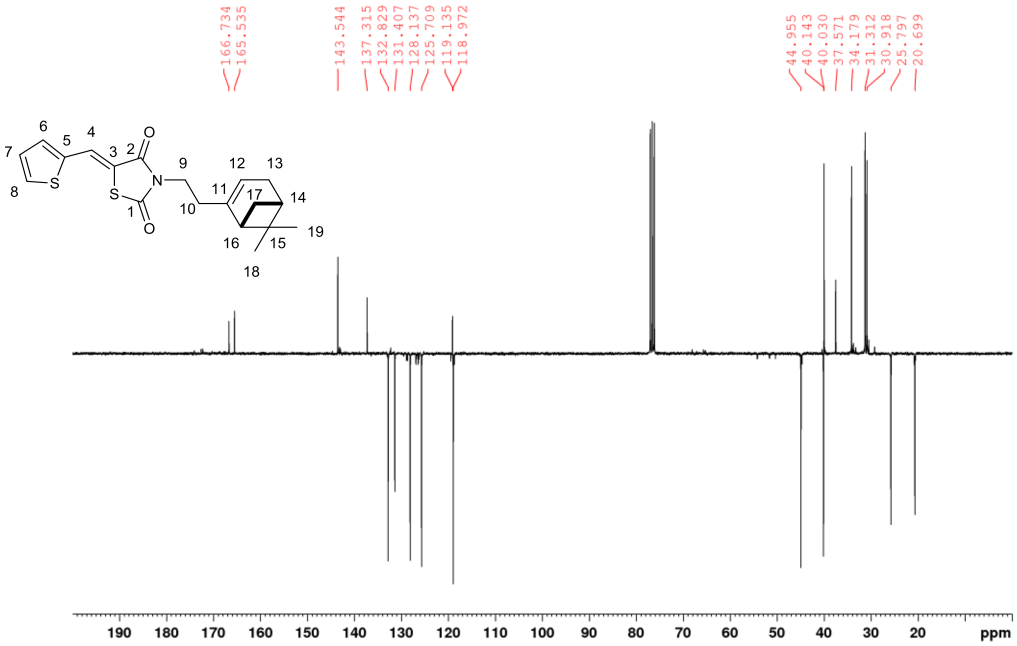

Figure S23. The ^{13}C NMR spectrum of **21c.**

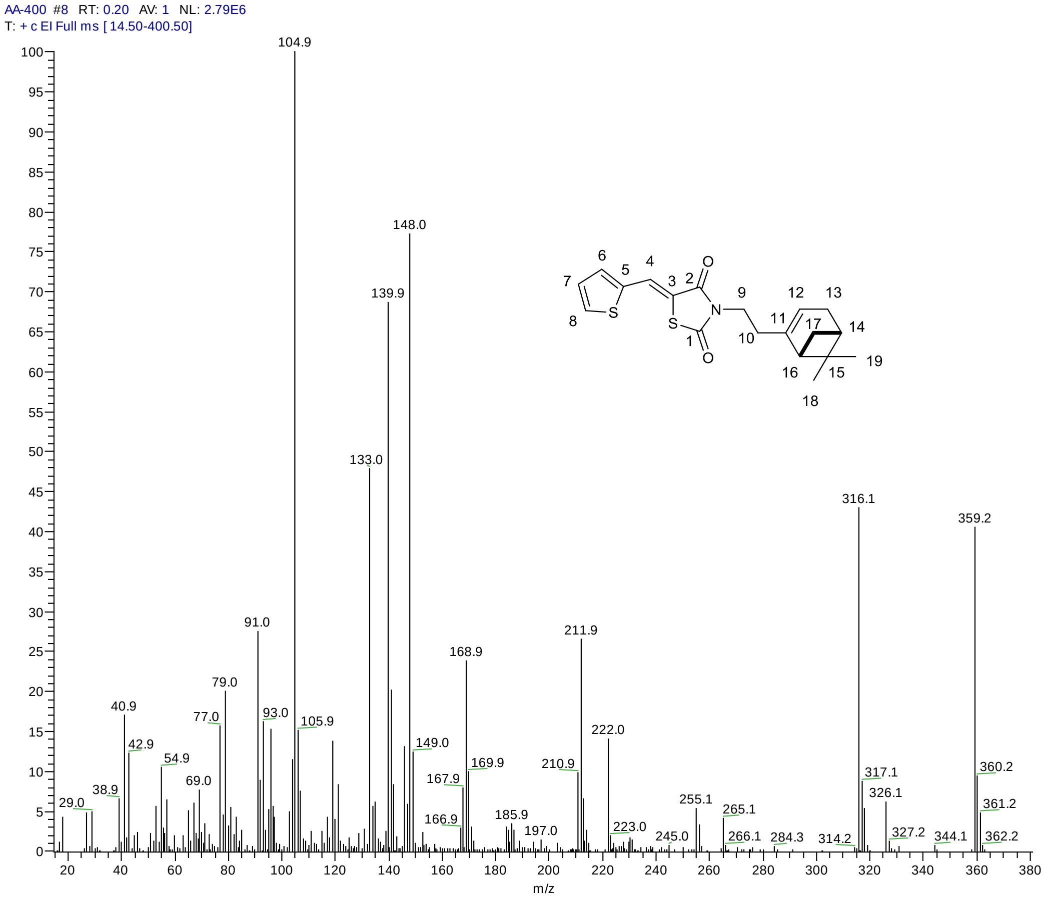

Figure S24. The DFS spectrum of **21c.**

Compound **19d**.

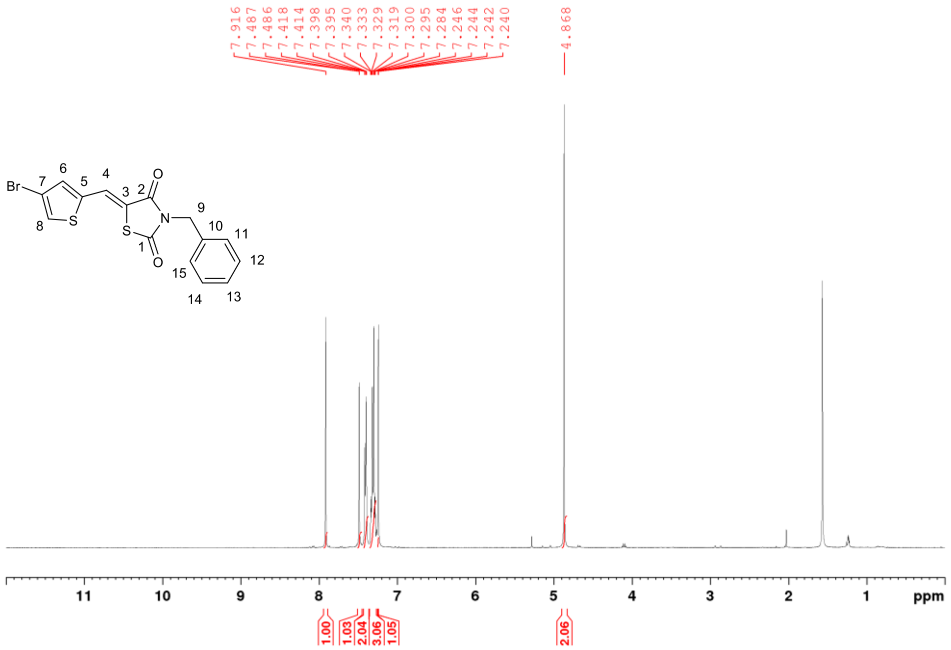

Figure S25. The ^{1}H NMR spectrum of **19d.**

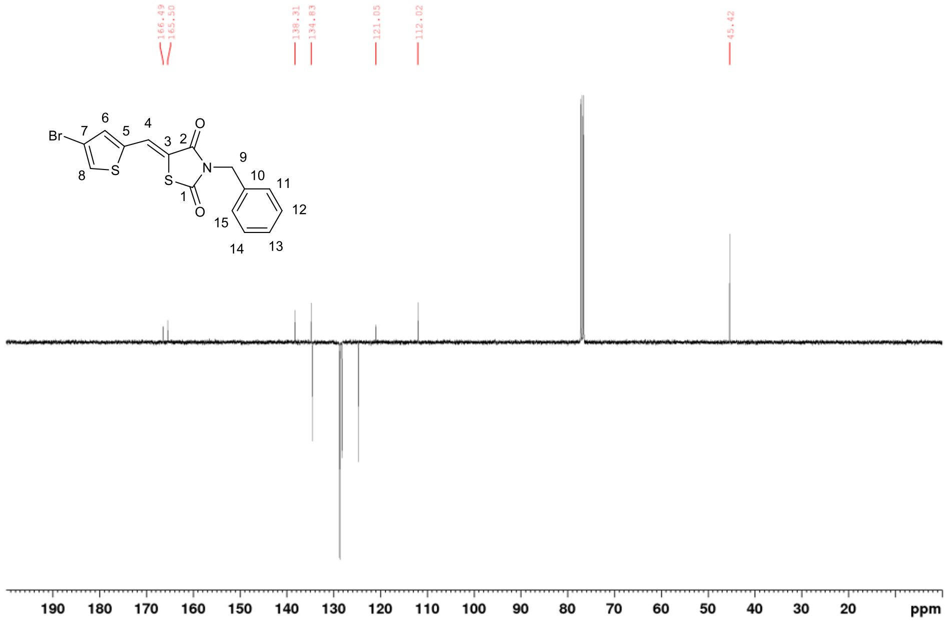

Figure S26. The ^{13}C NMR spectrum of **19d.**

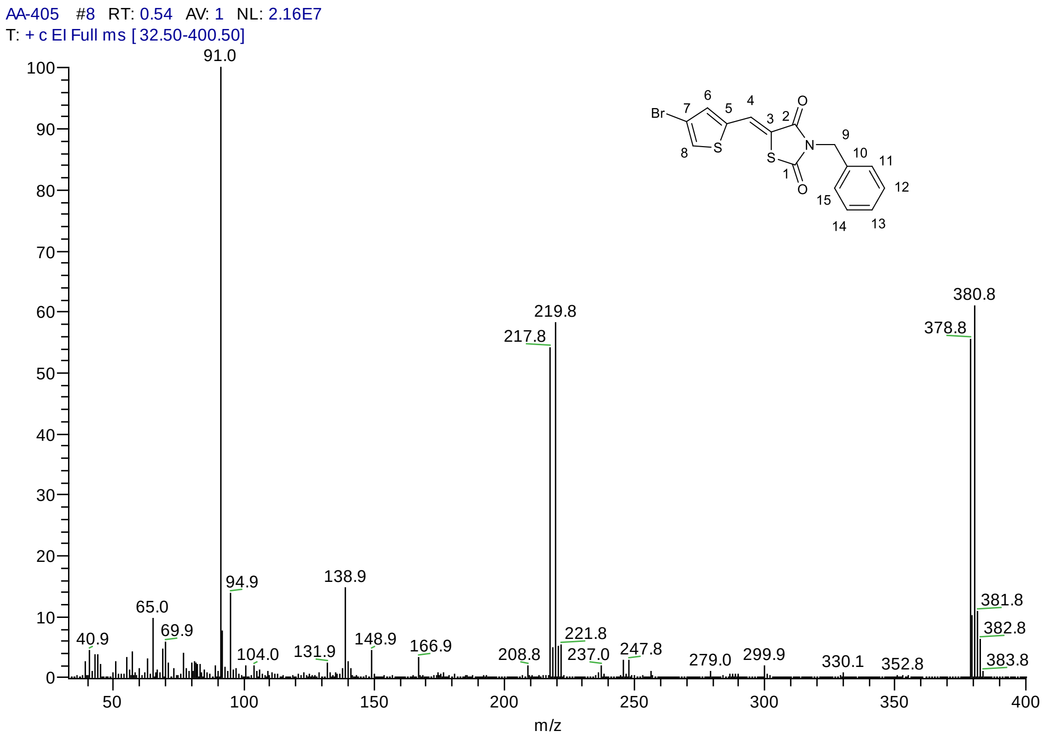

Figure S27. The DFS spectrum of **19d.**

Compound **20d**.

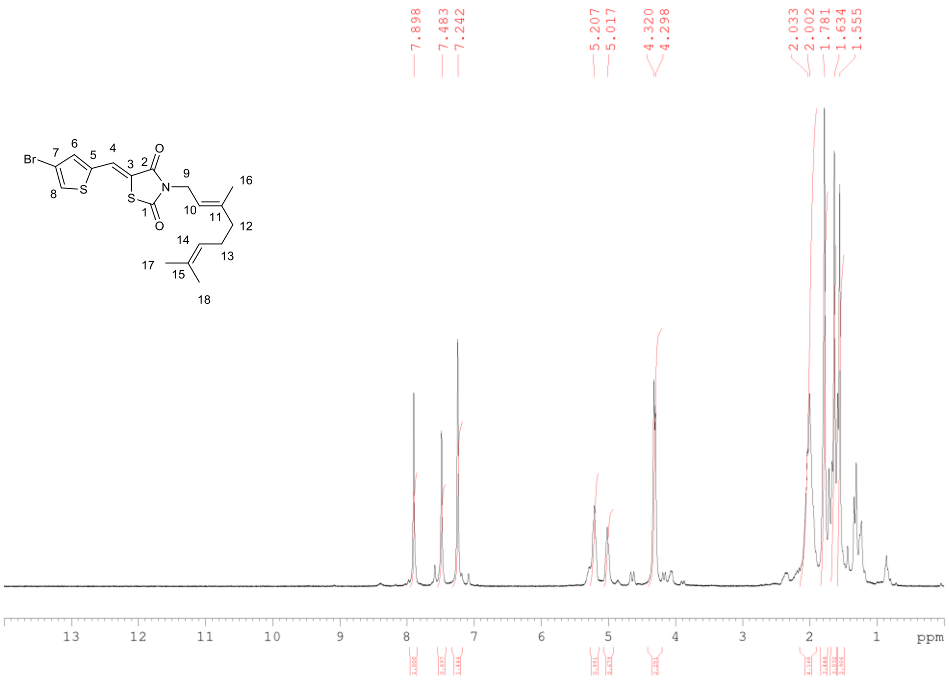

Figure S28. The ^{1}H NMR spectrum of **20d.**

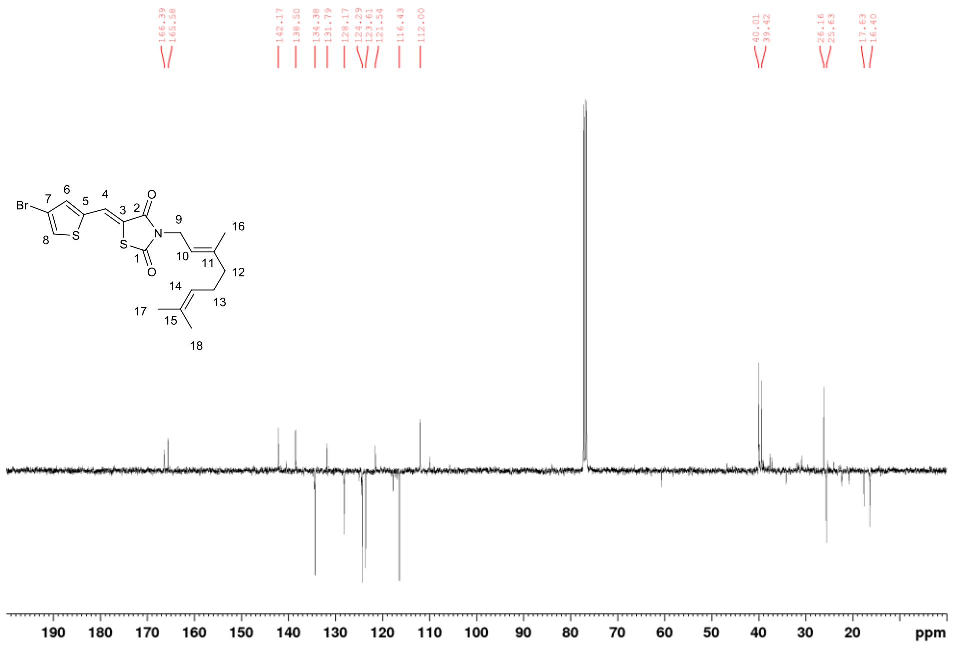

Figure S29. The ^{13}C NMR spectrum of **20d.**

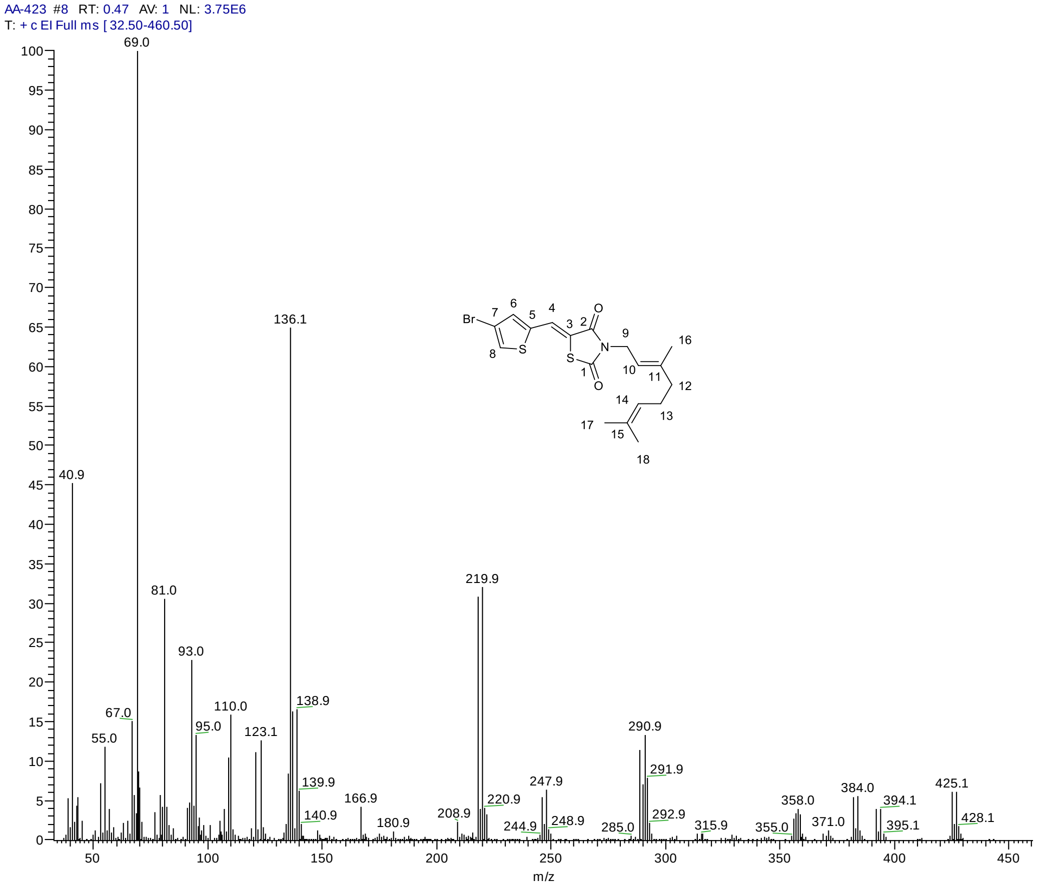

Figure S30. The DFS spectrum of **20d.**

Compound **21d**.

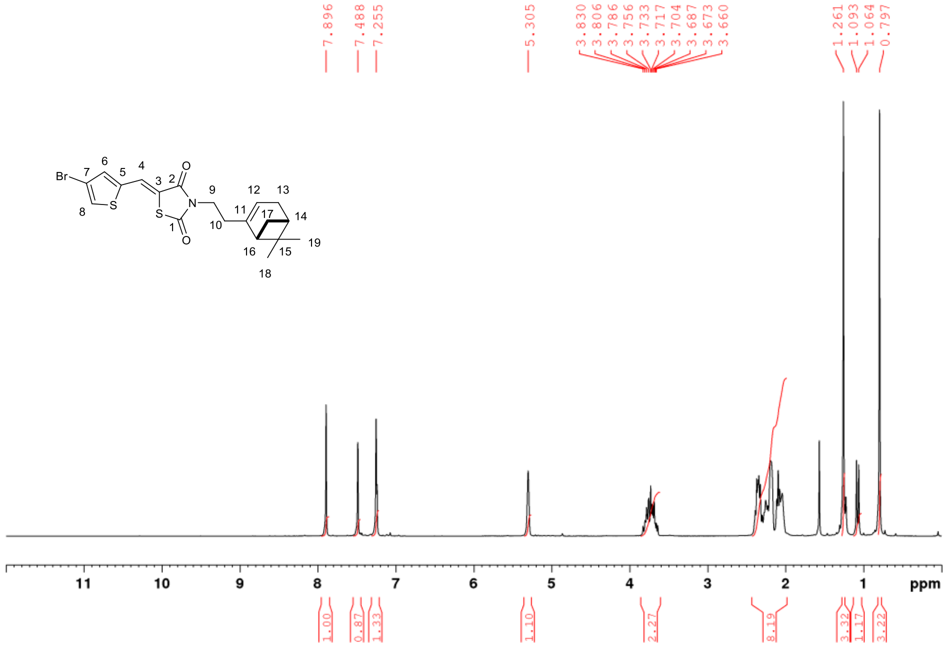

Figure S31. The ^{1}H NMR spectrum of **21d.**

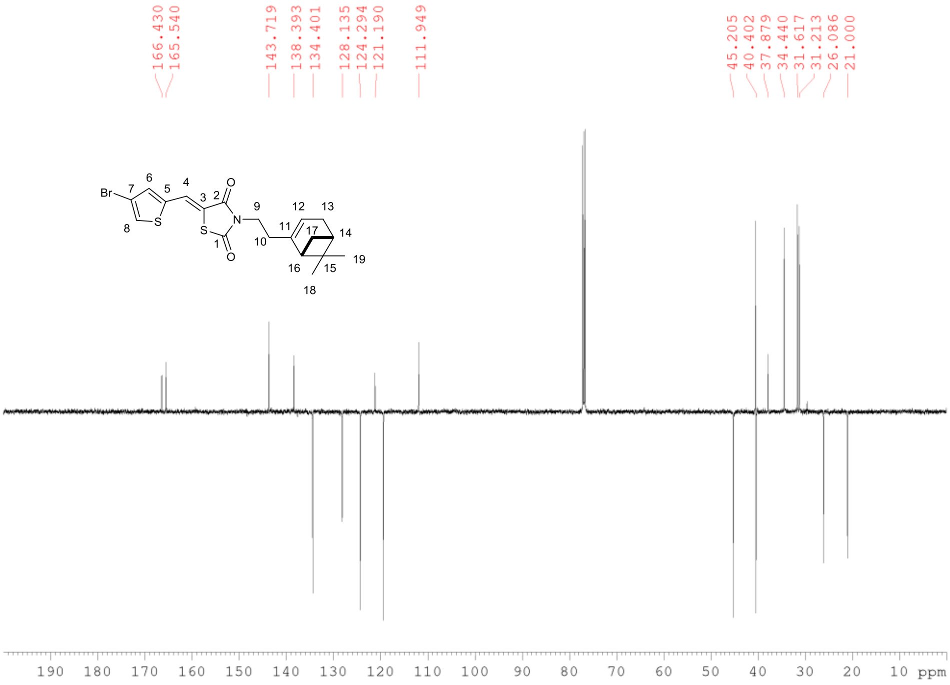

Figure S32. The ^{13}C NMR spectrum of **21d.**

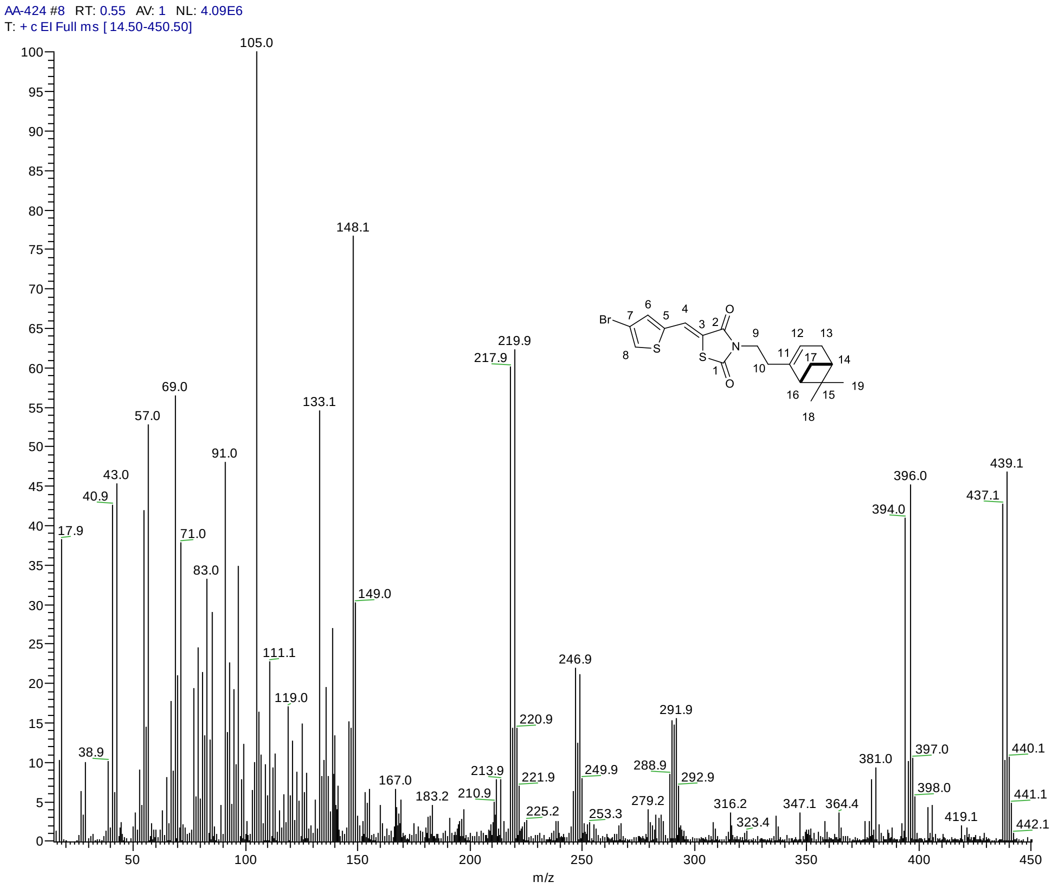

Figure S33. The DFS spectrum of **21d.**

Compound **19e**.

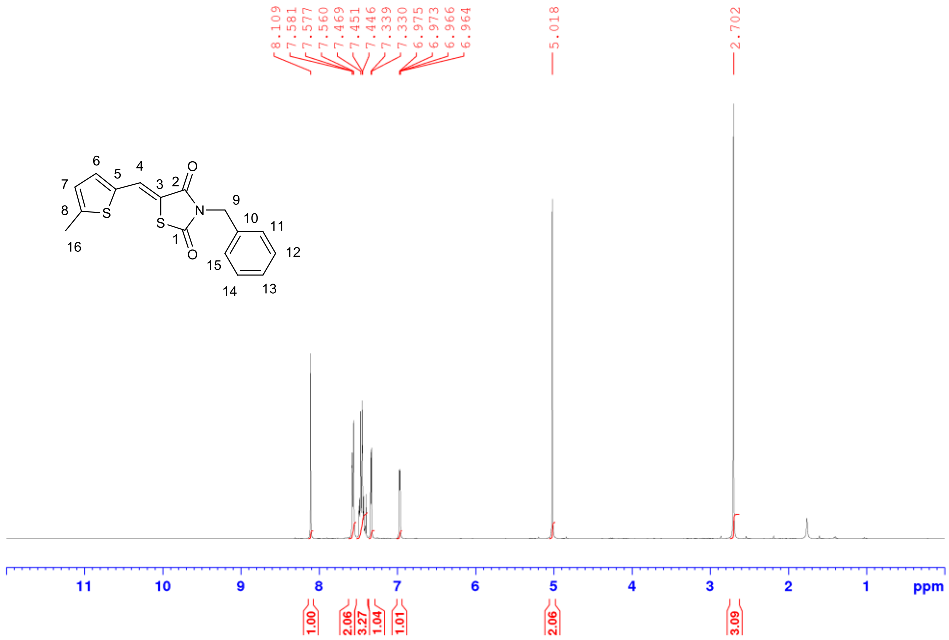

Figure S34. The ^{1}H NMR spectrum of **19e.**

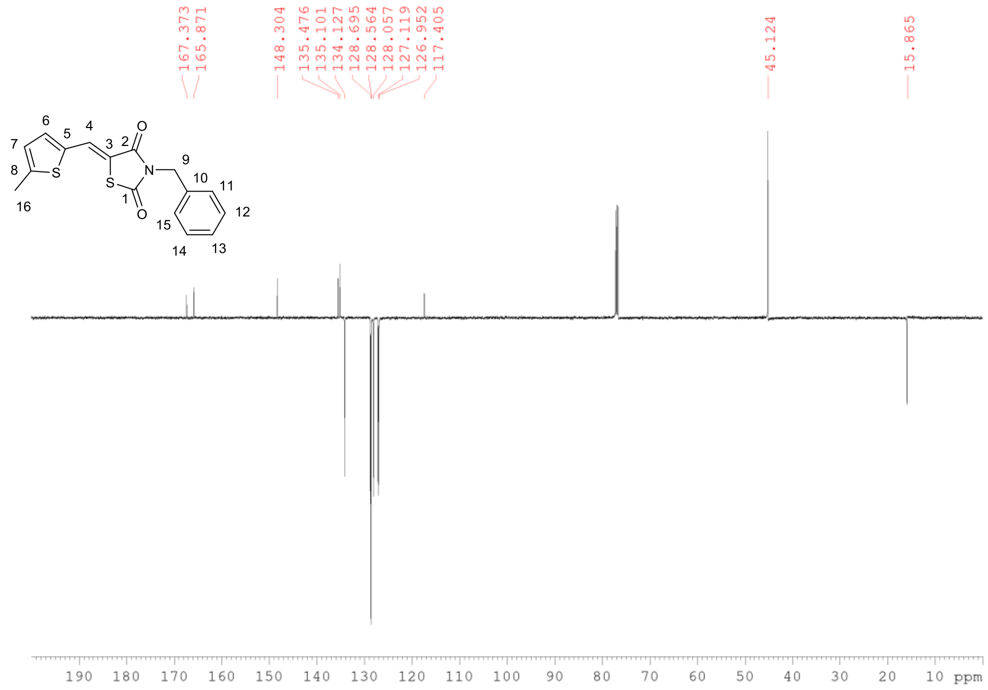

Figure S35. The ^{13}C NMR spectrum of **19e.**

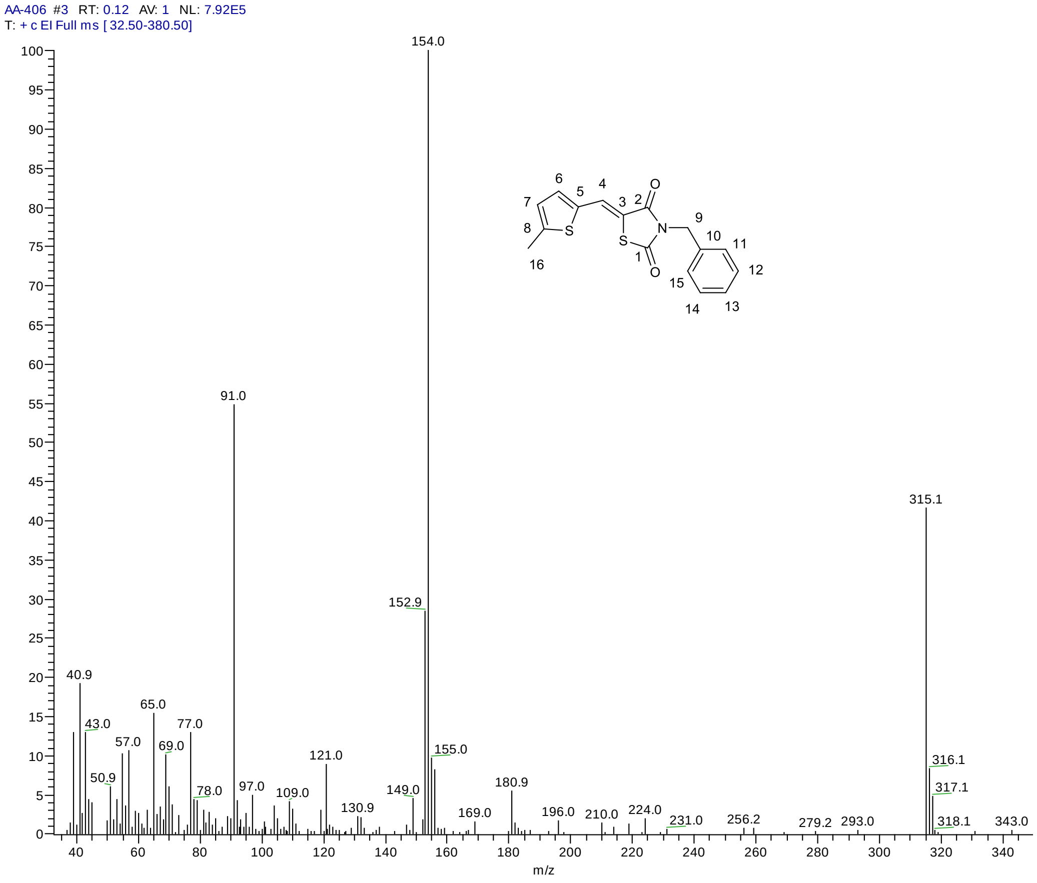

Figure S36. The DFS spectrum of **19e.**

Compound **20e**.

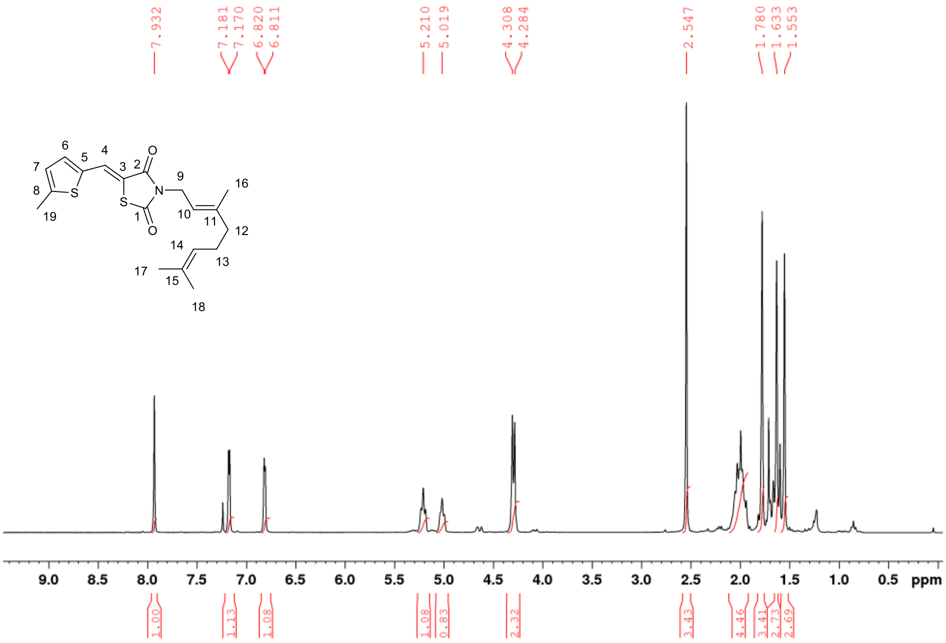

Figure S37. The ^{1}H NMR spectrum of **20e.**

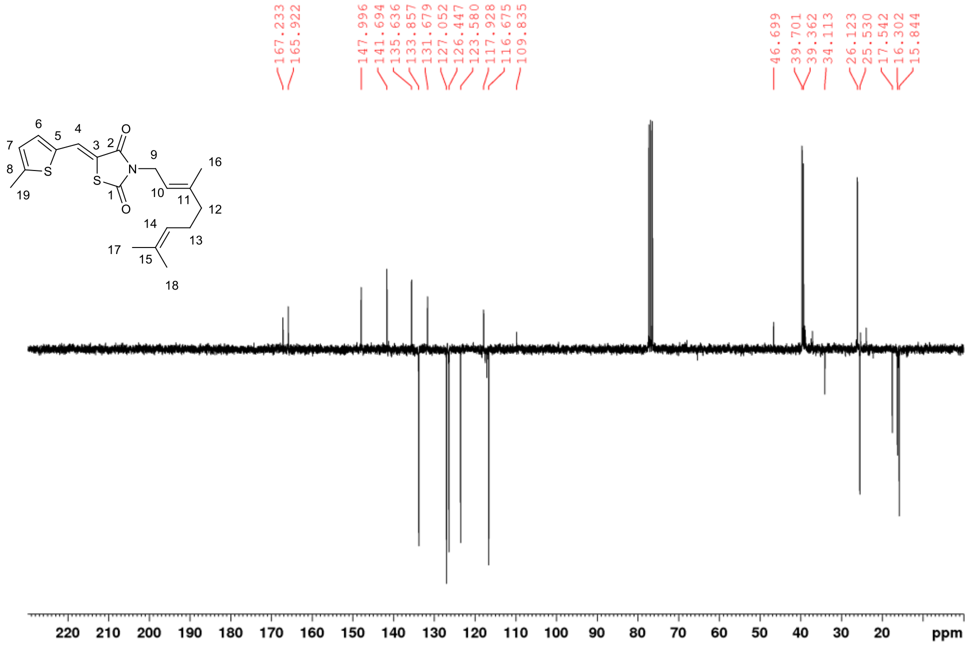

Figure S38. The ^{13}C NMR spectrum of **20e.**

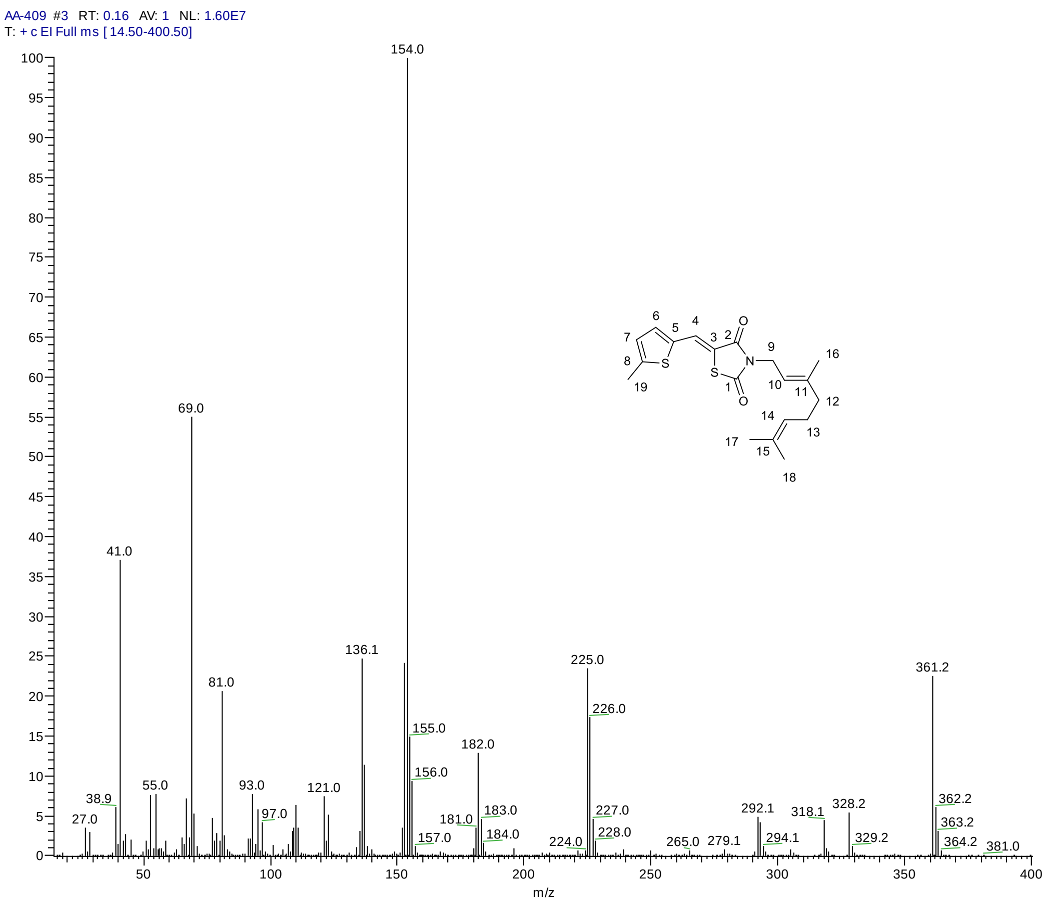

Figure S39. The DFS spectrum of **20e.**

Compound **21e**.

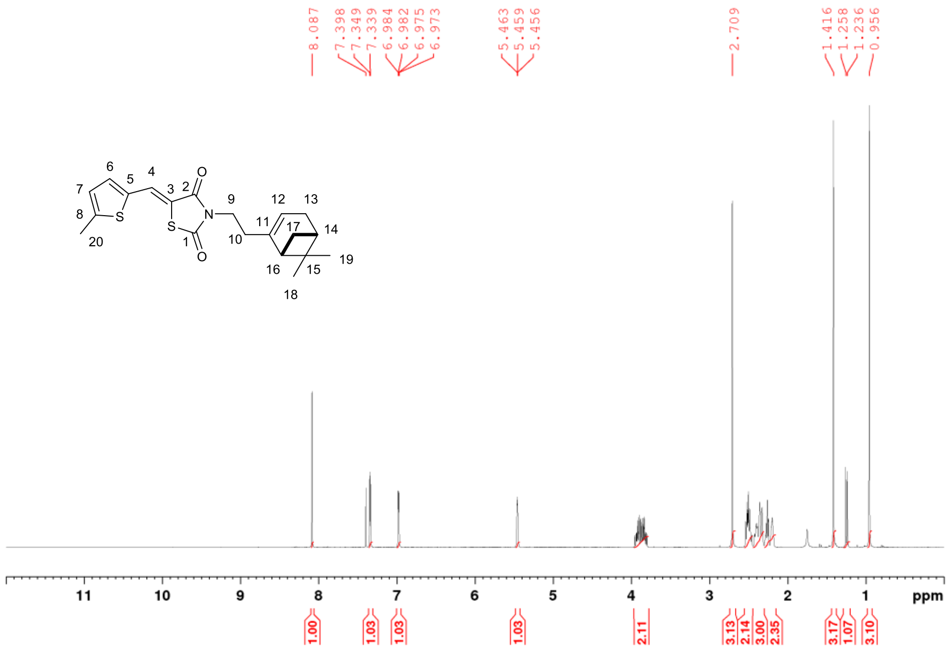

Figure S40. The ^{1}H NMR spectrum of **21e.**

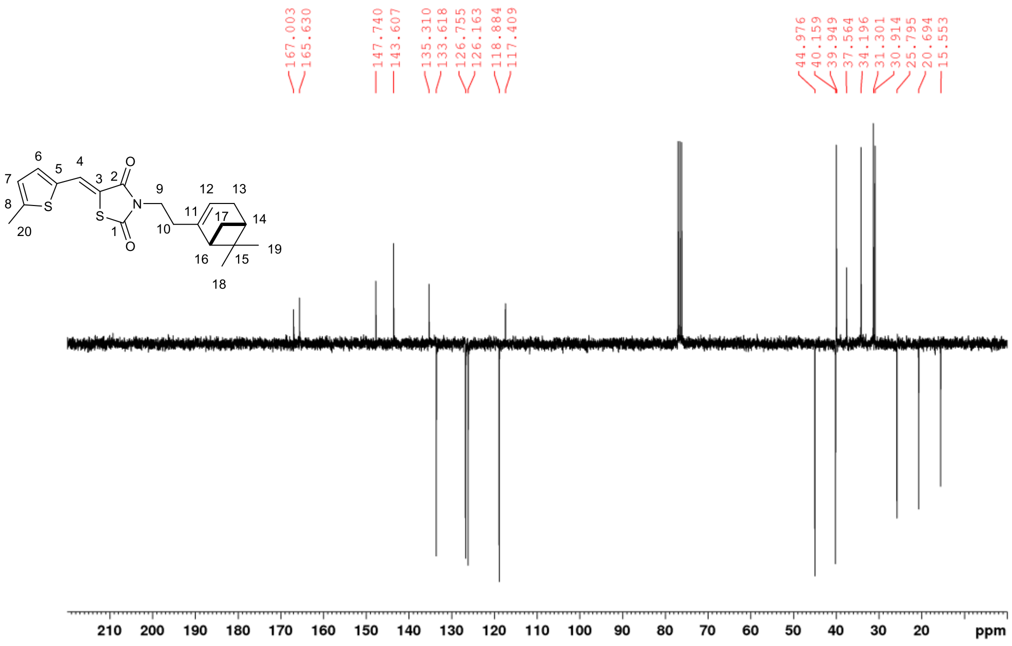

Figure S41. The ^{13}C NMR spectrum of **21e.**

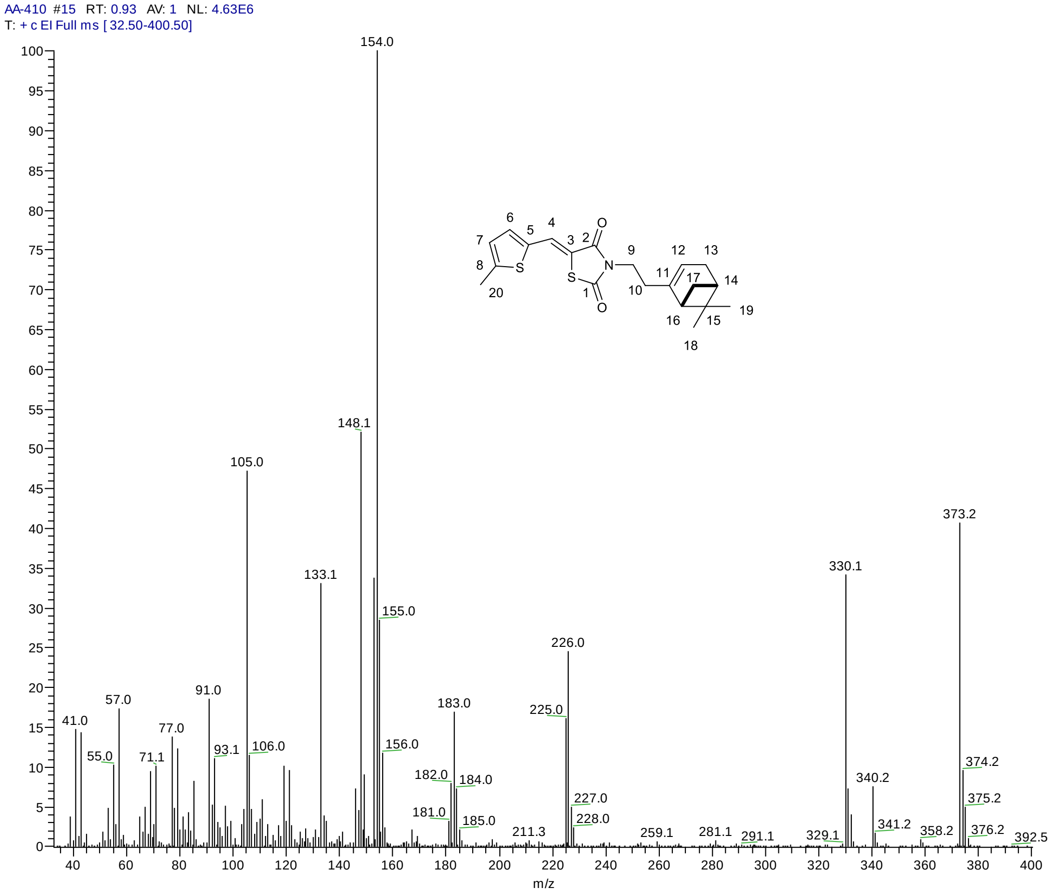

Figure S42. The DFS spectrum of **21e.**

Compound **19f**.

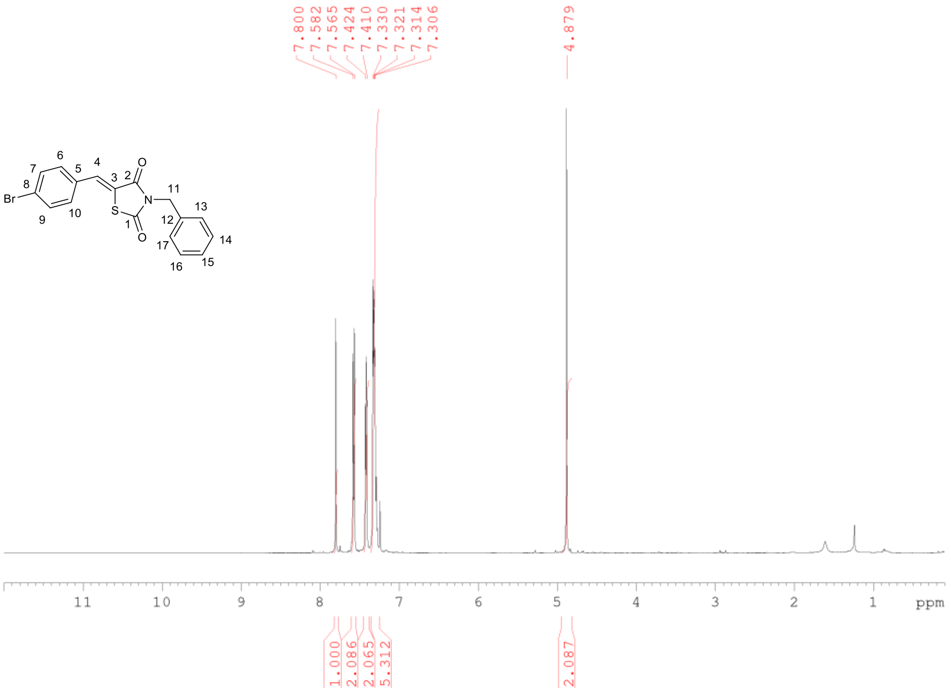

Figure S43. The ^{1}H NMR spectrum of **19f.**

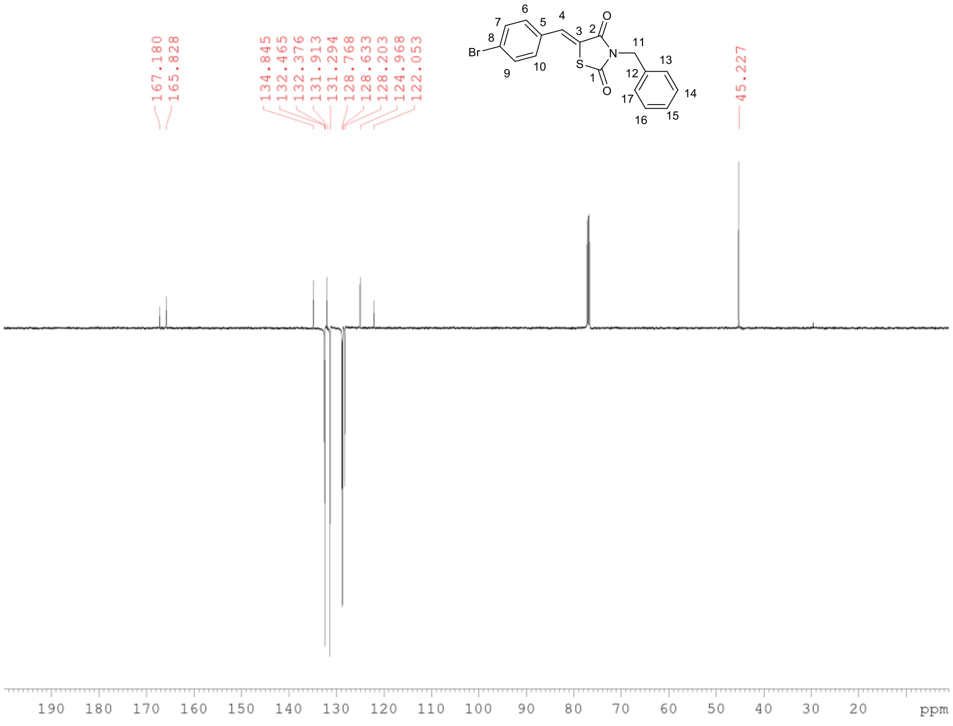

Figure S44. The ^{13}C NMR spectrum of **19f.**

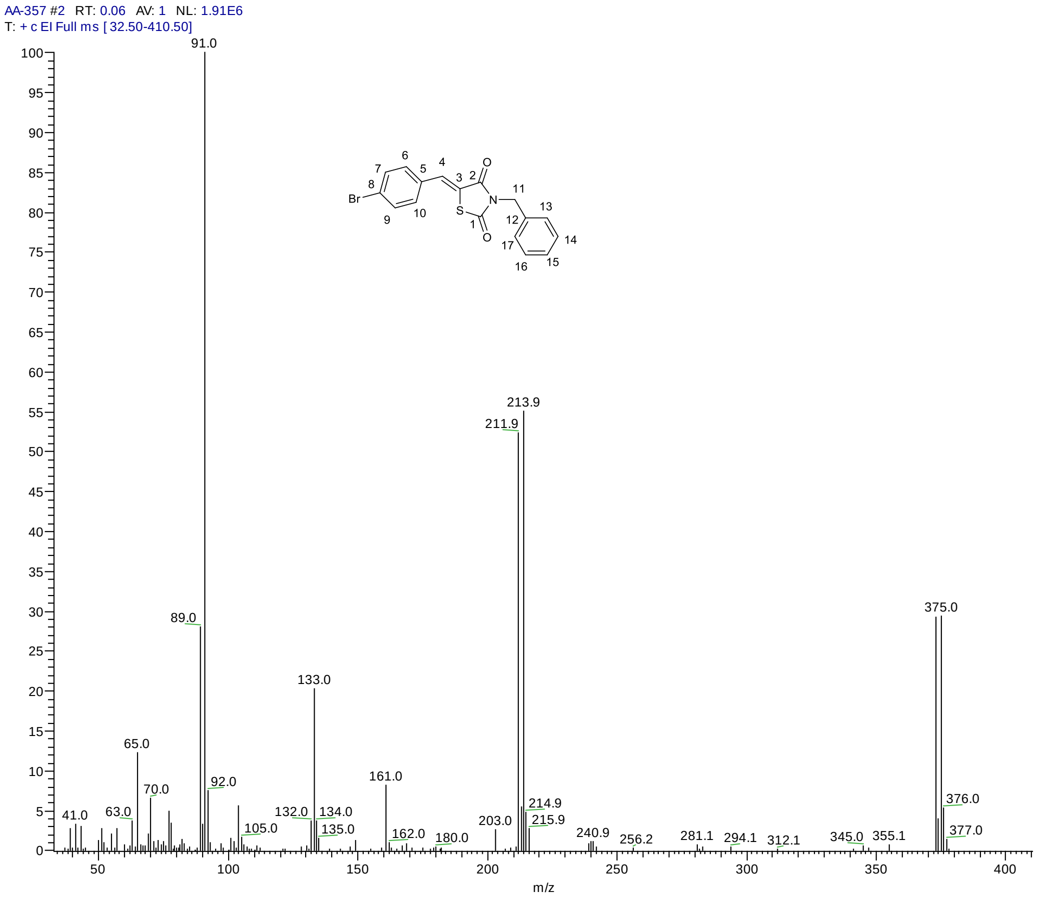

Figure S45. The DFS spectrum of **19f.**

Compound **20f**.

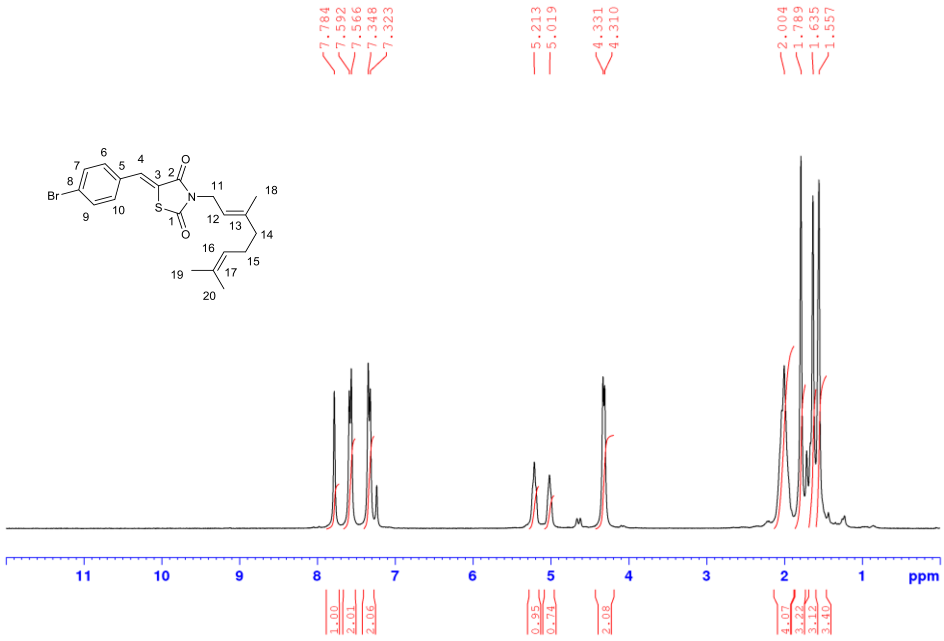

Figure S46. The ¹H NMR spectrum of **20f.**

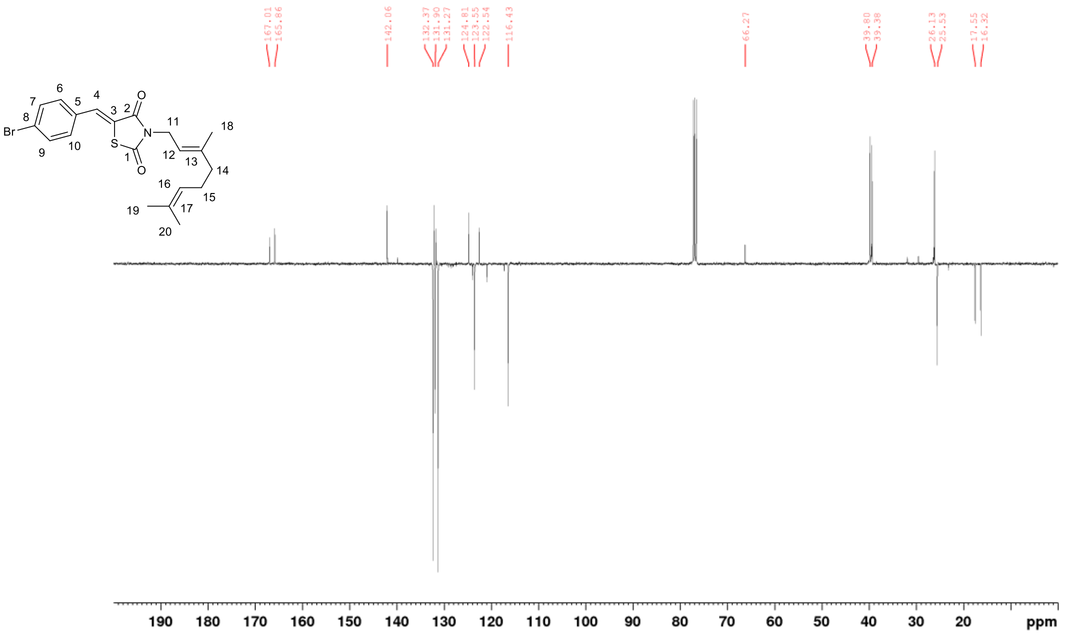

Figure S47. The ^{13}C NMR spectrum of **20f.**

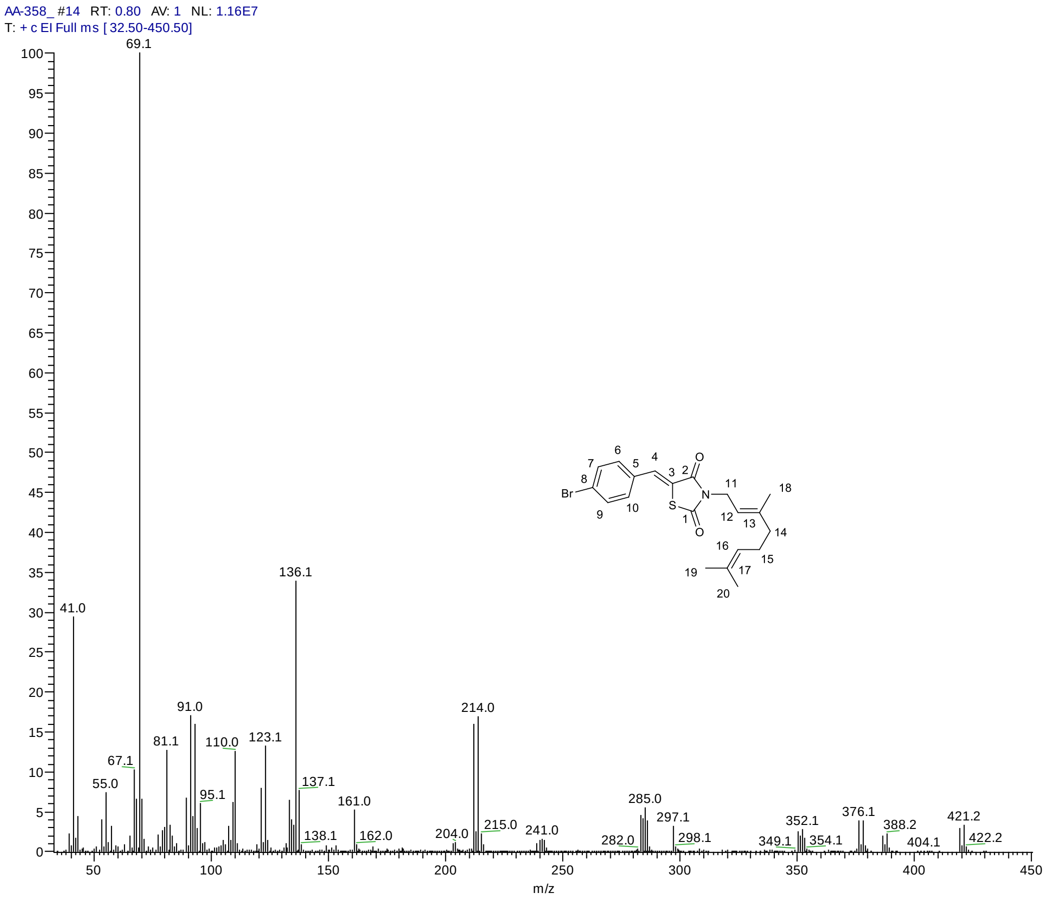

Figure S48. The DFS spectrum of **20f.**

Compound **21f**.

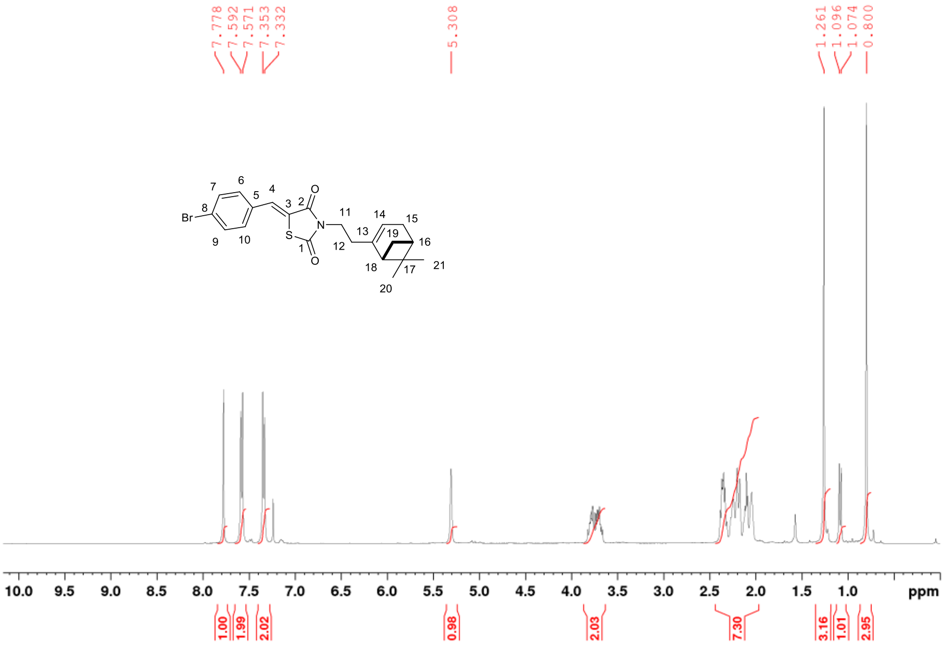

Figure S49. The ^{1}H NMR spectrum of **21f.**

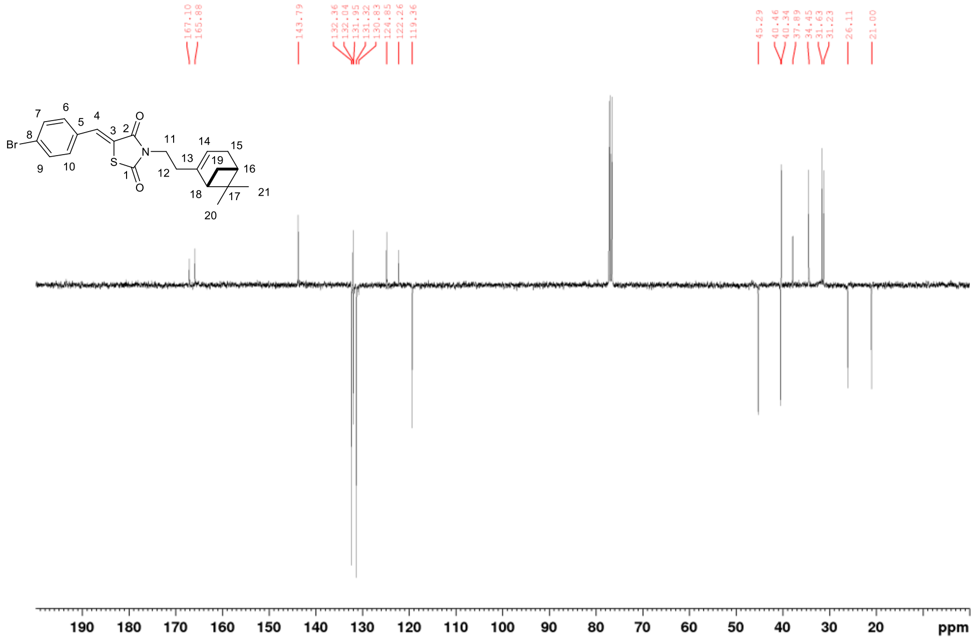

Figure S50. The ^{13}C NMR spectrum of **21f.**

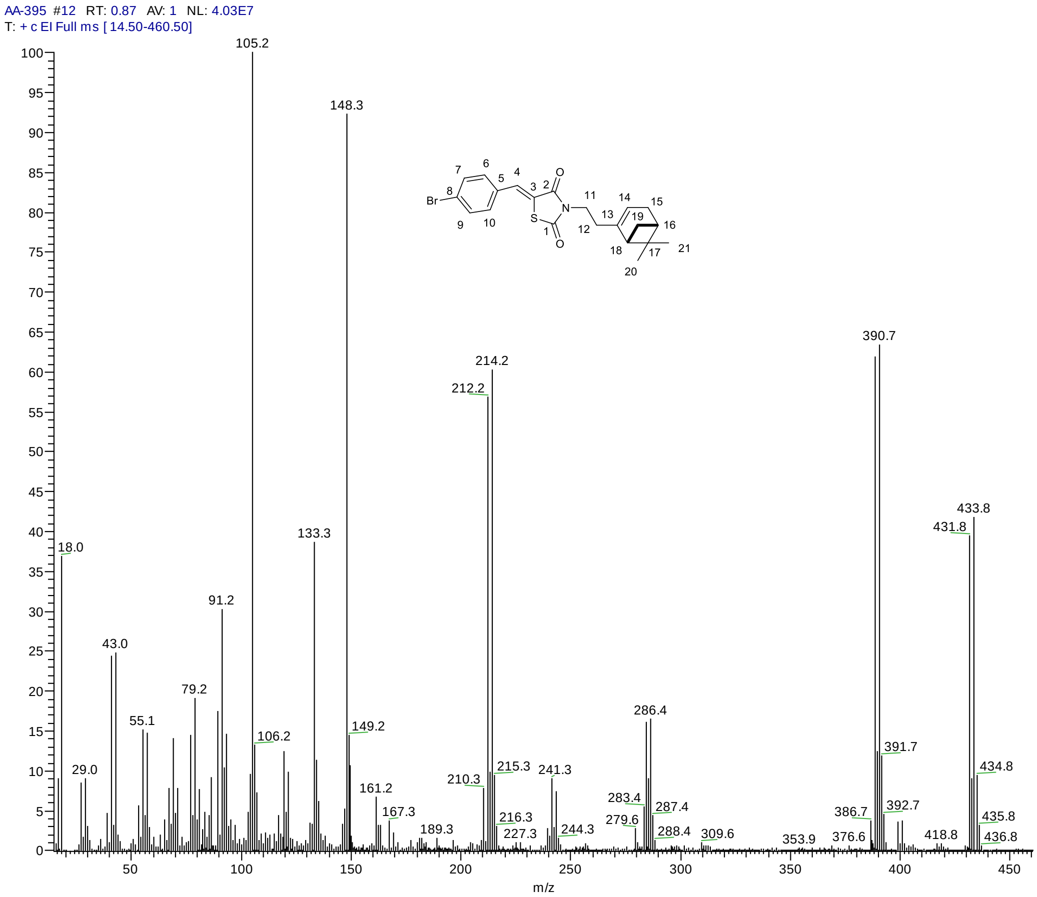

Figure S51. The DFS spectrum of **21f.**

Compound **21g**.

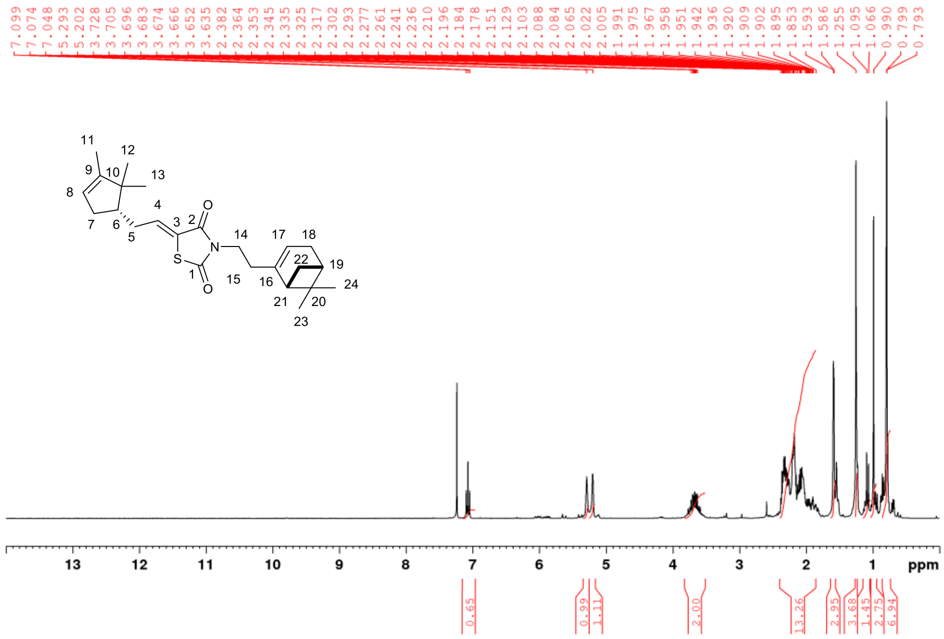

Figure S52. The ^{1}H NMR spectrum of **21g.**

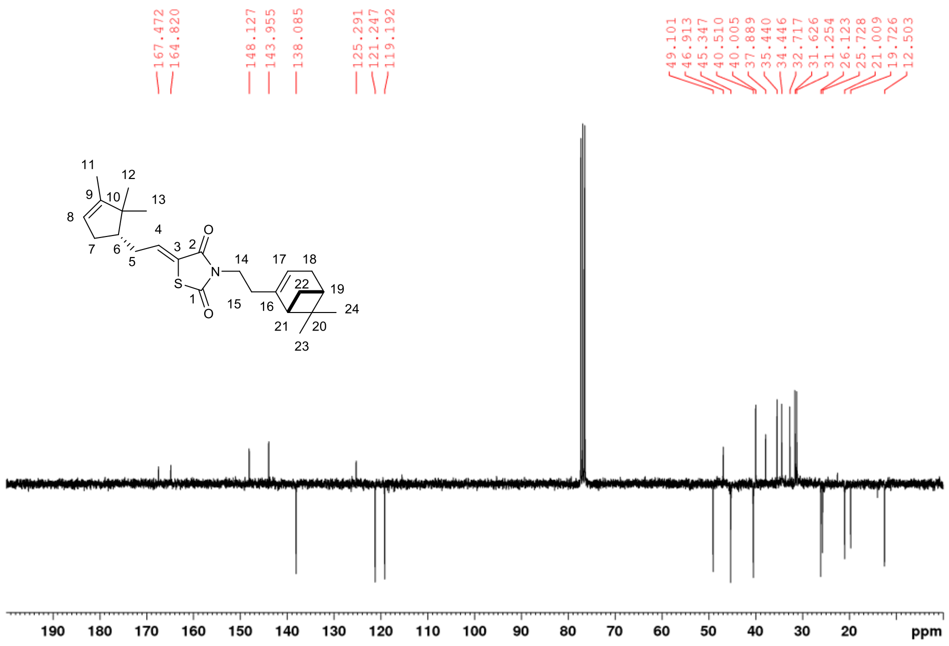

Figure S53. The ^{13}C NMR spectrum of **21g.**

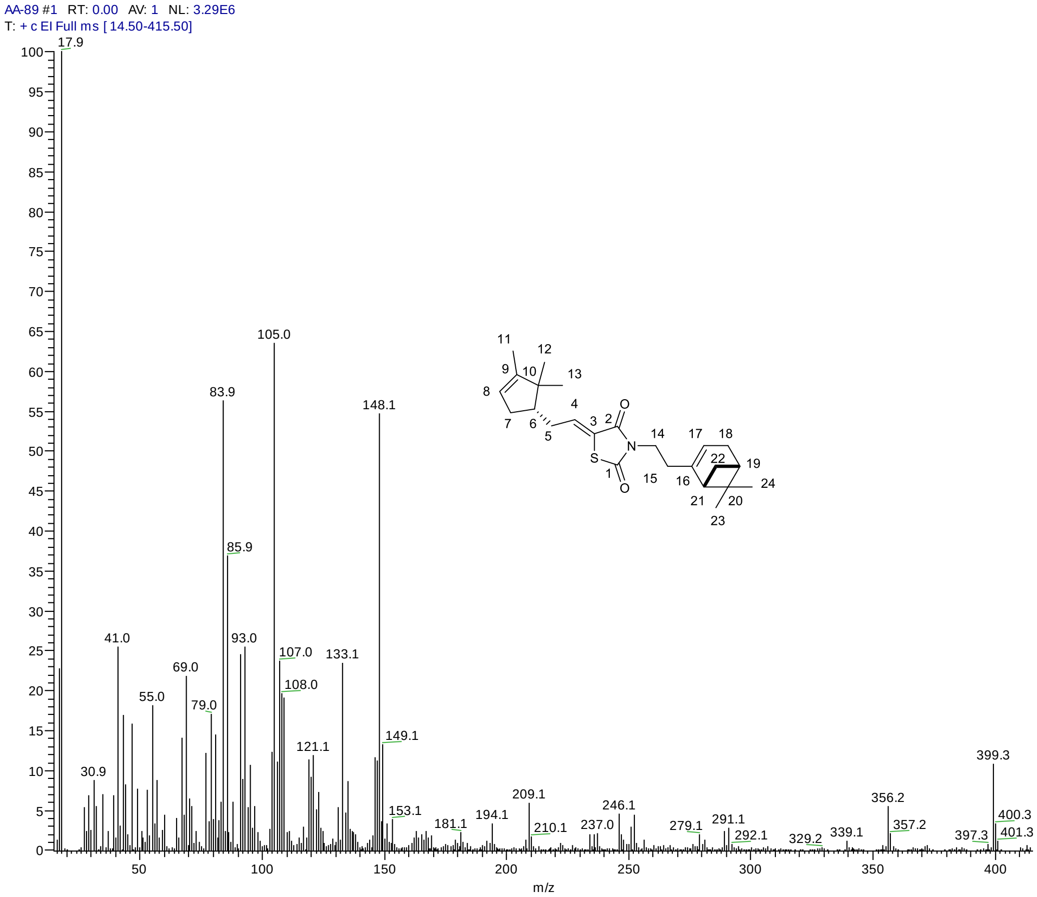

Figure S54. The DFS spectrum of **21g.**

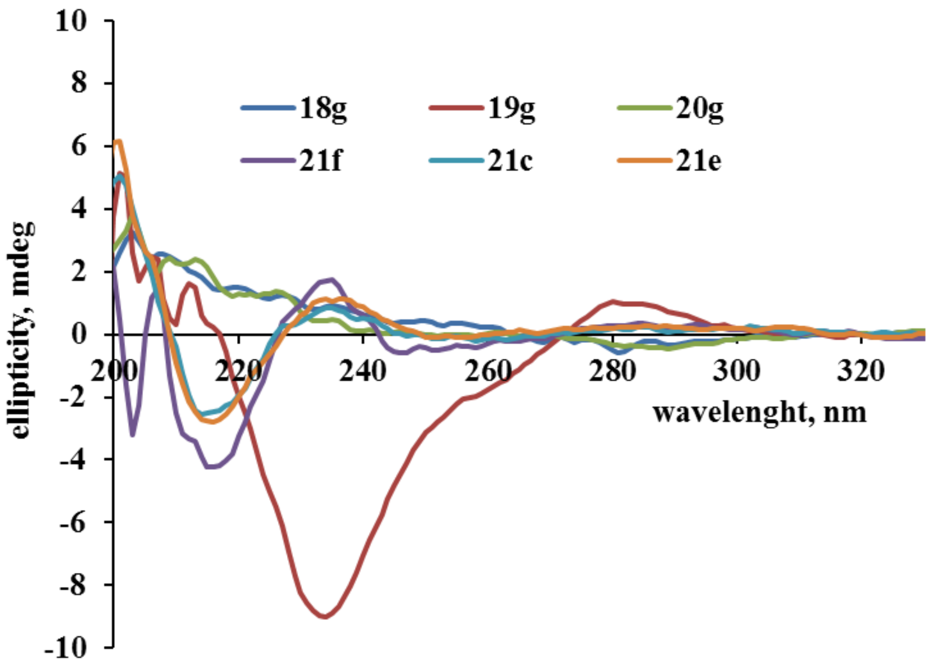

Figure S55. Circular dichroism spectroscopy experiments.

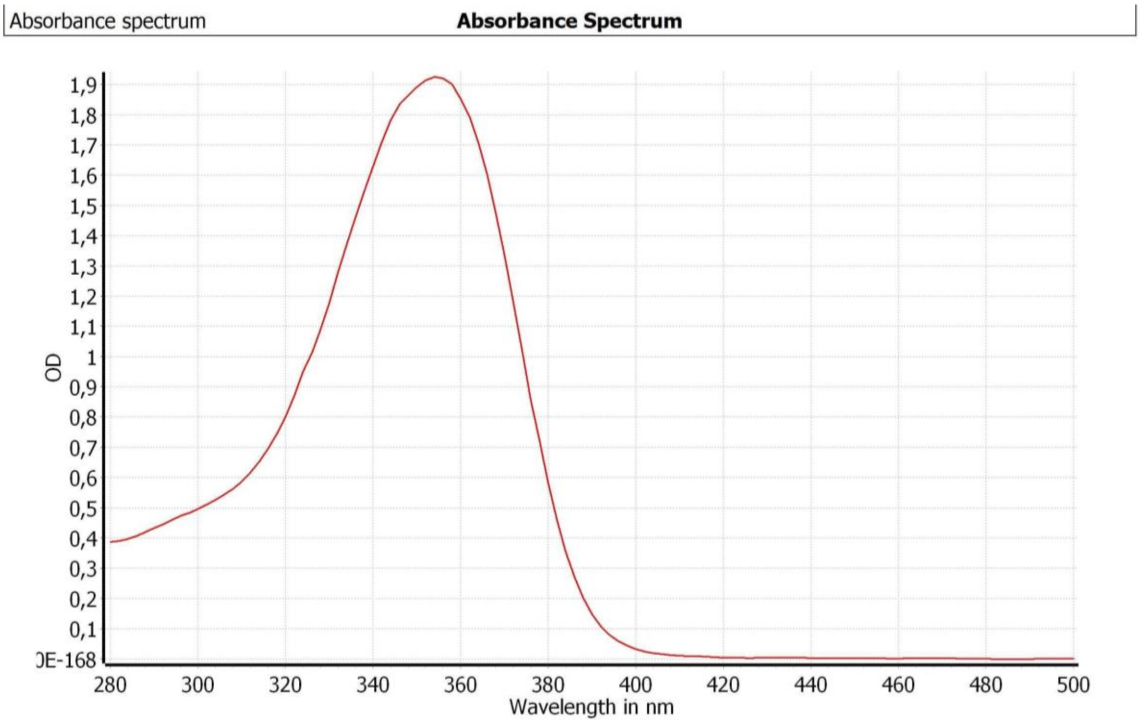

Figure S56. The UV-vis absorbtion spectrum of **18a**.

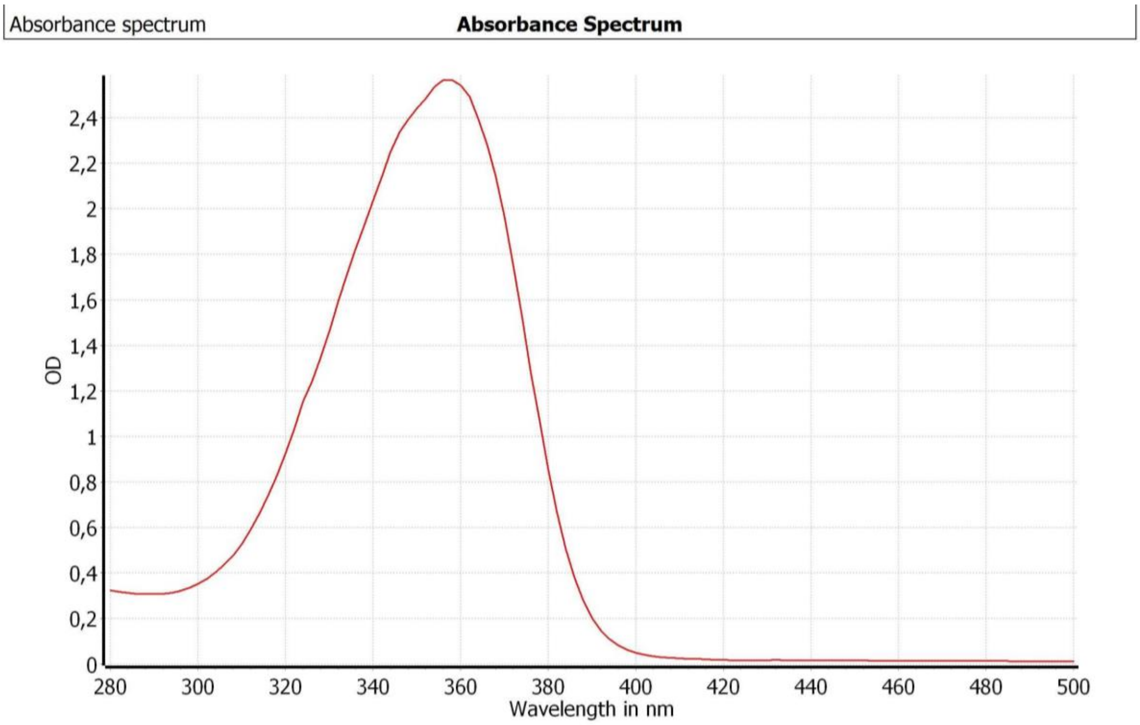

Figure S57. The UV-vis absorbtion spectrum of **19a**.

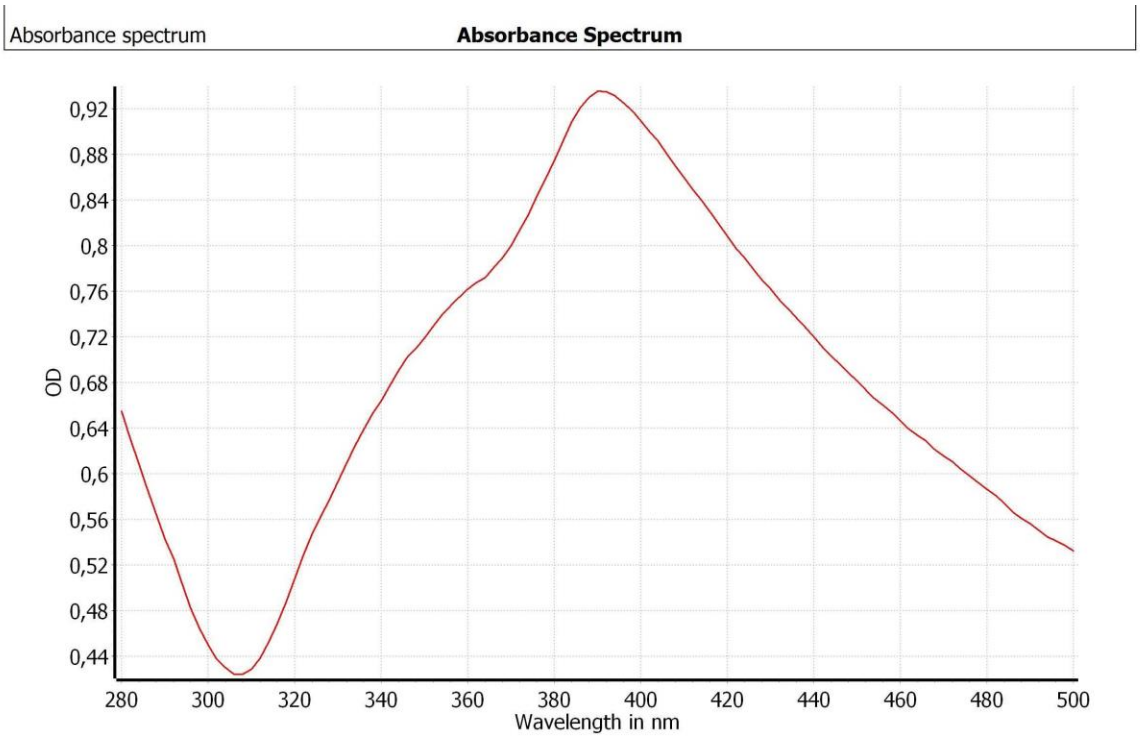

Figure S58. The UV-vis absorbtion spectrum of **20a**.

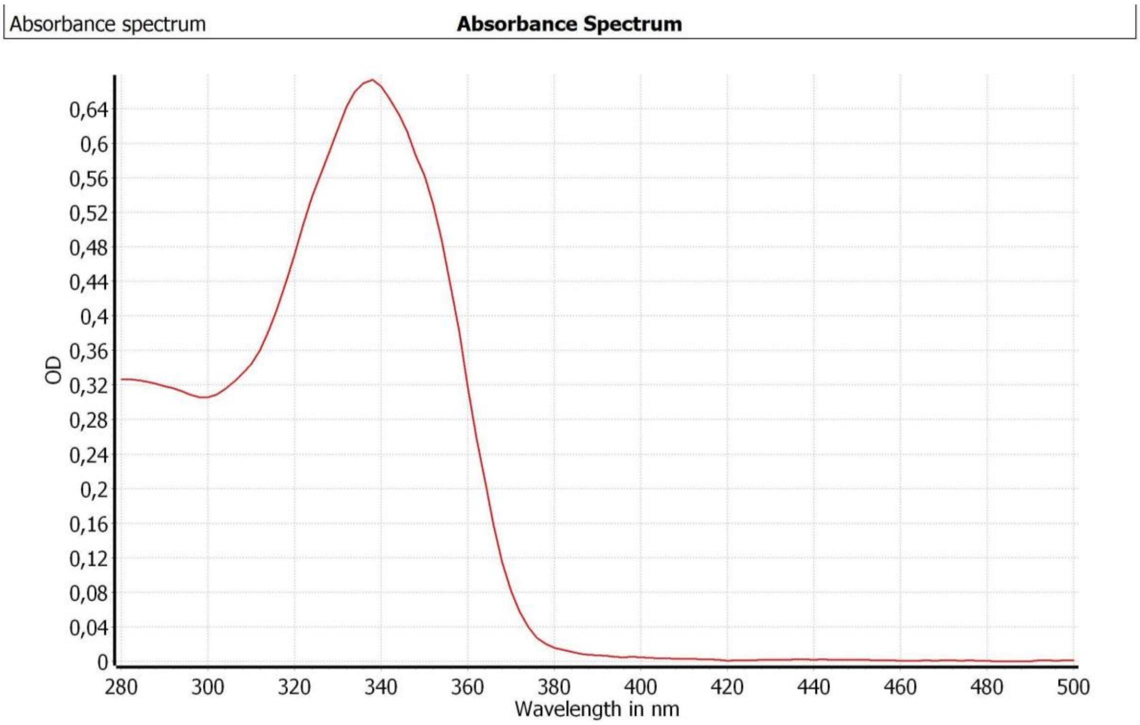

Figure S59. The UV-vis absorbtion spectrum of **18f**.

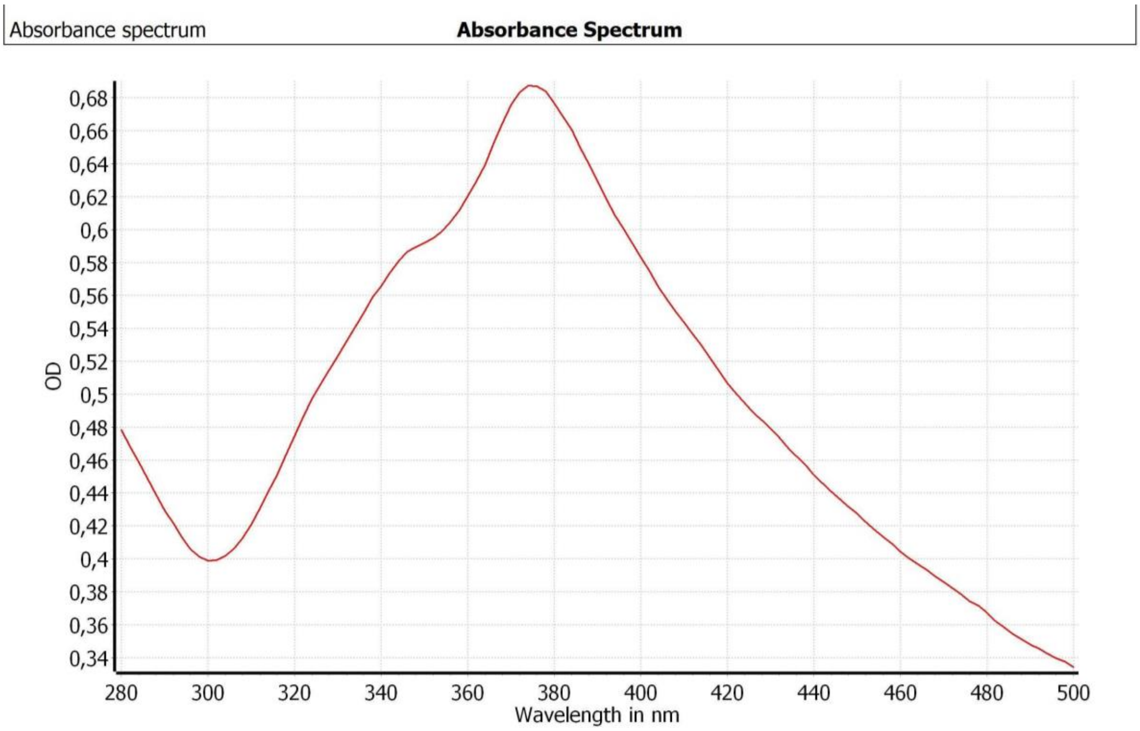

Figure S60. The UV-vis absorbtion spectrum of **19f**.

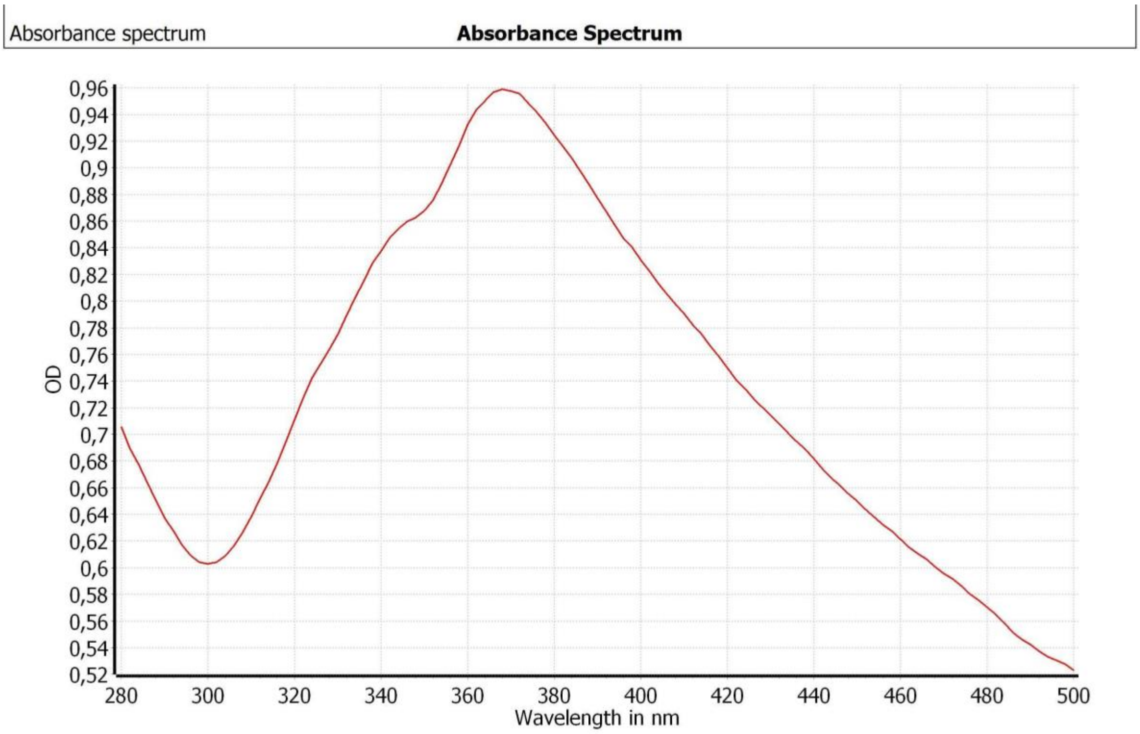

Figure S61. The UV-vis absorbtion spectrum of **20f**.

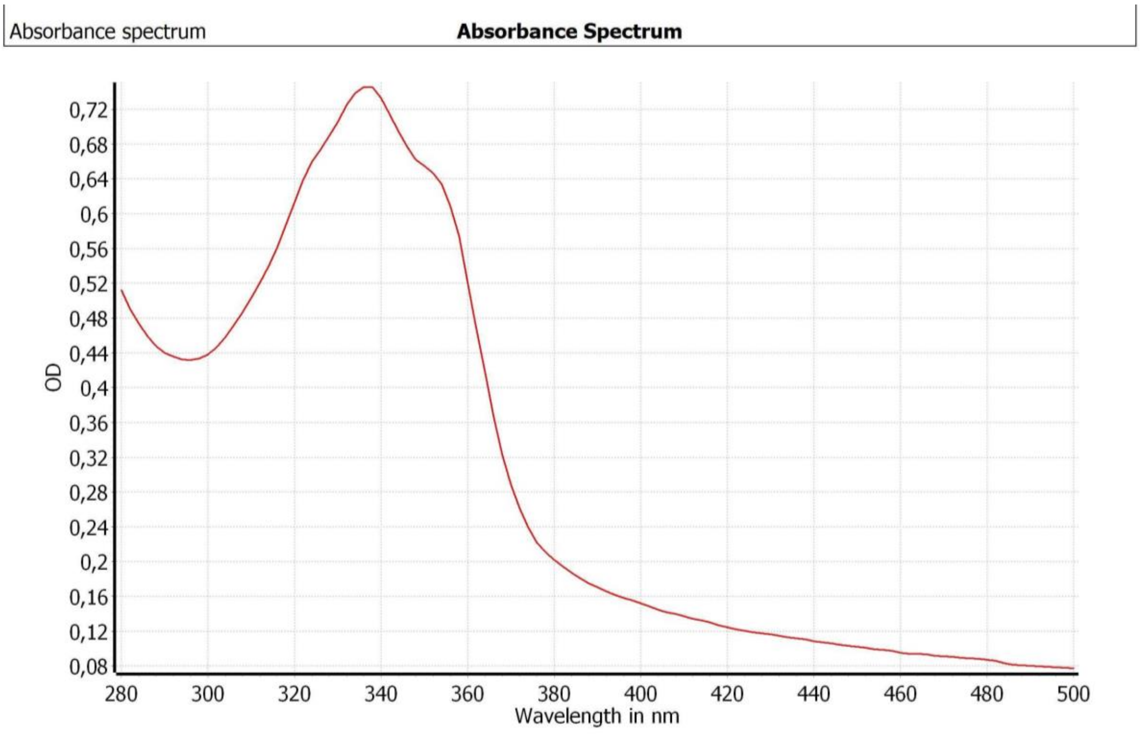

Figure S62. The UV-vis absorbtion spectrum of **21f**.

Table S1. The binding affinities as predicted by the scoring functions used to the catalytic TDP1 binding pocket and their measured IC_{50} values.

Ligand	ASP	ChemPLP	CS	GS	IC_{50}, μM
**18a**	28.5	48.6	29.7	43.4	> 100
**18b**	23.2	46.9	24.4	41.0	1.1 ± 0.2
**18c**	22.9	43.0	24.6	39.3	> 100
**18d**	24.1	45.2	26.4	40.1	> 100
**18e**	22.7	44.8	24.6	40.5	> 100
**18f**	27.5	46.4	28.4	47.7	> 100
**18g**	30.1	60.1	27.4	48.7	> 100
**19a**	31.9	52.2	25.8	50.8	> 100
**19b**	31.1	57.3	24.1	52.4	1.9 ± 0.3
**19c**	28.7	49.8	24.8	52.3	> 100
**19d**	28.5	49.9	22.9	52.9	> 100
**19e**	30.2	47.7	25.6	52.1	> 100
**19f**	31.2	55.9	27.2	51.7	1.6 ± 0.3
**19g**	33.6	54.6	27.4	52.5	2.4 ± 1.1
**20a**	32.2	61.7	27.4	54.4	3.4 ± 0.1
**20b**	31.1	64.0	26.2	57.9	> 100
**20c**	31.0	60.3	25.0	57.0	> 100
**20d**	32.5	63.3	24.8	57.1	0.65 ± 0.07
**20e**	30.6	64.9	25.7	56.2	2.9 ± 0.1
**20f**	32.9	67.6	27.7	59.7	> 100
**20g**	32.2	65.3	30.4	63.7	4.1 ± 0.1
**21a**	33.0	56.9	26.8	51.9	> 100
**21b**	29.1	58.6	26.9	52.2	2.5 ± 0.4
**21c**	31.4	57.6	25.8	55.1	> 100
**21d**	31.2	62.4	25.8	57.9	0.55 ± 0.07
**21e**	31.2	60.5	27.1	59.1	2.3 ± 0.1
**21f**	31.3	60.6	28.3	54.1	> 100
**21g**	34.1	68.0	30.1	59.6	2.4 ± 0.1

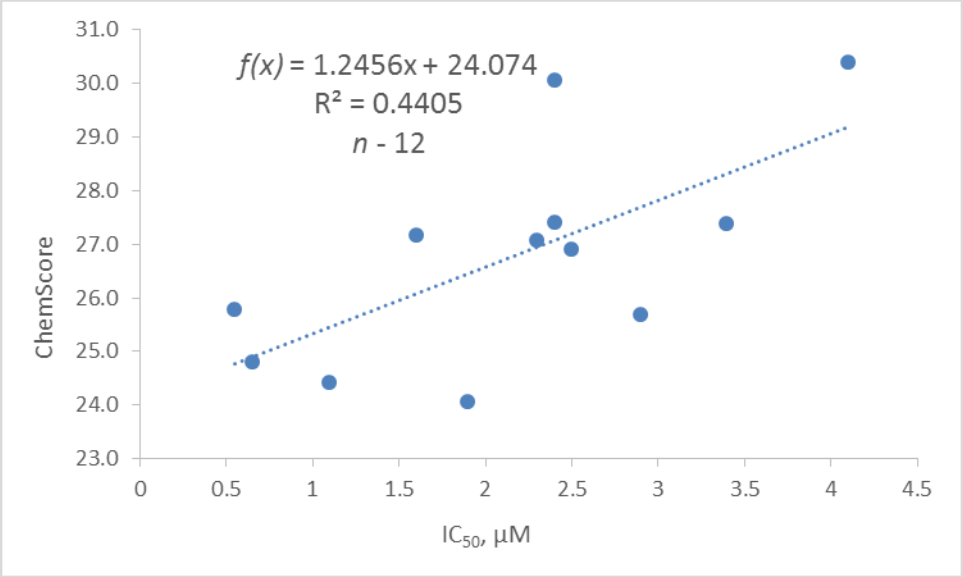

Figure S63. The correlation plot of measured IC_{50} values against their CS counterparts.

Table S2. The molecular descriptors and their corresponding Known Drug Indexes 2a and 2b ($KDI_{2a/2b}$).

	RB	MW	HD	HA	Log P	PSA	KDI_{2A}	KDI_{2B}	IC_{50}, µM
**18a**	2	195.2	1	3.5	0.9	83.7	4.38	0.10	> 100
**18b**	2	290.1	1	3	2.0	73.2	5.00	0.31	1.1 ± 0.2
**18c**	2	211.3	1	3	1.4	73.2	4.48	0.13	> 100
**18d**	2	290.1	1	3	2.0	73.2	5.00	0.31	> 100
**18e**	2	225.3	1	3	1.7	73.2	4.61	0.17	> 100
**18f**	2	284.1	1	3	2.0	76.4	4.98	0.30	> 100
**18g**	3	251.3	1	3	2.5	77.0	4.98	0.29	> 100
**19a**	4	285.3	0	3.5	3.3	63.4	4.96	0.28	> 100
**19b**	4	380.3	0	3	4.6	54.4	4.80	0.23	1.9 ± 0.3
**19c**	4	301.4	0	3	4.0	54.5	4.77	0.22	> 100
**19d**	4	380.3	0	3	4.6	54.4	4.80	0.22	> 100
**19e**	4	315.4	0	3	4.3	54.3	4.75	0.22	> 100
**19f**	4	374.3	0	3	4.6	54.6	4.78	0.22	1.6 ± 0.3
**19g**	5	341.5	0	3	5.1	60.0	4.65	0.18	2.4 ± 1.1
**20a**	7	331.4	0	3.5	4.9	66.6	4.61	0.18	3.4 ± 0.1
**20b**	7	426.4	0	3	6.0	59.4	4.14	0.08	> 100
**20c**	7	347.5	0	3	5.4	59.3	4.38	0.13	> 100
**20d**	7	426.4	0	3	6.0	59.3	4.14	0.08	0.65 ± 0.07
**20e**	7	361.5	0	3	5.7	59.4	4.31	0.11	2.9 ± 0.1
**20f**	7	420.4	0	3	6.0	59.3	4.14	0.08	> 100
**20g**	8	387.6	0	3	6.5	60.6	3.97	0.05	4.1 ± 0.1
**21a**	5	343.4	0	3.5	4.6	67.6	4.93	0.27	> 100
**21b**	5	438.4	0	3	5.7	60.5	4.37	0.11	2.5 ± 0.4
**21c**	5	359.5	0	3	5.1	60.4	4.68	0.18	> 100
**21d**	7	426.4	0	3	6.0	59.3	4.14	0.08	0.55 ± 0.07
**21e**	5	373.5	0	3	5.4	59.7	4.60	0.16	2.3 ± 0.1
**21f**	5	432.4	0	3	6.0	60.7	4.33	0.10	> 100
**21g**	6	399.6	0	3	6.3	59.7	4.28	0.08	2.4 ± 0.1

Table S3. Definition of lead-like, drug-like and Known Drug Space (KDS) in terms of molecular descriptors. The values given are the maxima for each descriptor for the volumes of chemical space used.

	**Lead-like Space**	**Drug-like Space**	**Known Drug Space**
Molecular weight (g mol^{-1})	300	500	800
Lipophilicity (Log P)	3	5	6.5
Hydrogen bond donors (HD)	3	5	7
Hydrogen bond acceptors (HA)	3	10	15
Polar surface area ($Å^2$) (PSA)	60	140	180
Rotatable bonds (RB)	3	10	17
